# Enhancing Controllability Robustness of *q*-Snapback Networks through Redirecting Edges

**DOI:** 10.34133/2019/7857534

**Published:** 2019-08-04

**Authors:** Yang Lou, Lin Wang, Guanrong Chen

**Affiliations:** ^1^City University of Hong Kong, Hong Kong; ^2^Shanghai Jiao Tong University, Shanghai 200240, China

## Abstract

The well-known small-world network model was established by randomly* rewiring* edges, aiming to enhance the synchronizability of an undirected nearest-neighbor regular network. This paper demonstrates via extensive numerical simulations that randomly* redirecting* edges could enhance the robustness of the network controllability for directed snapback networks against both random and intentional node-removal and edge-removal attacks.

## 1. Introduction

Network science has seen rapid development since the late 1990s and has gained popularity in recent years. With the idea of* randomly connecting* edges between nodes developed in the classical random-graph theory [1], the small-world network model [2] was established by* randomly rewiring* edges, aiming to enhance the synchronizability of an undirected nearest-neighbor regular network. Thereafter, another splash was made by the proposal of a scale-free network model [3] based on* randomly attaching* edges with preference. This paper suggests a modified* q*-snapback network model by* randomly redirecting* edges of the original* q*-snapback networks [4] and further studies its robustness of the network controllability against both random and intentional node-removal and edge-removal attacks.

Recently, the network controllability has become a focal topic in network science investigation [5–13], where the* controllability* means the ability of the network in changing its state from any initial position to any targeted position under a certain control input within a finite duration of time. Meanwhile, malicious attacks on complex networks are another concerned issue in network science studies [14–18]. Reportedly, intentional degree-based node attacks are more effective than random attacks on network structural controllability over directed random-graph (RG) networks and directed scale-free (SF) networks [19]. Both random and intentional edge-removal attacks have been studied by many. In [20], it was pointed out that intentional edge-removal attack by removing highly-loaded edges is very effective in reducing the network controllability. It is also observed (e.g., in [21]) that intentional edge-based attacks are usually able to trigger cascading failures in SF networks, but not necessarily in RG networks. These motivated some recent in-depth studies of the robustness of network controllability [4]. In this regard, both random and intentional attacks as well as both node-removal and edge-removal attacks were investigated. In particular, it was observed that redundant edges, which are not included in any of the maximum matchings, can be rewired or redirected so as to possibly enlarge a maximum matching such that the needed number of control driver nodes is reduced [22, 23].

The present paper continues the currently active study on the robustness of network controllability against both random and intentional attacks based on either node-removal or edge-removal strategy. In doing so, a modified* q*-snapback network model based on the original* q*-snapback networks [4] is proposed. Under the new framework, a new approach of randomly redirecting edges of the modified* q*-snapback network is formulated and evaluated. It is found that for all modified* q*-snapback networks with different numbers of redirecting edges, the one with *q* = 0.5 is overall the best, which is also better than other comparable complex network models such as random-graph networks [24], multiplex congruence networks [25], random triangle networks [26], and random rectangle networks (to be formulated in this paper). By examining closely the structures of the modified* q*-snapback networks, it is found that they contain many 3-rings and 4-rings, which are beneficial for enhancing the robustness of the network controllability, consistent with the observation reported in [4].

The rest of the paper is organized as follows. In Section 2, some preliminaries are given, including a brief description of the* q*-snapback network model and the proposed strategy of redirecting edges in such a network.  Section 3 reports the main experimental results of the paper, describes various attack methods, defines a robustness measure, introduces the modified* q*-snapback network model, and presents detailed numerical simulation results with thorough comparisons. It also discusses the effects of degree distributions on the controllability robustness. Finally, Section 4 concludes the investigation.

## 2. Preliminaries

### 2.1. The* q*-Snapback Network Model

**Figure 1:**
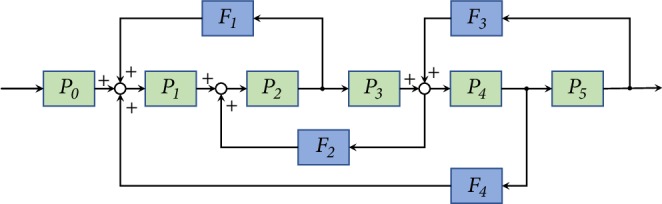
[4] Schematic of industrial assembly-line automation, where *P* represents a plant and *F* a feedback controller.

Resembling to the industrial assembly-line automation, as illustrated by Figure 1, the* q*-snapback network (QSN) model is established based on a backbone chain, with snapback edges determined by a probability *q* ∈ (0,1), which generates a directed network with multiring structure [4].

Briefly, the *q*-snapback network is constructed as follows. Start from a directed chain with nodes 1,2,…, *N*. Introduce a process index *r* = 2,3,…, *N*, and build the network with the following steps. For each node *i* = *r* + 1, *r* + 2,…, it connects backward to the previously appeared nodes *i* − *l* × *r* (*l* = 1,2,…, ⌊*i*/*r*⌋), with the same probability *q* ∈ (0,1). For *r* = 2, every node *i*  (≥3) connects forward to node *i* + 1  (≤*N*), and meanwhile, it connects backward to all previously appeared nodes *i* − 2, *i* − 4,…, with the same probability *q*. Then, the same process is repeated for *r* = 3, where it connects backward to the previously appeared nodes *i* − 3, *i* − 6,…, with 4 ≤ *i* ≤ *N*, according to the same probability uniformly. The construction continues for *r* = 4,5,…, *N*, until it cannot be processed any further. The resulting network is the *q*-snapback multiplex network of size *N*. Self-loops and multiple edges are naturally avoided in this generation method. It was shown [4] that the robustness of both state controllability and structural controllability of the *q*-snapback network model, against targeted and random node- and edge-removal attacks, is stronger than both the multiplex congruence network [25] and the generic scale-free network [27], due to its advantageous structure with many inherent chain- and loop-motifs. This is consistent with the observation that motifs are main components of communities in complex networks of many kinds [28].

### 2.2. Redirecting Edges

The proposed new network model is based on edge redirecting operations, as detailed in this subsection.

Each snapback edge is reversed with a probability *p*_*re*_ (*p*_*re*_ ∈ [0,1]), irrelative to the generating probability *q* ∈ (0,1), while the edges in the backbone chain are unchanged. When *p*_*re*_ = 0, it is the original QSN [25]; when *p*_*re*_ = 1, the result is a feedforward network.

**Figure 2:**
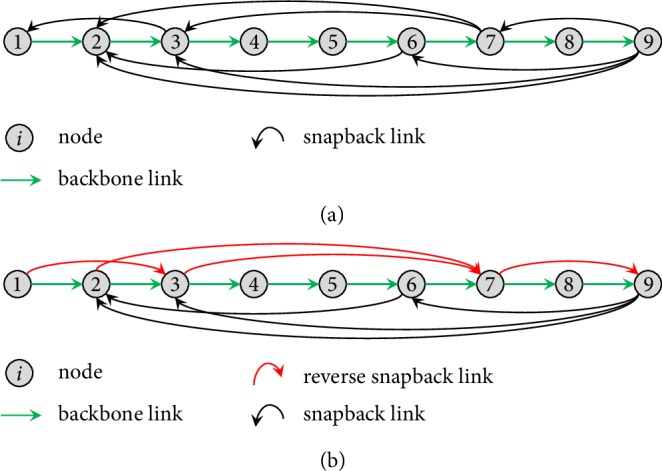
An example of QSN: (a) a randomly generated QSN with 9 nodes and 16 edges (*q* = 0.2); (b) QSN generated with *p*_*re*_ = 0.5, where the 4 redirected edges are colored red. For 3-ring motifs: in (a), there are two 3-rings, namely, 1-2-3 and 7-8-9. By redirecting edges, the original* local* rings are destroyed, but three new 3-rings are generated, namely, 2-7-9, 3-7-9, and 6-7-9.

As shown by the example in Figure 2, a few loops may be destroyed by such redirecting operations, but more new loops are formed, resulting in a network with more loops in the end. As illustrated later, this is exactly the main reason why the new model has better robustness of controllability than the original QSN model. It should be noted that the edges of the backbone chain will not be affected by the redirecting operation since none of them will be redirected. Here, the probability *p*_*re*_ is calculated by the number of redirected edges divided by the total number of snapback edges in the original QSN. For example, in Figure 2(a), the total number of snapback edges (black edges) is 8; when a redirecting operation with *p*_*re*_ = 0.5 is applied, 4 (red) edges might be redirected as illustrated by Figure 2(b).

QSN is constructed based on a backbone chain, where the nodes directly connected in the backbone chain are considered as local connected nodes, while the nodes connected by long distance snapback edges are nonlocal. In the QSN shown in Figure 2(a), there are two local 3-rings, namely, 1-2-3 and 7-8-9. Note that in the QSN model, due to its generation mechanism, only local rings can be formed. Notably, by randomly redirecting some edges (as shown in Figure 2(b)), these two local 3-rings will be broken down, but three new 3-rings will be formed: 2-7-9, 3-7-9, and 6-7-9. Thus, by performing redirecting, not only the number of 3-rings increases from two to three, but also the diversity of motifs increases, in the sense that nonlocal motifs are formed. This gives another reason why the new model has better robustness of controllability than the original QSN model, as further discussed in Section 3.

### 2.3. Expected Degree of Each Node

For a single layer (the *r*th layer, *r* = 1,…, *N* − 1) in a QSN, the expected outdegree (i.e., number of outgoing edges) and indegree (i.e., number of incoming edges) of each node can be calculated as follows:

(1) $$\begin{array}{c} d_{O}\left(i\right)=\left\{\begin{array}{cc} 1+\left\lfloor \frac{N-i}{r}\right\rfloor \cdot q\cdot p_{re}, & \text{for }i=1,2,\ldots ,r\\ 1+\left\lfloor \frac{i-1}{r}\right\rfloor \cdot q\cdot \left(1-p_{re}\right)+\left\lfloor \frac{N-i}{r}\right\rfloor \cdot q\cdot p_{re}, & \text{for }i=r+1,\ldots ,N-r\\ 1+\left\lfloor \frac{i-1}{r}\right\rfloor \cdot q\cdot \left(1-p_{re}\right), & \text{for }i=N-r+1,\ldots ,N-1\\ \left\lfloor \frac{i-1}{r}\right\rfloor \cdot q\cdot \left(1-p_{re}\right), & \text{for }i=N \end{array}\right. \end{array}$$

where *d*_*O*_(*i*) represents the expected outdegree of node *i*, and ⌊*x*⌋ is the floor function that returns the greatest integer less than or equal to *x*, and

(2) $$\begin{array}{c} d_{I}\left(i\right)=\left\{\begin{array}{cc} \left\lfloor \frac{N-1}{r}\right\rfloor \cdot q\cdot \left(1-p_{re}\right), & \text{for }i=1\\ 1+\left\lfloor \frac{N-i}{r}\right\rfloor \cdot q\cdot \left(1-p_{re}\right), & \text{for }i=2,\ldots ,r\\ 1+\left\lfloor \frac{N-i}{r}\right\rfloor \cdot q\cdot \left(1-p_{re}\right)+\left\lfloor \frac{i-1}{r}\right\rfloor \cdot q\cdot p_{re}, & \text{for }i=r+1,\ldots ,N-r\\ \left\lfloor \frac{i-1}{r}\right\rfloor \cdot q\cdot p_{re}, & \text{for }i,=N-r+1,\ldots ,N \end{array}\right. \end{array}$$

where *d*_*I*_(*i*) represents the expected indegree of node *i*, *i* = 1,2,…, *N*.

An illustrative example of the expected outdegree and indegree, calculated by (1) and (2), respectively, and the statistical outdegree and indegree averaged from 1000 QSNs, are given in the Supplementary Information ([Supplementary-material supplementary-material-1]). As for a multiplex QSN, the expected outdegree and indegree can be calculated, respectively, by

(3) $$\begin{array}{c} d_{O}^{M}\left(i\right)=\left\{\begin{array}{cc} 1+\overset{N}{\underset{j=2}{\sum }}P_{ji}\cdot p_{re}, & \text{for }i=1\\ 1+\overset{i-1}{\underset{j=1}{\sum }}P_{ij}\cdot \left(1-p_{re}\right)+\overset{N}{\underset{j=i+1}{\sum }}P_{ji}\cdot p_{re}, & \text{for }i=2,3,\ldots ,N-1\\ \overset{N-1}{\underset{j=1}{\sum }}P_{ij}\cdot \left(1-p_{re}\right), & \text{for }i=N \end{array}\right. \end{array}$$

and

(4) $$\begin{array}{c} d_{I}^{M}\left(i\right)=\left\{\begin{array}{cc} \overset{N}{\underset{j=2}{\sum }}P_{ji}\cdot \left(1-p_{re}\right), & \text{for }i=1\\ 1+\overset{N}{\underset{j=i+1}{\sum }}P_{ji}\cdot \left(1-p_{re}\right)+\overset{i-1}{\underset{j=1}{\sum }}P_{ij}\cdot p_{re}, & \text{for }i=2,3,\ldots ,N-1\\ 1+\overset{N-1}{\underset{j=1}{\sum }}P_{ij}\cdot p_{re}, & \text{for }i=N \end{array}\right. \end{array}$$

where *d*_*O*_^*M*^(*i*) and *d*_*I*_^*M*^(*i*) represent the expected outdegree and indegree of node *i* in the multiplex QSN. Note that an edge (*i*, *j*) could appear on different layers of a multiplex QSN. The probability that edge (*i*, *j*) exists on at least one layer, i.e., the probability of the existence of edge (*i*, *j*), is given by

(5) $$\begin{array}{c} P_{ij}=1-{\left(1-q\right)}^{\prod_{e=1}^{E}\left(x_{e+1}\right)} \end{array}$$

where *i*, *j* = 1,2,…, *N*, satisfying *i* − *j* = *a*_1_^*x*_1_^ · *a*_2_^*x*_2_^ ⋯ *a*_*E*_^*x*_*E*_^, with *a*_1_, *a*_2_,…, *a*_*E*_ being prime numbers, and (1 − *q*)^∏_*e*=1_^*E*^(*x*_*e*+1_)^ represents the probability that edge (*i*, *j*) does not exist on any layer of the multiplex network. Here, 1 means 1^0^.

## 3. Experimental Studies

An extensive experimental study is carried out to verify the effectiveness of the above-described edge redirecting operations in improving the robustness of network controllability. Firstly, QSN networks with different redirecting probabilities are compared. Then, the QSN generated with redirecting probability *p*_*re*_ = 0.5, which is the overall best-performing network among all the QSN variants, is evaluated and compared to other 4 typical network topologies.

### 3.1. Attack Methods

Attack methods used in simulation include node-removal and edge-removal. Node-removal attack removes the nodes one after another, until there is only one single node left. When a node is removed, all of its connected edges are removed together. In an edge-removal attack, edges are removed one after another, while no node is removed even if a node has become isolated. Thus, when the edge-removal attack is terminated, there are *N* isolated nodes left.

A total of 6 types of attacks are performed in simulations, as are summarized in Table 1. Here, the edge-degree is calculated by taking the geometric mean of the node-degrees of the two end nodes of an edge [15]. Thus, for a directed edge *A*_*ij*_, its edge-degree is $\sqrt{k_{i}^{in}\cdot k_{j}^{out}}$, where *k*_*i*_^*in*^ is the indegree of node *i* and *k*_*j*_^*out*^ is the outdegree of node *j*.

For all the intentional attacks listed in Table 1, the target (node or edge) with the largest betweenness or largest degree is updated after each node- or edge-removal. Here, as a common centrality measure in graph theory and network science, betweenness of a node or an edge is the sum of the shortest path-lengths that pass through the node or the edge.

### 3.2. Robustness Measure

The network controllability is measured by the density of the control-nodes *n*_*D*_, defined by

(6) $$\begin{array}{c} n_{D}\equiv \frac{N_{D}}{N}, \end{array}$$

where *N*_*D*_ is the number of external controllers (also called driver nodes) needed to retain the network controllability after the network had been attacked, and *N* is the network size. Note that *N* does not change during an edge-removal attack, but it would be reduced by a node-removal attack. Under this measure, the smaller the *n*_*D*_ is, the more robust the network controllability will be.

On the other hand, recall from computational network science that a network is considered to be sparse if the number of edges *M* (i.e., the number of nonzero elements of the adjacency matrix) is much smaller than the possible maximum number of edges, *M*_*max*_ (for a directed network, *M*_*max*_ = *N* · (*N* − 1)/2). Practically, if *M*/*M*_*max*_ ≤ 0.05, then it is considered a sparse network.

For state controllability, if the adjacency matrix *A* of the network is sparse, the number of driver nodes *N*_*D*_ can be calculated by [29]

(7) $$\begin{array}{c} N_{D}=\max\left\{1,N-rank\left(A\right)\right\}. \end{array}$$

As for structural controllability, the number of driver nodes *N*_*D*_ is determined by the number of elements in the maximum matching [5]:

(8) $$\begin{array}{c} N_{D}=\max\left\{1,N-\left|E^{*}\right|\right\}, \end{array}$$

where |*E*^*∗*^| is the cardinal number of elements in the maximum matching *E*^*∗*^. A maximum matching of a network is a matching that contains the largest possible number of edges, which cannot be further extended in the network.

For measuring the robustness of both state and structural controllabilities, the value of *n*_*D*_ is calculated according to (6) and recorded after each node or edge is removed.

The quantitative measure of controllability robustness [30] is used for comparison among different network topologies. It is a comparative measure that gives a comparison rank of multiple networks, which takes into account of each node- or edge-removal through the whole process.

**Figure 3:**
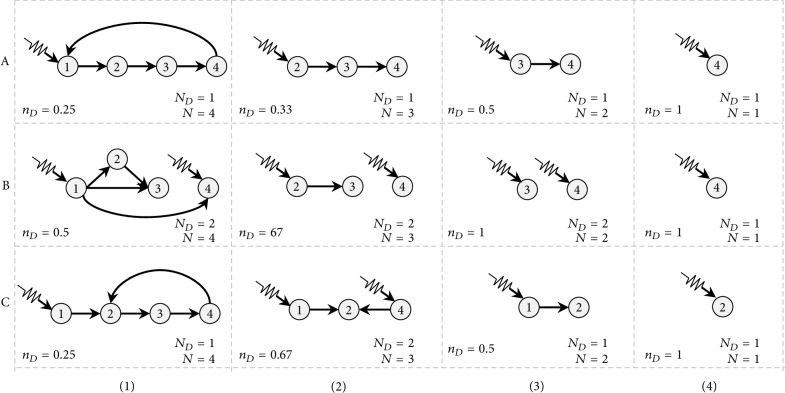
Three networks (A, B, and C) under a node-removal attack. The node-removal order for each network is based on the outdegrees, descending from large to small. When there is more than one node having the maximum outdegree, it randomly picks one among them. For networks A and B, the node-removal order is 1, 2, and 3; for network C, the order is 3, 4, and 1. The density of driver-nodes (*n*_*D*_), the number of nodes (*N*), and the number of minimal driver-nodes needed (*N*_*D*_) are also presented. The corresponding comparison is shown in Figure 4.

**Figure 4:**
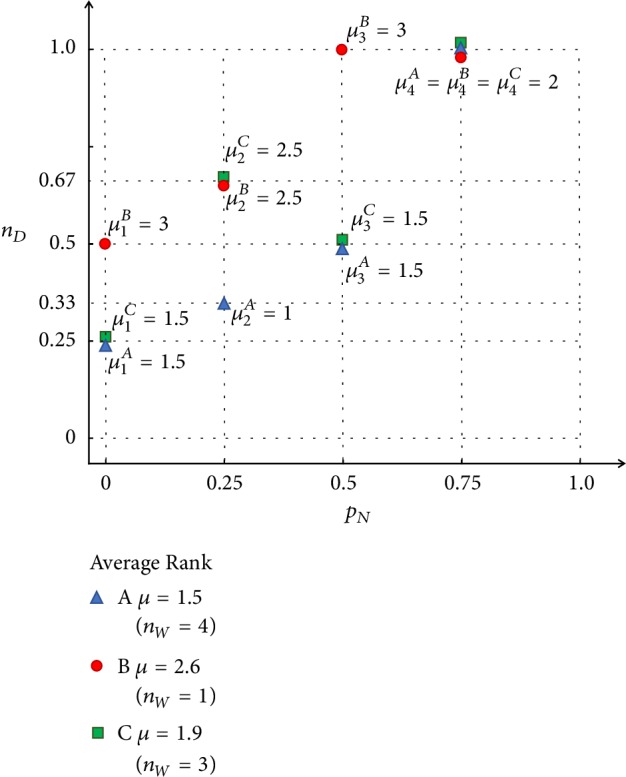
Density of driver-nodes (*n*_*D*_), as a function of the proportion of the removed nodes (*p*_*N*_). Initially, when no node has been removed (*p*_*N*_ = 0), both networks A and C require only 1 driver-node, while network B requires 2. When *p*_*N*_ = 0.75, meaning that 3 out of 4 nodes are removed, each network has only one node remaining, so the required number of driver nodes is also 1 for each network. The ordinal ranks are calculated and marked at each *p*_*N*_ value, where *μ*_*i*_^*X*^ represents the rank of network *X* (*X* = *A*, *B*, or *C*) at step *i* of an attack, *i* = 1,2, 3,4. After averaging over all 4 points, network A has an average rank *μ* = 1.5, network B has *μ* = 2.6, and network C has *μ* = 1.9. Here, *n*_*W*_ represents the number of winnings. Network A wins the first rank for 4 *p*_*N*_ values, network B wins only once when *p*_*N*_ = 0.75, and network C wins 3 times.

Since a smaller value of the density of the driver nodes *n*_*D*_ represents a better network controllability, the controllabilities of the simulated networks are ranked ordinally. Then, the overall average rank is taken, denoted by *μ*, which is the quantitative and comparative measure of the robustness of the network controllability. Here, the ordinal number is taken for comparison instead of the cardinal number, because at each step when a portion of nodes or edges have been removed from the network, one can focus more on the comparison to find which network requires a smaller number of driver nodes, while ignoring precisely how many drivers nodes are needed by each network. An example of such a quantitative measure is shown in Figures 3 and 4, where 3 networks are compared.

Three networks, denoted by A, B, and C, with 4 nodes and 4 edges, are shown in Figure 3. As the outdegree-based node-removal attack is performed, the remaining nodes and the required number of driver-nodes are shown in the figure. Initially, both networks A and C require only 1 driver-node, while network B requires 2. As can be seen from Figure 3(1), the maximum outdegree node in network B is node #1, while for networks A and C, every node has the same outdegree 1. Thus, the first attack is applied to node #1 in network B, while a random attack is performed on networks A and C. Since nodes are removed from network A, it always requires only 1 driver-node. For network B, the first attack breaks the network into two separated subnetworks. It should be noted that, in Figure 3C(2), it requires 2 control inputs to nodes #1 and #4. However, after node #4 is removed, it requires only 1 control input. Not only the needed number of driver-nodes *N*_*D*_ is reduced, but also the density *n*_*D*_ is reduced. This is also reflected by the simulation results shown in Figure 4, where the density curves show an upward trend, but it is not monotonically increasing. Finally, each network retains one and only one node, and *n*_*D*_ reaches 1.

In Figure 4, the corresponding density of driver-nodes, *n*_*D*_, as a function of the proportion of the removed nodes, *p*_*N*_, is plotted. Since a smaller value of the density *n*_*D*_ of the driver-nodes represents a better network controllability, at each point of *p*_*N*_, the controllabilities of the three networks are ranked ordinally, as marked in the figure. Then, the overall average rank is taken, denoted by *μ*, as a quantitative and comparative measure of the robustness of the network controllability. When two (or more) networks have the same value *n*_*D*_ at a sample point *p*_*N*_, they share the same rank. For example, when *p*_*N*_ = 0.5, for both networks A and C, *n*_*D*_ = 0.5; thus, they share the ranks 1 and 2, and the same average rank 1.5 is assigned to networks A and C, respectively.

It can be seen from Figure 4 that network A has better controllability robustness than networks B and C. However, given the many and complicated curves there, the comparison may not be clearly observed. For example, in the comparison of edge-removal attack, where the network size is 1000 and the average degree is 10, each curve has 10000 sample points to compare. Therefore, besides the average rank *μ*, the number *n*_*W*_ of winning the first place is also considered, which reflects the times that the network performs the best controllability under an attack. As shown in Figure 4, network A keeps requiring the minimum number of driver-nodes, among the three networks at each sample point, and thus the *n*_*w*_ value for network A is 4. A draw in the first place is also counted as a winning time. For network B, it only wins once, when *p*_*N*_ = 0.75, which is a draw.

The average rank *μ* and the number of winning times, *n*_*w*_, reflect two aspects of the comparison of network controllability robustness. A lower value of *mu* or a higher value of *n*_*w*_ is expected for a network model with good robustness of controllability.

This quantitative measure can be scaled to a setting with any number of networks and to edge-removal attacks as well.

### 3.3. QSNs with Different Redirecting Probabilities

Firstly, comparison is performed among the original QSN and the QSN with reversed edges. All networks have the same size with *N* = 1000 nodes and *M* = 6069 edges. Some other network settings are also studied, with results summarized in the SI file, where the network settings include *N* = 500 with average degree 〈*k*〉 = {5.38,10}, *N* = 1000 with 〈*k*〉 = {6.069,10,20}, and *N* = 2000  〈*k*〉 = {6.759,10,20} (*M* ≡ *N* × 〈*k*〉), where the average degree values 5.38, 6.069, and 6.759 are determined by the nature of MCN [25]. Note that (7) can be applied to sparse networks. When *N* = 500 and 〈*k*〉 = 20, *M*/*M*_*max*_ = 0.08 > 0.05; therefore, it is excluded from the experiments reported in SI.

Both state controllability and structural controllability are compared. Figures 6–11 show the curves of network controllability against the 6 different types of attacks. There are 11 networks being compared here, namely, the original QSN and the QSNs with *p*_*re*_ = 0.1 to 1.0, with an increment of 0.1. For clarity, only the results of the original QSN and the QSNs with *p*_*re*_ = 0.3, 0.5, 0.8, and 1.0 are presented in Figures 6–11.

The curves of heterogeneity and the numbers of three motifs are also plotted for reference. The heterogeneity of a network is calculated by *ξ* = 〈*k*^2^〉/〈*k*〉^2^, where 〈*k*〉 = ∑_*i*=1_^*N*^*k*_*i*_/*N* represents the average degree of the network, and 〈*k*^2^〉 = ∑_*i*=1_^*N*^*k*_*i*_^2^/*N* represents the raw second moment of the network [31].

**Figure 5:**
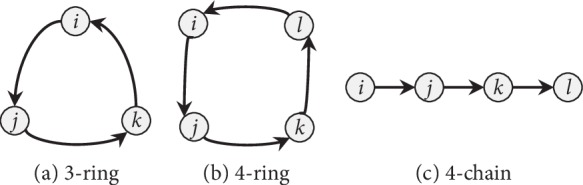
Topologies of the three motifs counted in simulations: (a) a 3-ring motif; (b) a 4-ring motif; and (c) a 4-chain motif.

The three counted motifs are shown in Figure 5. Empirically revealed, these three motifs are important to achieve robust network controllability [4, 30].

#### 3.3.1. Node-Removal Attacks

**Figure 6:**
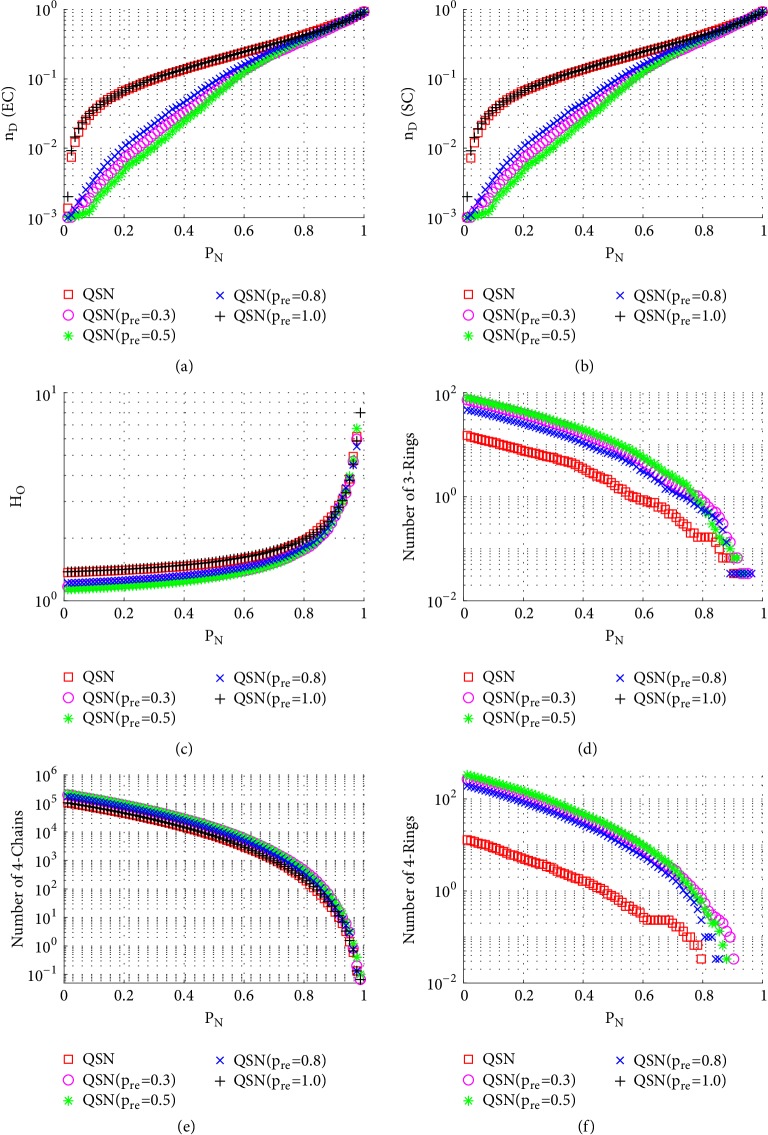
Simulation results of* random node-removal attack* on QSN variants: (a) exact controllability (EC); (b) structural controllability (SC); (c) heterogeneity of outdegree (*H*_*O*_); (d) number of 3-rings; (e) number of 4-chains; and (f) number of 4-rings. *P*_*N*_ represents the proportion of the removed nodes in the network.

Figure 6 shows the simulation results of random node-removal attacks. Comparing Figures 6(a), 6(b), 6(d), and 6(f), it can be seen that during a long period (0 < *P*_*N*_ < 0.8), QSN with *p*_*re*_ = 0.5 has the largest numbers of 3- and 4-rings and also has the best controllability robustness. Once the 3- and 4-rings are exhausted (around *P*_*N*_ > 0.9), the controllabilities of different networks become very similar to each other. The original QSN has fewer rings, while the QSN with *p*_*re*_ = 1.0 has no rings. All the networks have similar numbers of 4-chains, as shown in Figure 6(e), which does not distinguish the robustness of controllability in this case. The outdegree heterogeneity curves in Figure 6(c) show that the QSN with *p*_*re*_ = 0.5 has lowest *H*_*O*_ values and the original QSN and the QSN with *p*_*re*_ = 1.0 have the highest *H*_*O*_ values. Lower heterogeneity is beneficial against the random node-removal attack. When a higher-heterogeneity network is being attacked by random node removals, and when a large-degree node is removed, the controllability would decrease severely. However, in a lower-heterogeneity network, there are fewer large-degree nodes; therefore, the controllability could be maintained better than in a higher-heterogeneity one.

In summary, the QSN with *p*_*re*_ = 0.5 performs most robustly against random node-removal attacks, followed by those with *p*_*re*_ = 0.3 and *p*_*re*_ = 0.8. The original QSN and the QSN with *p*_*re*_ = 1.0 perform very similarly to each other, and they are ranked the last two. A positive correlation between the controllability robustness and the numbers of 3- and 4-rings is clearly observed. Many 3- and 4-rings in the network help maintain a good controllability.

**Figure 7:**
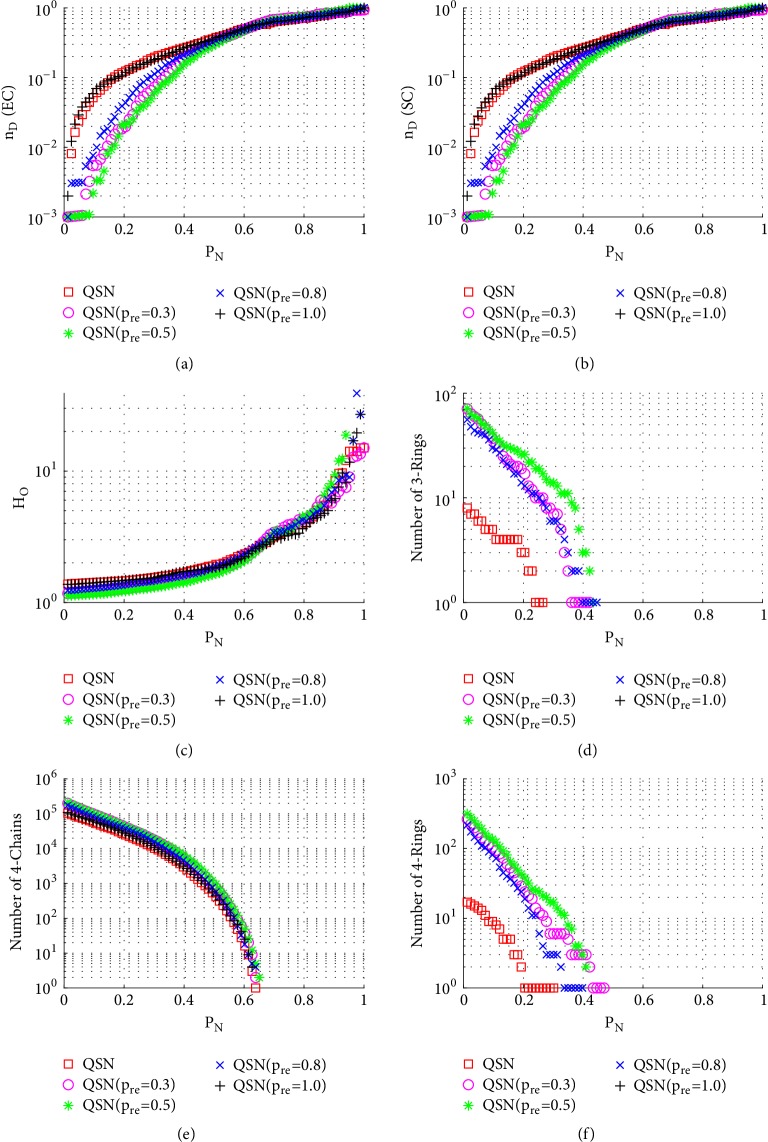
Simulation results of* betweenness-targeted node-removal attack* on QSN variants: (a) exact controllability (EC); (b) structural controllability (SC); (c) heterogeneity of outdegrees (*H*_*O*_); (d) number of 3-rings; (e) number of 4-chains; and (f) number of 4-rings. *P*_*N*_ represents the proportion of the removed nodes in the network.

Figure 7 shows the simulation results of betweenness-targeted node-removal attacks. This attack method breaks the 3-rings, 4-chains, and 4-rings much faster than random node-removal. For example, under this attack, the 3- and 4-rings are exhausted in all the networks before they reach *P*_*N*_ = 0.5, while under a random node-removal attack, the 3- and 4-rings are not exhausted even around *P*_*N*_ = 0.9. Except for this, the phenomenon seen from Figure 7 is similar to that from Figure 6. Redirected edges increase the numbers of 3- and 4-rings, as well as the robustness of controllability, of these networks.

**Figure 8:**
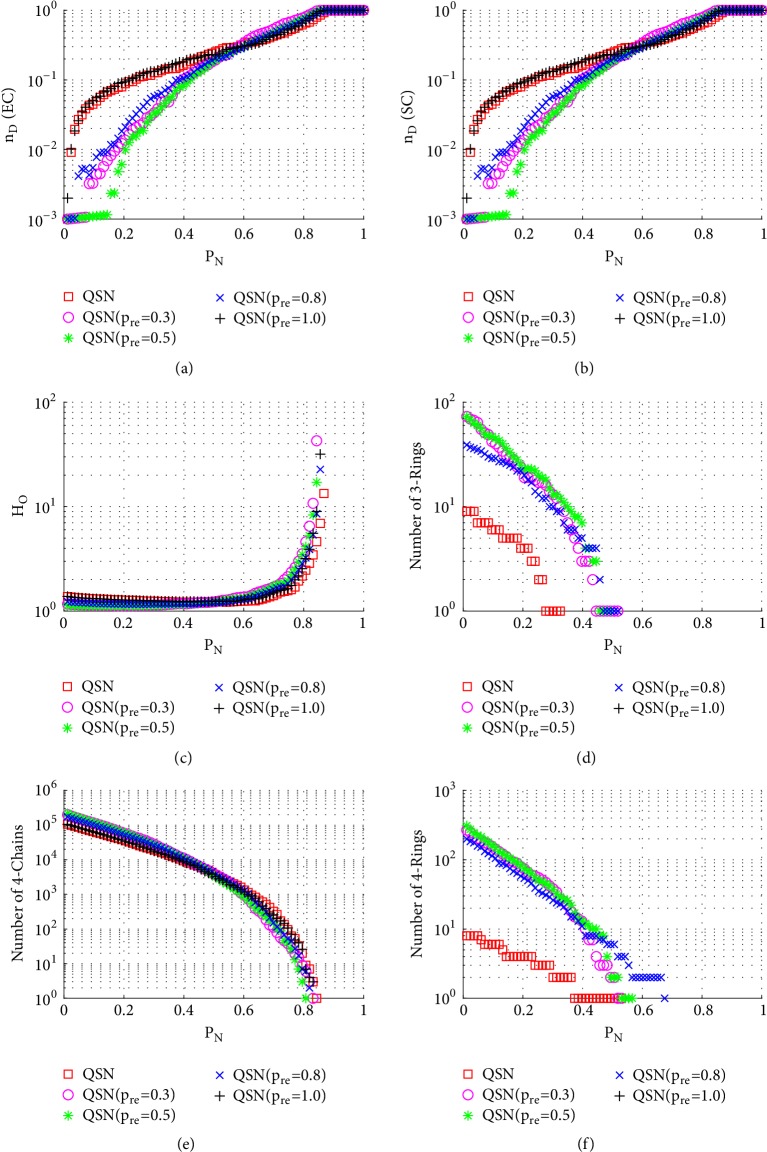
Simulation results of* degree-targeted node-removal attack* on QSN variants: (a) exact controllability (EC); (b) structural controllability (SC); (c) heterogeneity of outdegrees (*H*_*O*_); (d) number of 3-rings; (e) number of 4-chains; and (f) number of 4-rings. *P*_*N*_ represents the proportion of the removed nodes in the network.

Figure 8 shows the simulation results of degree-targeted node-removal attacks. A similar phenomenon, as seen in Figure 7, can be observed; namely, the QSN with *p*_*re*_ = 0.5 shows the best controllability robustness, which also has more motifs than other networks. Comparing Figures 7 and 8, (1) the betweenness-based node-removal attack is more harmful to the motifs, which are exhausted earlier under a betweenness-based attack than a degree-based attack; (2) after the 3- and 4-rings are exhausted, the controllabilities of different networks become similar to each other.

#### 3.3.2. Edge-Removal Attacks

**Figure 9:**
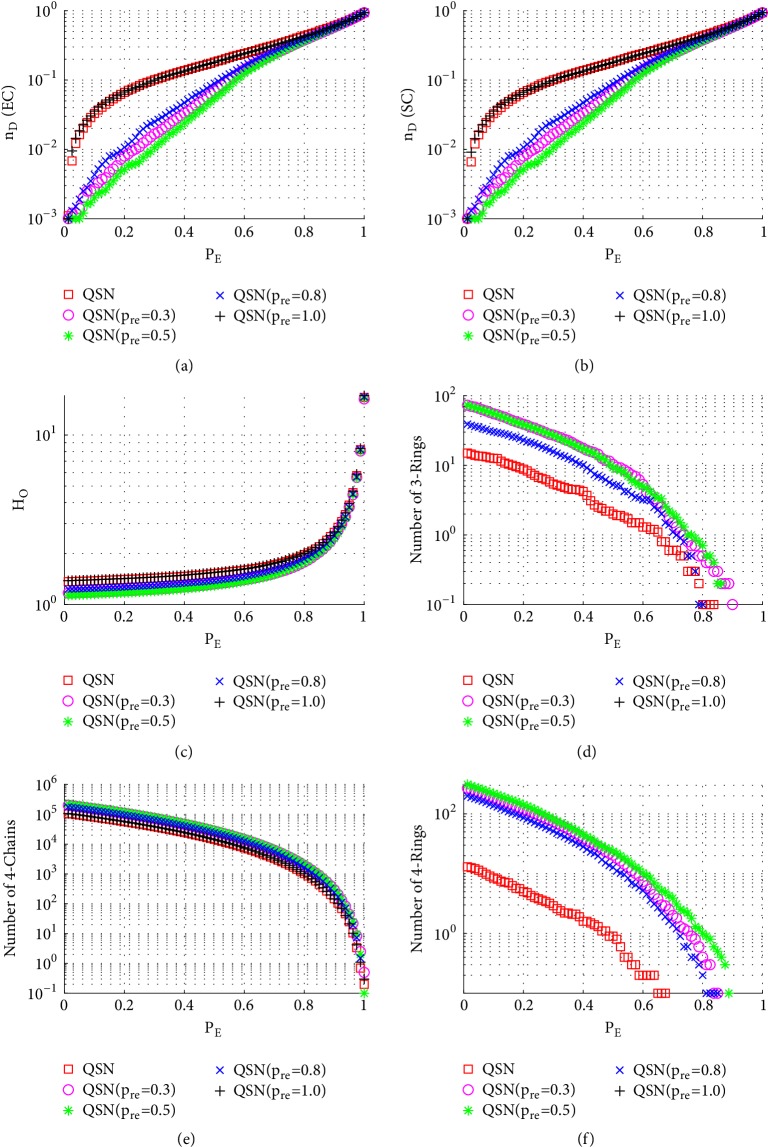
Simulation results of* random edge-removal attack* on QSN variants: (a) exact controllability (EC); (b) structural controllability (SC); (c) heterogeneity of outdegrees (*H*_*O*_); (d) number of 3-rings; (e) number of 4-chains; and (f) number of 4-rings. *P*_*E*_ represents the proportion of the removed edges in the network.

**Figure 10:**
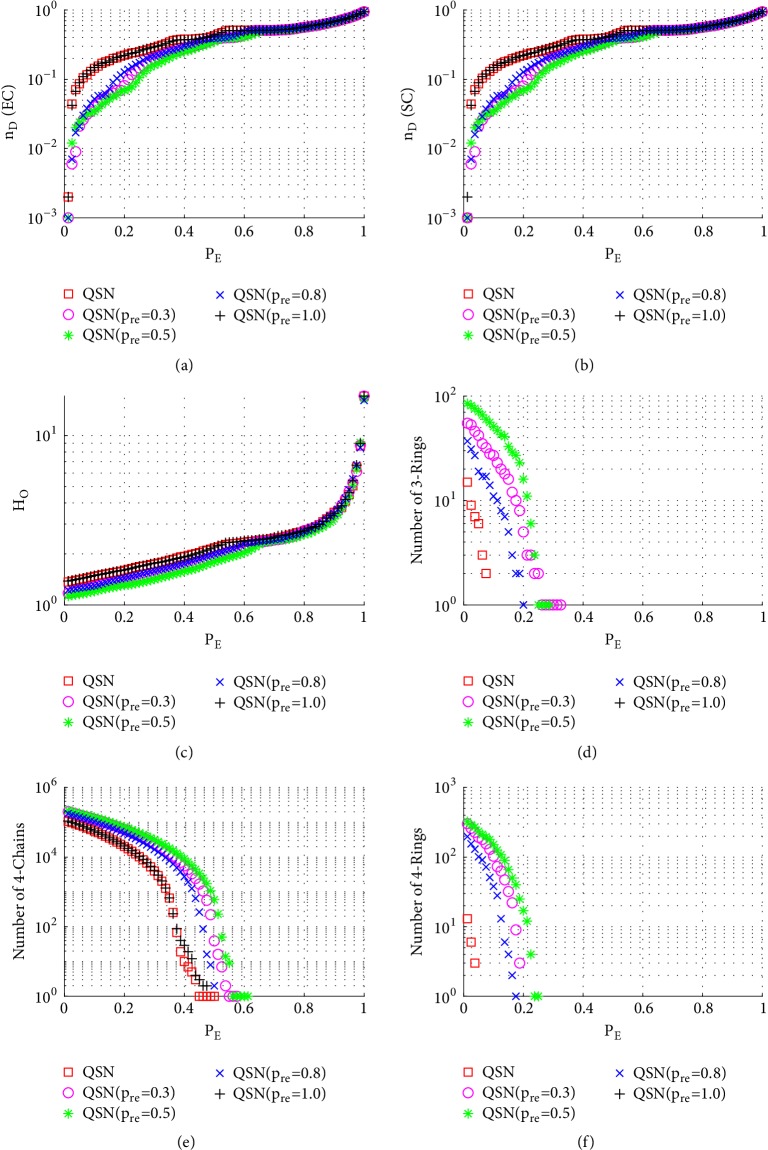
Simulation results of* betweenness-targeted edge-removal attack* on QSN variants: (a) exact controllability (EC); (b) structural controllability (SC); (c) heterogeneity of outdegrees (*H*_*O*_); (d) number of 3-rings; (e) number of 4-chains; and (f) number of 4-rings. *P*_*E*_ represents the proportion of the removed edges in the network.

**Figure 11:**
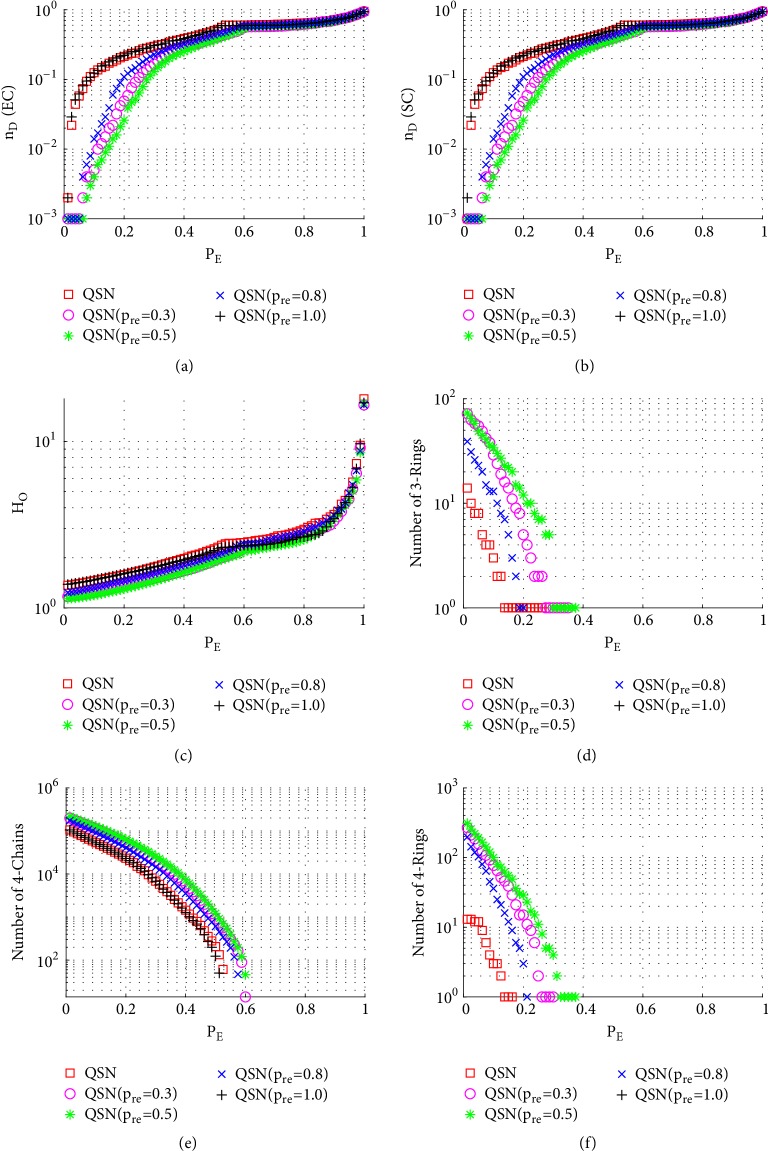
Simulation results of* degree-targeted edge-removal attack* on QSN variants: (a) exact controllability (EC); (b) structural controllability (SC); (c) heterogeneity of outdegrees (*H*_*O*_); (d) number of 3-rings; (e) number of 4-chains; and (f) number of 4-rings. *P*_*E*_ represents the proportion of the removed edges in the network.

Similarly to Figures 6, 7, and 8, here Figures 9, 10, and 11 show the simulation results of random, betweenness-targeted, and degree-targeted edge-removal attacks, respectively.

Similarly to the random node-removal, the random edge-removal is not effective on destroying motifs, while the two intentional edge-removal attacks are effective, where the 3- and 4-rings can be cleaned up before reaching *P*_*E*_ = 0.4. When the motifs are exhausted in a network, the required density of driver-nodes, *n*_*D*_, becomes high. Consistently, the QSN with *p*_*r*_*e* = 0.5 has the largest numbers of 3- and 4-rings during all the 6 attacks, and they maintain the best controllability robustness as well.

The full comparison results of the original QSN and the QSN with *p*_*re*_ = 0.1 to 1.0, with an increment of 0.1, are summarized in Tables 2 (for exact controllability) and 3 (for structural controllability). The average rank and the number of winning times are both listed. For example, in the first cell of Table 2, the number “9.95” means the average rank (R_N_) of the original QSN during the entire random node-removal attack process over all 11 networks. The number below inside parentheses represents the number of winning the first place during the entire process, where the “(1)” under “9.95” means that the QSN only wins the first rank once (perhaps a draw). A low value of average rank, or a high value of winning times, means better robustness of controllability.

**Figure 12:**
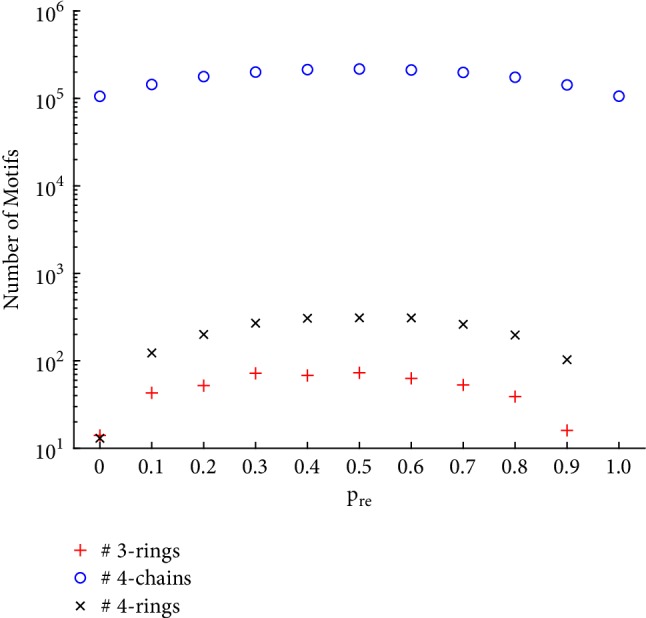
Comparison of the initial numbers of 3 motifs (3-rings, 4-rings, and 4-chains) in the QSN with different *p*_*re*_ values.

As shown in Tables 2 and 3, the minimum average ranks mostly appear around the QSN with *p*_*re*_ = 0.5, while the maximum ranks are bipolarly distributed in the original QSN and the QSN with *p*_*re*_ = 1.0. The controllability robustness becomes better as *p*_*re*_ increases from 0 (the original QSN could be considered as the QSN with *p*_*re*_ = 0) to 0.5 and then becomes worse as *p*_*re*_ changes from 0.5 to 1.0.  Figure 12 shows the initial number of 3 motifs in each network. When *p*_*re*_ = 0.5, the network has the largest numbers of 3-rings, 4-rings, and 4-chains.

### 3.4. Comparison with Other Network Models

The QSN with *p*_*re*_ = 0.5 is compared to 4 other complex network models, under the same 6 types of malicious attacks. The 4 models include random graph (RG) [1], multiplex congruence network (MCN) [25], random triangle network (RTN) [26, 32], and random rectangle network (RRN) [30]. These 4 models are compared to the original QSN in [30], where RRN shows overall best robustness of controllability. The uncomparable generic directed scale-free network is excluded in the comparison here. The generation methods of these networks are briefly introduced as follows.

For each model, possible isolated nodes, multiple edges, and self-loops, if existing in the final network, will be removed. Thus, a simple directed graph will be obtained as a result. For a fair comparison, the networks are generated with the same number of nodes *N* and the same number of edges *M*. Here, *N* = 1000 and *M* = 6069 (i.e., 〈*k*〉 = 6.069). An extensive comparison of the 5 networks is given in SI, where the parameters are set to *N* = 500 with 〈*k*〉 = {5.38,10}, *N* = 1000 with 〈*k*〉 = {6.069,10,20}, and *N* = 2000  〈*k*〉 = {6.759,10,20} with *M* ≡ *N* × 〈*k*〉.

#### 3.4.1. Random-Graph Networks

A directed RG network is generated as follows:

1. Start with *N* isolated nodes.
2. Pick up all possible pairs of nodes, denoted by *i* and *j* (*i* ≠ *j*, *i*, *j* = 1,2,…, *N*), once and once only, from the *N* nodes, and connect each pair of nodes by a directed edge with probability *p*_*RG*_ ∈ [0,1]. The edge is with an even probability 0.5 to be directed from *i* to *j* or from *j* to *i*.

Statistically, the expectation of the number of edges of an RG network is *E*(*M*) = *p*_*RG*_ · *N*(*N* − 1)/2. To precisely control the number of edges *M*, if necessary, uniformly at random adding or removing edges is performed where, when adding an edge, the direction can be random.

#### 3.4.2. Multiplex Congruence Networks

Each node in an MCN is marked by an index *i*, *i* = 1,2,…, *N*. The congruence relation of the node indices is applied to generate edges in the MCN. Given a congruence value *r*_*MCN*_, the MCN is generated as follows:

1. Start with *N* isolated nodes.
2. A directed edge *A*_*ij*_ is added if *j* ≡ *r*_*MCN*_(mod *i*).

This generates a layer (the *r*_*MCN*_th layer) in the multiplex. The degree distribution of each layer follows a power-law form. There is no pattern in the MCN, and its topology is determined by the two parameters *N* and *r*_*MCN*_.

#### 3.4.3. Random Triangle Networks

Based on the observations that a multiring structure benefits the robustness of controllability [4] and the network stability [33, 34] and that the triangular structure is frequently encountered in real-life situations, a directed RTN is designed here for comparison study of the network controllability robustness, as follows:

1. Start with *N* − 3 isolated nodes, with the other 3 nodes linked in as directed ring.
2. Randomly pick up two nodes, *i* and *j*, without edge *A*_*ij*_ or *A*_*ji*_ in between. Then, randomly pick up a node *k* from the neighbors of node *j*. If there is an edge *A*_*jk*_, then add two edges *A*_*ij*_ and *A*_*ki*_; otherwise, add two edges *A*_*ji*_ and *A*_*ik*_.
3. Repeat Step (2), until *M* edges have been added.

#### 3.4.4. Random Rectangle Networks

The above directed RTN is extended to an RRT, as follows:

1. Start with *N* − 4 isolated nodes, and the other 4 nodes are linked as a directed ring.
2. Randomly pick up three nodes, *i*, *j*, and *k*, without edges between any pair of them. Then, randomly pick up a node *w* from the neighbors of node *k*. If there is an edge *A*_*kw*_, then add edges *A*_*wi*_, *A*_*ij*_, and *A*_*jk*_; otherwise (with an edge *A*_*wk*_), add edges *A*_*ki*_, *A*_*ij*_, and *A*_*jw*_.
3. Repeat Step (2), until *M* edges have been added.

Since, at each time step, two edges are added to RTN, and three edges are added to RRN; uniform at random adding or removing edges can be performed to control the number of edges precisely.

#### 3.4.5. Simulation Results

**Figure 13:**
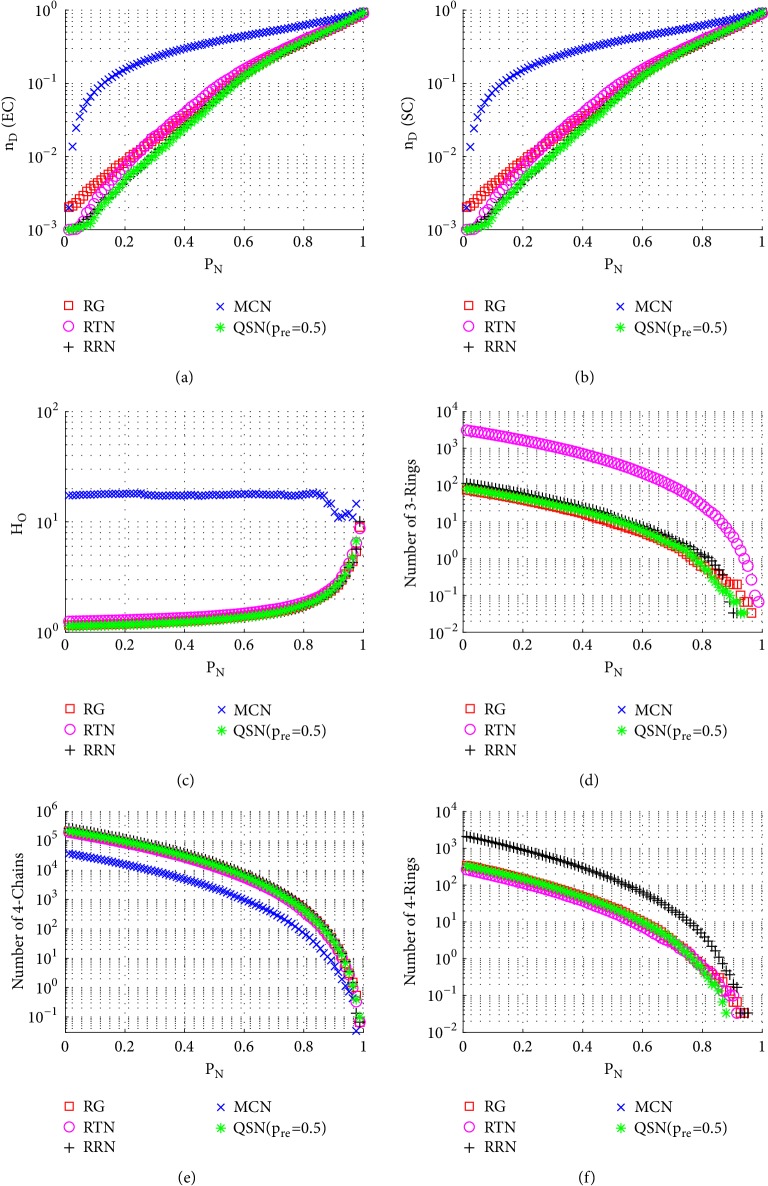
Simulation results of* random node-removal attack* on the 5 networks: (a) exact controllability (EC); (b) structural controllability (SC); (c) heterogeneity of outdegrees (*H*_*O*_); (d) number of 3-rings; (e) number of 4-chains; and (f) number of 4-rings. *P*_*N*_ represents the proportion of the removed nodes in the network.

**Figure 14:**
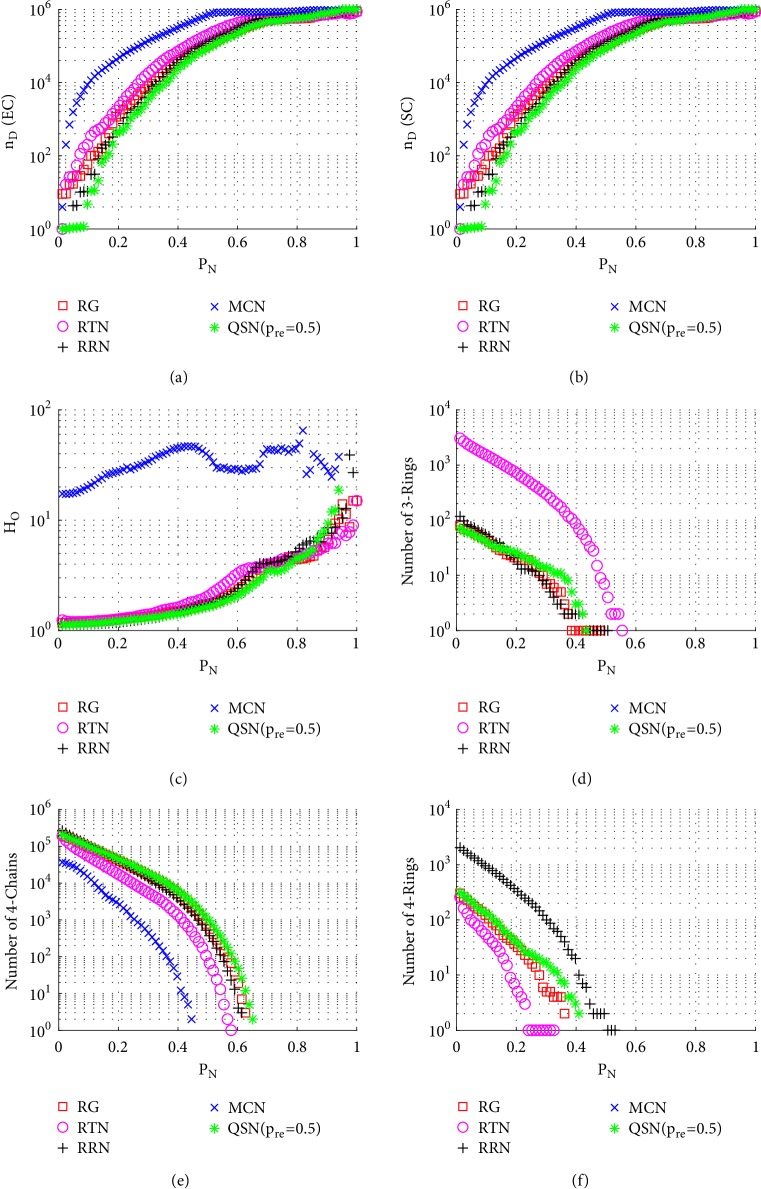
Simulation results of* betweenness-targeted node-removal attack* on the 5 networks: (a) exact controllability (EC); (b) structural controllability (SC); (c) heterogeneity of outdegrees (*H*_*O*_); (d) number of 3-rings; (e) number of 4-chains; and (f) number of 4-rings. *P*_*N*_ represents the proportion of the removed nodes in the network.

**Figure 15:**
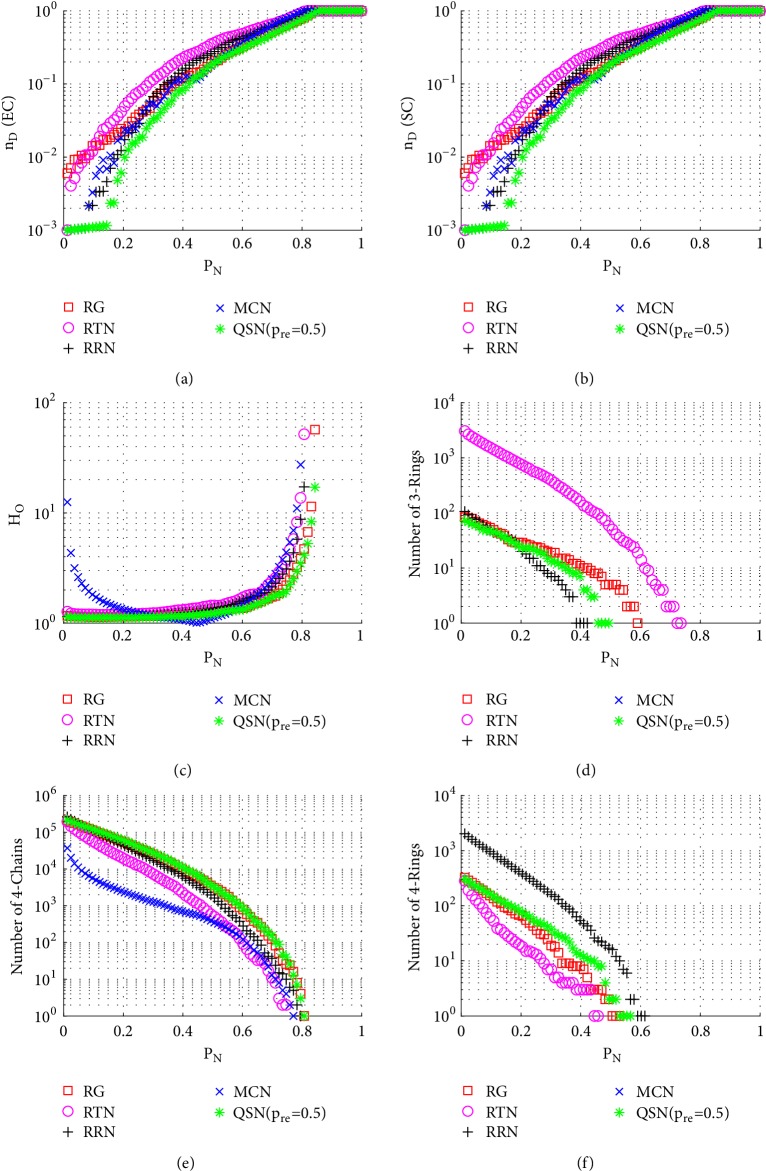
Simulation results of* degree-targeted node-removal attack* on the 5 networks: (a) exact controllability (EC); (b) structural controllability (SC); (c) heterogeneity of outdegrees (*H*_*O*_); (d) number of 3-rings; (e) number of 4-chains; and (f) number of 4-rings. *P*_*N*_ represents the proportion of the removed nodes in the network.

**Figure 16:**
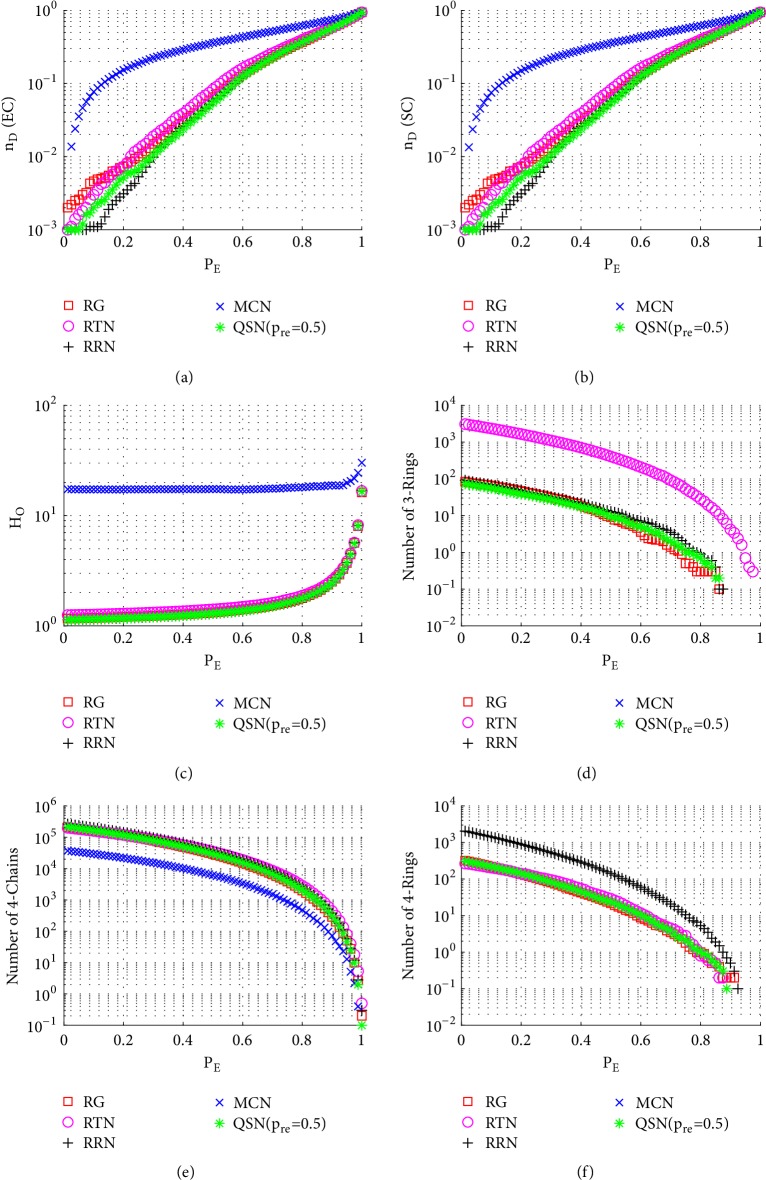
Simulation results of* random edge-removal attack* on the 5 networks: (a) exact controllability (EC); (b) structural controllability (SC); (c) heterogeneity of outdegrees (*H*_*O*_); (d) number of 3-rings; (e) number of 4-chains; and (f) number of 4-rings. *P*_*E*_ represents the proportion of the removed edges in the network.

**Figure 17:**
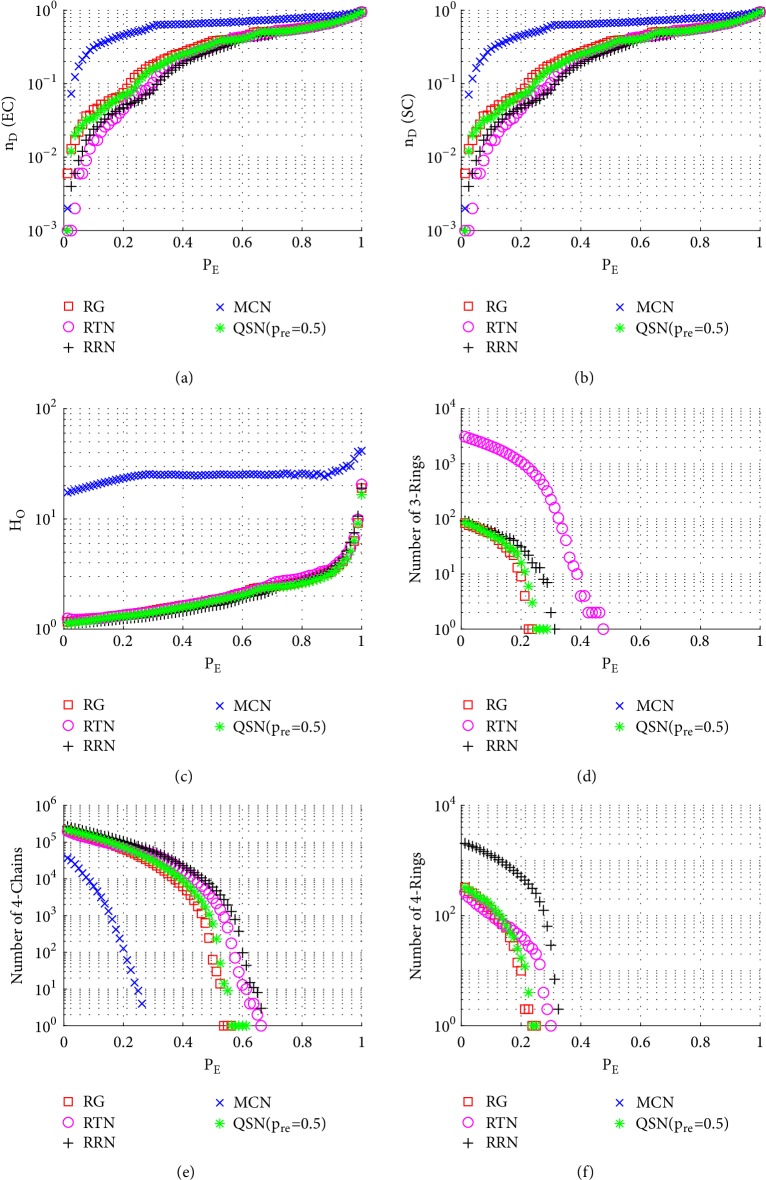
Simulation results of* betweenness-targeted edge-removal attack* on the 5 networks: (a) exact controllability (EC); (b) structural controllability (SC); (c) heterogeneity of outdegrees (*H*_*O*_); (d) number of 3-rings; (e) number of 4-chains; and (f) number of 4-rings. *P*_*E*_ represents the proportion of the removed edges in the network.

**Figure 18:**
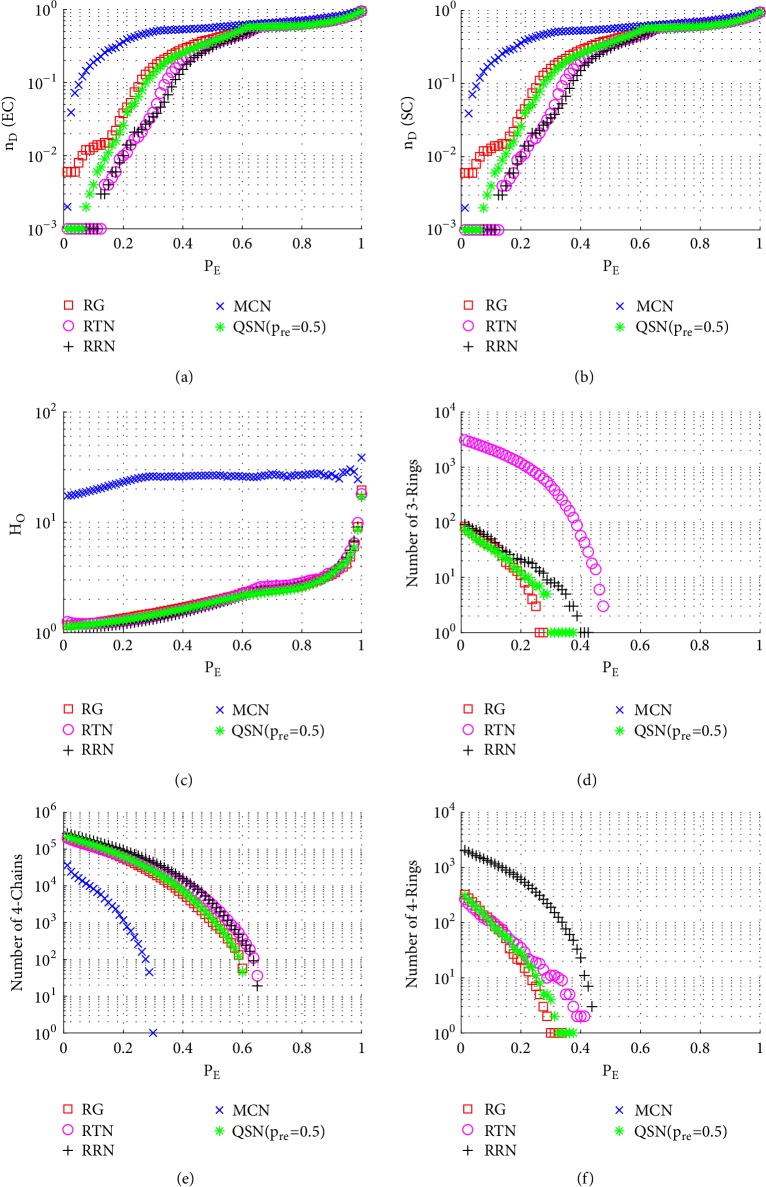
Simulation results of* degree-targeted edge-removal attack* on the 5 networks: (a) exact controllability (EC); (b) structural controllability (SC); (c) heterogeneity of outdegrees (*H*_*O*_); (d) number of 3-rings; (e) number of 4-chains; and (f) number of 4-rings. *P*_*E*_ represents the proportion of the removed edges in the network.

Figures 13–18 show a comparison of the 5 networks. From these figures, some common features can be observed: (1) RTN has the largest number of 3-rings, since it is based on triangles; (2) RRN has the largest number of 4-rings, since it is based on rectangles; and (3) MCN has the largest value of *H*_*O*_, which is the only topology with power-law degree distribution in this comparison.

As can be seen from Figures 13, 14, and 15, QSN with *p*_*r*_*e* = 0.5 clearly outperforms the other 4 topologies against the node-removal attacks.

It can be seen from Figures 16, 17, and 18 that the QSN with *p*_*r*_*e* = 0.5 clearly outperforms the other 4 topologies against node-removal attacks.

Figure 16 shows the simulation results of the 5 networks against random node-removal attacks. The QSN with *p*_*r*_*e* = 0.5 clearly outperforms RG, RTN, and MCN and has similar performance comparable to RRN. RRN has better controllability when *P*_*E*_ is near but less than 0.4; after that, the performances are undistinguishable. Quantitatively, according to Tables 4 and 5, the QSN with *p*_*r*_*e* = 0.5 outperforms RRN in both the average rank and the number of winning times.

In Figures 17 and 18, the QSN with *p*_*r*_*e* = 0.5 is clearly ranked at the third place among the 5 network topologies. Both RRN and RTN perform better than the QSN with *p*_*r*_*e* = 0.5, under both betweenness-targeted and degree-targeted edge-removal attacks.

Tables 4 and 5 show the average ranks and the number of winning times in the comparison among the 5 networks. Overall, the QSN with *p*_*r*_*e* = 0.5 gains the lowest average rank. RRN has the maximum number of winning times, due to its strong performance against the betweenness-targeted and degree-targeted edge-removal attacks.

### 3.5. Effects of Degree Distributions

Finally, the effect of a degree distribution on the robustness of network controllability is briefly discussed.

**Figure 19:**
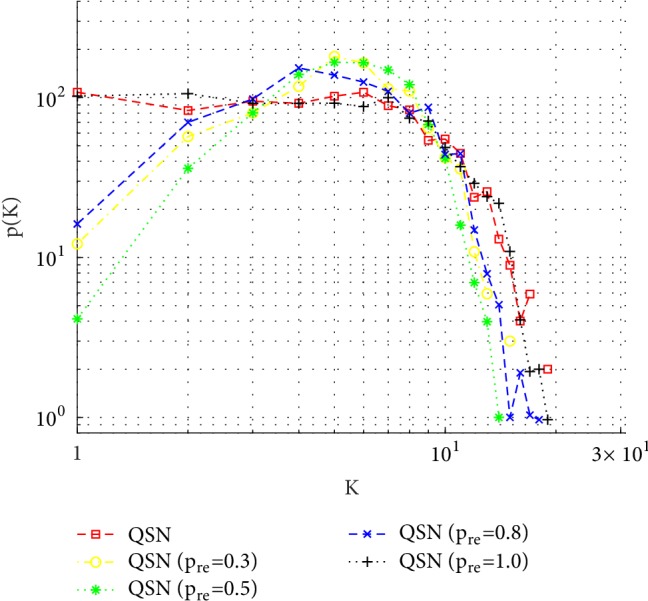
Degree distributions of the QSN with different *p*_*re*_ values. The original QSN and the QSN with *p*_*re*_ = 0.1 have a uniform outdegree distribution, while the QSN with *p*_*re*_ = 0.3, 0.5, and 0.8 has a Poisson-like outdegree distribution.

Figure 19 shows the degree distributions of the QSN variants. Together with these distributions, the relationships between the numbers of motifs (mainly, 3- and 4-rings and 4-chains) and the controllability robustness are observed, as summarized below:

1. If the degree distribution of the QSN (or a variant) network is not uniform but Poisson, then the numbers of 3- and 4-rings are positively correlated to the controllability robustness: the more rings, the better controllability robustness.
2. If the degree distribution of the QSN (or a variant) network is uniform (only for the original QSN and the QSN with *p*_*re*_ = 1.0), the existence of many rings in the original QSN and the absence of rings in the QSN with *p*_*re*_ = 1.0 do not affect the controllability robustness.

The extensive comparison results presented in SI confirms that the existence of many 3- and 4-rings in the QSN with *p*_*re*_ = 0.5 improves the robustness of the controllability, when both network size and average degree are changed.

## 4. Conclusions

Inspired by the idea of small-world network model, which was formed by randomly* rewiring* edges, aiming to enhance the synchronizability of an undirected nearest-neighbor regular network, this paper proposed a new network model by randomly* redirecting* edges, aiming to enhance the controllability robustness of a directed snapback network against both random and intentional node-removal and edge-removal attacks. Through extensive numerical simulations, it was found that the modified* q*-snapback network, with statistically one-half of the feedforward edges being redirected, is overall better than other variants, and also better than comparable random networks, multiplex congruence networks, random triangle networks, and random rectangle networks, against various attacks. It was found that there are many 3-rings and 4-rings in the modified* q*-snapback networks, which seems to be the main reason that make such networks strong in resisting malicious attacks. The advantageous role of ring motifs in complex networks remains to be further understood in future studies towards more and better technical network applications.

## Figures and Tables

**Table 1:** Attack methods in simulations.

		Node-removal	Edge-removal
Intentional	Betweenness	TB_N_: Remove the node with the largest betweenness	TB_E_: Remove the edge with the largest betweenness
	Degree	TD_N_: Remove the node with the largest outdegree	TD_E_: Remove the edge with the largest edge degree

Random		R_N_: Remove a node randomly	R_E_: Remove an edge randomly

**Table 2:** Comparison of the original QSN and the QSN with redirected edges in terms of exact controllability. Average rank and number of winning rank one are listed for each network. Italic numbers represent the minimum average rank, and italic numbers inside parentheses mean the maximum number of winning times.

EC	R_N_	TB_N_	TD_N_	R_E_	TB_E_	TD_E_	Average
QSN	9.95	7.88	7.35	10.57	9.33	10.39	9.25
	(1)	(87)	(321)	(10)	(18)	(24)	(77)

QSN *p*_*re*_ = 0.1	8.38	7.82	6.69	8.36	7.63	8.25	7.85
	(4)	(20)	(149)	(41)	(47)	(185)	(74)

QSN *p*_*re*_ = 0.2	6.20	5.11	7.15	6.45	5.83	5.42	6.03
	(16)	(81)	(146)	(111)	(257)	(174)	(131)

QSN *p*_*re*_ = 0.3	3.89	5.82	6.25	4.58	4.46	4.10	4.85
	(154)	(71)	(221)	(111)	(199)	(1062)	(303)

QSN *p*_*re*_ = 0.4	3.19	*4.00 *	4.37	2.23	*2.09 *	2.97	3.14
	(107)	*(417) *	(390)	(1410)	(2115)	(1311)	(958)

QSN *p*_*re*_ = 0.5	2.78	4.91	*4.08 *	*1.69 *	2.14	*1.55 *	*2.86 *
	(329)	(354)	*(432) *	*(4218) *	*(3532) *	*(4607) *	*(2245) *

QSN *p*_*re*_ = 0.6	*2.28 *	4.71	4.09	2.44	3.25	3.93	3.45
	*(401) *	(112)	(394)	(854)	(434)	(416)	(435)

QSN *p*_*re*_ = 0.7	4.71	4.66	5.73	4.33	5.70	5.18	5.05
	(32)	(147)	(163)	(288)	(297)	(109)	(173)

QSN *p*_*re*_ = 0.8	6.68	6.33	5.64	6.62	7.46	6.82	6.59
	(11)	(32)	(162)	(46)	(67)	(263)	(97)

QSN *p*_*re*_ = 0.9	7.76	6.69	6.99	8.42	8.69	8.30	7.81
	(63)	(75)	(144)	(30)	(104)	(119)	(89)

QSN *p*_*re*_ = 1.0	10.18	8.08	7.67	10.31	9.40	9.09	9.12
	(14)	(76)	(230)	(11)	(20)	(104)	(76)

**Table 3:** Comparison of the original QSN and the QSN with redirected edges in terms of structural controllability. Average rank and number of winning rank one are listed for each network. Italic numbers represent the minimum average rank, and italic numbers inside parentheses mean the maximum number of winning times.

SC	R_N_	TB_N_	TD_N_	R_E_	TB_E_	TD_E_	Average
QSN	9.95	7.88	7.35	10.58	9.33	10.39	9.25
	(2)	(87)	(321)	(14)	(19)	(25)	(78)

QSN *p*_*re*_ = 0.1	8.38	7.82	6.67	8.36	7.64	8.25	7.85
	(4)	(20)	(149)	(41)	(47)	(185)	(74)

QSN *p*_*re*_ = 0.2	6.21	5.13	7.14	6.45	5.84	5.42	6.03
	(16)	(81)	(146)	(111)	(257)	(174)	(131)

QSN *p*_*re*_ = 0.3	3.88	5.83	6.25	4.58	4.46	4.10	4.85
	(159)	(71)	(221)	(111)	(199)	(1062)	(304)

QSN *p*_*re*_ = 0.4	3.21	*4.02 *	4.38	2.22	*2.09 *	2.98	3.15
	(106)	*(393) *	(382)	(1410)	(2109)	(1257)	(943)

QSN *p*_*re*_ = 0.5	2.75	4.88	4.09	*1.69 *	2.15	*1.54 *	*2.85 *
	(347)	(372)	*(432) *	*(4219) *	*(3543) *	*(4646) *	*(2260) *

QSN *p*_*re*_ = 0.6	*2.30 *	4.71	*4.04 *	2.44	3.26	3.93	3.45
	*(385) *	(111)	(400)	(853)	(443)	(416)	(435)

QSN *p*_*re*_ = 0.7	4.71	4.65	5.76	4.33	5.72	5.18	5.06
	(31)	(147)	(163)	(288)	(297)	(109)	(173)

QSN *p*_*re*_ = 0.8	6.67	6.32	5.65	6.62	7.40	6.82	6.58
	(11)	(32)	(161)	(46)	(67)	(263)	(97)

QSN *p*_*re*_ = 0.9	7.76	6.69	7.00	8.42	8.70	8.30	7.81
	(67)	(75)	(144)	(30)	(104)	(119)	(90)

QSN *p*_*re*_ = 1.0	10.18	8.07	7.67	10.29	9.41	9.09	9.12
	(14)	(76)	(230)	(11)	(20)	(104)	(76)

**Table 4:** Comparison of robustness of exact controllability among 5 networks. Average rank and number of winning rank one are listed for each network. Italic numbers represent the minimum average rank, and italic numbers inside parentheses mean the maximum number of winning times.

EC	R_N_	TB_N_	TD_N_	R_E_	TB_E_	TD_E_	Average
RG	3.12	2.28	2.94	3.08	3.64	3.66	3.12
	(29)	(173)	(167)	(143)	(45)	(4)	(94)

RTN	3.62	3.41	4.45	3.80	2.33	2.54	3.36
	(51)	(107)	(166)	(65)	(1244)	(1474)	(518)

RRN	*1.61 *	2.85	3.19	1.63	*1.65 *	*1.32 *	2.04
	(455)	(60)	(223)	(2902)	*(3272) *	*(4908) *	*(1970) *

MCN	4.99	4.94	2.96	4.99	5.00	5.00	4.65
	(2)	(12)	(275)	(9)	(6)	(4)	(51)

QSN *p*_*re*_ = 0.5	1.66	*1.53 *	*1.45 *	*1.49 *	2.38	2.48	*1.83 *
	*(520) *	*(784) *	*(933) *	*(3393) *	(1749)	(1197)	(1429)

**Table 5:** Comparison of robustness of structural controllability among 5 networks. Average rank and number of winning rank one are listed for each network. Italic numbers represent the minimum average rank, and italic numbers inside parentheses mean the maximum number of winning times.

SC	R_N_	TB_N_	TD_N_	R_E_	TB_E_	TD_E_	Average
RG	3.12	2.29	2.93	3.08	3.65	3.66	3.12
	(29)	(173)	(171)	(140)	(33)	(4)	(92)

RTN	3.62	3.41	4.45	3.80	2.33	2.54	3.36
	(51)	(107)	(166)	(65)	(1244)	(1474)	(518)

RRN	*1.60 *	2.84	3.19	1.63	*1.65 *	*1.32 *	2.04
	(462)	(60)	(223)	(2958)	*(3272) *	*(4908) *	*(1981) *

MCN	4.99	4.94	2.97	4.99	5.00	5.00	4.65
	(2)	(12)	(275)	(9)	(6)	(4)	(51)

QSN *p*_*re*_ = 0.5	1.67	*1.53 *	*1.45 *	*1.50 *	2.38	2.48	*1.83 *
	*(508) *	*(784) *	*(932) *	*(3342) *	(1749)	(1197)	(1419)

## Supplementary Materials

* *Supplementary Materials Supplemental information includes 32 tables and 97 figures can be found with this article online.
