# Supplementary material for: Enhancing Controllability Robustness of q-Snapback Networks through Redirecting Edges
Source: Research (Wash D C). 2019 Aug 4;2019:7857534. doi: 10.34133/2019/7857534 (PMC7006948; doi:10.34133/2019/7857534)

Research
OFFICIAL JOURNAL OF CAST

Supplementary Material for the Paper
"Enhancing Controllability Robustness of *q*-Snapback Networks through Redirecting Edges"

Yang Lou[1], LinWang[2], and Guanrong Chen[1]

[1]*City University of Hong Kong, Hong Kong*
[2]*Shanghai Jiao Tong University, Shanghai 200240, China*

Correspondence should be addressed to Guanrong Chen; eegchen@cityu.edu.hk

1 Expected Outdegrees and Indegrees

In Figure S1, the expected outdegree and indegree for each node calculated according to Eqs. (1) and (2) are plotted together with the real degrees of each node averaged from 1000 independent runs. The network size is N=1000. It can be seen from Figure S1 that the expected outdegree and indegree of each node can be precisely calculated by Eqs. (1) and (2), respectively.

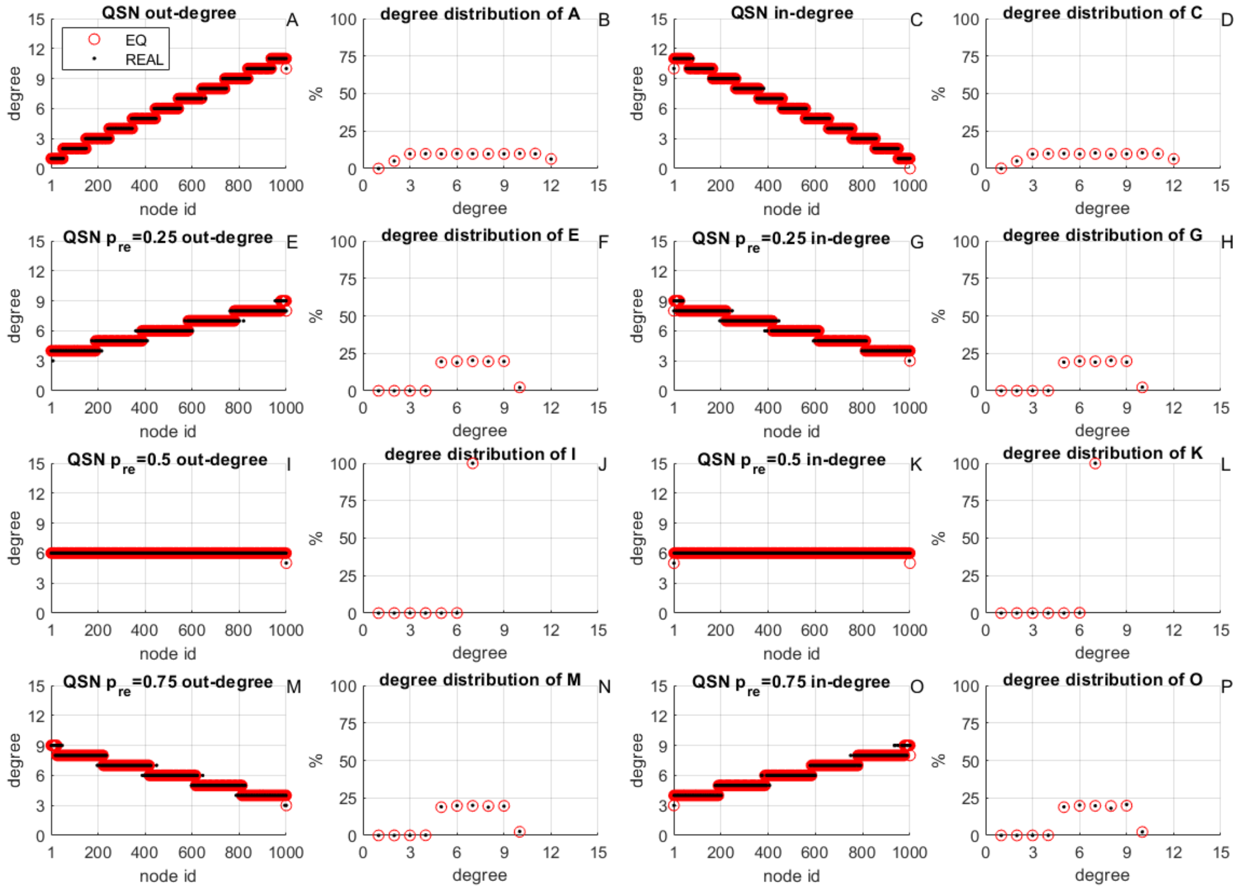

Figure S1 (A, C, E, G, I, K, M, and O) The expected outdegree and indegree for node id (1,2, ..., 1000) is plotted in red circle, and the real degree (averaged from 1000 independent runs) is plotted in black dot. (B, D, F, H, J, L, N, and P) The according degree distribution of its left figure.

2 QSN vs. QSN Variants Comparison Tables

The comparisons of the original q-snapback network (QSN) and the QSN variants (with redirected edges) are given in Tables. S1 to S16. The network size is set to N=500, N=1000, and N=2000, respectively. For N=500, the average degree of networks is set to $\langle k \rangle$=5.38 and $\langle k \rangle$=10, respectively. For N=1000, the average degree is set to $\langle k \rangle$={6.069, 10, 20}, respectively. For N=2000, the average degree is set to $\langle k \rangle$={6.759, 10, 20}, respectively. Both exact controllability (EC) and structural controllability (SC) are compared. Note that when N=500 and $\langle k \rangle$=20, then the ratio between the number of edges M and possible maximum number of edges M_{max} is M/M_{max}=0.08>0.05, meaning it is not a sparse network and the calculation of exact controllability cannot be applied to it, and thus excluded from the comparison.

In each cell of the table, the real number represents the average rank, and the integer inside the parentheses mean the number of winning times. Italic real numbers (with gray-shaded) represent the minimum average rank, and italic numbers inside parentheses (with gray-shaded) mean the maximum average number of winning times.

2.1 Network Size N=500

Table S1. [N=500, $\langle k \rangle$=5.38] Comparison of the original QSN and the QSN with redirected edges in terms of exact controllability (EC).

EC	R_N	TB_N	TD_N	R_E	TB_E	TD_E	Average
QSN	10.49<br>(2)	7.33<br>(66)	8.06<br>(74)	10.11<br>(4)	8.75<br>(88)	7.39<br>(425)	8.69<br>(110)
QSN<br>p_{re}=0.1	8.37<br>(2)	7.34<br>(21)	5.48<br>(180)	8.12<br>(9)	8.34<br>(90)	8.51<br>(93)	7.69<br>(66)
QSN<br>p_{re}=0.2	6.04<br>(9)	6.82<br>(29)	6.73<br>(75)	6.34<br>(65)	7.37<br>(19)	6.36<br>(76)	6.61<br>(46)
QSN<br>p_{re}=0.3	3.96<br>(13)	4.41<br>(172)	4.14<br>(142)	4.17<br>(168)	3.31<br>(406)	5.99<br>(128)	4.33<br>(172)
QSN<br>p_{re}=0.4	1.95<br>(193)	3.81<br>(138)	3.61<br>(244)	2.97<br>(278)	4.34<br>(233)	2.40<br>(743)	*3.18*<br>(305)
QSN<br>p_{re}=0.5	2.03<br>(238)	4.95<br>(27)	6.42<br>(167)	1.39<br>(2148)	3.01<br>(1384)	2.66<br>(1056)	3.41<br>*(837)*
QSN<br>p_{re}=0.6	3.70<br>(76)	4.99<br>(101)	4.33<br>(140)	2.77<br>(396)	4.24<br>(609)	2.91<br>(974)	3.82<br>(383)
QSN<br>p_{re}=0.7	5.13<br>(6)	6.06<br>(30)	6.38<br>(90)	4.56<br>(8)	4.15<br>(369)	4.64<br>(89)	5.15<br>(99)
QSN<br>p_{re}=0.8	5.85<br>(12)	7.22<br>(56)	7.39<br>(89)	6.65<br>(22)	6.31<br>(90)	6.45<br>(30)	6.65<br>(50)
QSN<br>p_{re}=0.9	8.72<br>(2)	4.97<br>(139)	5.37<br>(144)	8.27<br>(30)	7.22<br>(92)	9.28<br>(54)	7.30<br>(77)
QSN<br>p_{re}=1.0	9.75<br>(1)	8.10<br>(29)	8.09<br>(92)	10.65<br>(13)	8.98<br>(19)	9.40<br>(44)	9.16<br>(33)

Table S2. [N=500, $\langle k \rangle$=5.38] Comparison of the original QSN and the QSN with redirected edges in terms of structural controllability (SC).

**SC**	**R_N**	**TB_N**	**TD_N**	**R_E**	**TB_E**	**TD_E**	**Average**
QSN	10.50<br>(2)	7.35<br>(66)	8.06<br>(74)	10.12<br>(4)	8.81<br>(88)	7.40<br>(425)	8.71<br>(110)
QSN<br>p_{re}=0.1	8.37<br>(2)	7.34<br>(21)	5.49<br>(179)	8.11<br>(9)	8.25<br>(90)	8.52<br>(93)	7.68<br>(66)
QSN<br>p_{re}=0.2	6.05<br>(9)	6.83<br>(29)	6.73<br>(75)	6.33<br>(65)	7.41<br>(21)	6.35<br>(76)	6.62<br>(46)
QSN<br>p_{re}=0.3	3.95<br>(13)	4.42<br>(172)	4.14<br>(142)	4.15<br>(168)	3.32<br>(382)	6.00<br>(128)	4.33<br>(168)
QSN<br>p_{re}=0.4	1.93<br>(192)	3.83<br>(137)	3.62<br>(236)	2.99<br>(278)	4.27<br>(253)	2.41<br>(714)	*3.17*<br>(302)
QSN<br>p_{re}=0.5	1.98<br>(247)	4.94<br>(35)	6.43<br>(167)	1.39<br>(2153)	3.02<br>(1363)	2.68<br>(1037)	3.41<br>*(834)*
QSN<br>p_{re}=0.6	3.78<br>(67)	5.00<br>(100)	4.33<br>(142)	2.76<br>(389)	4.27<br>(611)	2.90<br>(989)	3.84<br>(383)
QSN<br>p_{re}=0.7	5.15<br>(6)	6.06<br>(30)	6.39<br>(89)	4.58<br>(8)	4.16<br>(359)	4.63<br>(89)	5.16<br>(97)
QSN<br>p_{re}=0.8	5.82<br>(12)	7.21<br>(56)	7.31<br>(89)	6.66<br>(22)	6.31<br>(90)	6.44<br>(30)	6.62<br>(50)
QSN<br>p_{re}=0.9	8.72<br>(2)	4.98<br>(139)	5.42<br>(144)	8.27<br>(30)	7.22<br>(92)	9.27<br>(54)	7.32<br>(77)
QSN<br>p_{re}=1.0	9.74<br>(1)	8.04<br>(37)	8.09<br>(92)	10.64<br>(13)	8.96<br>(19)	9.41<br>(44)	9.15<br>(34)

Table S3. [N=500, $\langle k \rangle$=10] Comparison of the original QSN and the QSN with redirected edges in terms of exact controllability (EC).

**EC**	**R_N**	**TB_N**	**TD_N**	**R_E**	**TB_E**	**TD_E**	**Average**
QSN	10.50<br>(3)	9.33<br>(11)	8.33<br>(60)	10.65<br>(21)	8.58<br>(49)	10.59<br>(17)	9.66<br>(27)
QSN<br>p_{re}=0.1	7.81<br>(50)	6.97<br>(59)	6.34<br>(150)	8.53<br>(200)	8.59<br>(392)	8.40<br>(393)	7.77<br>(207)
QSN<br>p_{re}=0.2	6.26<br>(40)	5.66<br>(70)	3.94<br>(289)	6.71<br>(449)	6.28<br>(195)	5.11<br>(848)	5.66<br>(315)
QSN<br>p_{re}=0.3	4.57<br>(81)	4.82<br>(90)	7.00<br>(132)	4.03<br>(1438)	3.49<br>(756)	5.00<br>(829)	4.82<br>(554)
QSN<br>p_{re}=0.4	3.23<br>(137)	4.41<br>(219)	4.91<br>(273)	2.86<br>(1990)	5.73<br>(181)	2.88<br>(1487)	4.00<br>(715)
QSN<br>p_{re}=0.5	1.83<br>(437)	4.29<br>(236)	5.13<br>(225)	2.04<br>(3598)	2.53<br>(2929)	2.59<br>(2840)	*3.07*<br>*(1711)*
QSN<br>p_{re}=0.6	3.10<br>(184)	4.53<br>(141)	4.07<br>(273)	2.41<br>(2415)	2.94<br>(697)	2.38<br>(2647)	3.24<br>(1060)
QSN<br>p_{re}=0.7	4.21<br>(114)	4.06<br>(149)	5.61<br>(167)	4.34<br>(1157)	5.20<br>(714)	4.65<br>(996)	4.68<br>(550)
QSN<br>p_{re}=0.8	6.23<br>(39)	6.59<br>(27)	5.09<br>(168)	5.95<br>(507)	4.99<br>(439)	6.52<br>(579)	5.90<br>(293)
QSN<br>p_{re}=0.9	8.31<br>(14)	6.74<br>(64)	6.79<br>(61)	8.32<br>(36)	8.22<br>(65)	8.24<br>(425)	7.77<br>(111)
QSN<br>p_{re}=1.0	9.96<br>(1)	8.61<br>(23)	8.79<br>(42)	10.16<br>(5)	9.44<br>(18)	9.63<br>(8)	9.43<br>(16)

Table S4. [N=500, $\langle k\rangle$=10] Comparison of the original QSN and the QSN with redirected edges in terms of structural controllability (SC).

SC	R_N	TB_N	TD_N	R_E	TB_E	TD_E	Average
QSN	10.52 (3)	9.31 (13)	8.36 (57)	10.63 (21)	8.54 (49)	10.59 (17)	9.66 (27)
QSN p_{re}=0.1	7.81 (50)	6.99 (59)	6.35 (148)	8.53 (200)	8.59 (399)	8.40 (394)	7.78 (208)
QSN p_{re}=0.2	6.27 (40)	5.71 (70)	3.95 (285)	6.71 (449)	6.30 (195)	5.10 (848)	5.67 (315)
QSN p_{re}=0.3	4.57 (81)	4.86 (90)	7.01 (132)	4.02 (1438)	3.54 (624)	5.00 (829)	4.84 (532)
QSN p_{re}=0.4	3.21 (137)	4.49 (205)	4.91 (274)	2.86 (2000)	5.74 (225)	2.89 (1478)	4.02 (720)
QSN p_{re}=0.5	1.82 (440)	4.30 (248)	5.16 (222)	2.07 (3463)	2.55 (2907)	2.58 (2832)	*3.08 (1685)*
QSN p_{re}=0.6	3.12 (183)	4.56 (146)	4.04 (277)	2.38 (2547)	2.98 (634)	2.38 (2656)	3.24 (1074)
QSN p_{re}=0.7	4.21 (113)	4.10 (149)	5.56 (170)	4.35 (1157)	5.14 (728)	4.66 (996)	4.67 (552)
QSN p_{re}=0.8	6.23 (38)	6.64 (27)	5.07 (164)	5.96 (507)	4.94 (593)	6.53 (579)	5.90 (318)
QSN p_{re}=0.9	8.31 (14)	6.43 (102)	6.80 (61)	8.31 (36)	8.22 (65)	8.22 (451)	7.71 (122)
QSN p_{re}=1.0	9.94 (1)	8.61 (23)	8.79 (42)	10.18 (5)	9.47 (18)	9.63 (8)	9.44 (16)

2.2 Network Size N=1000

Table S5. [N=1000, $\langle k\rangle$=6.069] Comparison of the original QSN and the QSN with redirected edges in terms of exact controllability (EC).

EC	R_N	TB_N	TD_N	R_E	TB_E	TD_E	Average
QSN	10.04 (30)	7.30 (125)	7.79 (132)	10.57 (10)	8.55 (282)	9.98 (24)	9.04 (101)
QSN p_{re}=0.1	8.40 (4)	6.89 (92)	6.05 (157)	8.36 (41)	7.31 (390)	8.61 (295)	7.60 (163)
QSN p_{re}=0.2	5.88 (9)	7.21 (32)	5.26 (156)	6.45 (111)	6.50 (225)	6.93 (150)	6.37 (114)
QSN p_{re}=0.3	4.16 (21)	5.10 (77)	5.99 (218)	4.58 (111)	4.43 (633)	4.92 (474)	4.86 (256)
QSN p_{re}=0.4	2.79 (75)	4.24 (157)	5.33 (309)	2.23 (1410)	3.16 (667)	2.33 (2034)	3.34 (775)
QSN p_{re}=0.5	1.40 (883)	3.90 (260)	5.15 (370)	1.69 (4218)	3.59 (2912)	2.84 (2157)	*3.09 (1800)*
QSN p_{re}=0.6	3.07 (14)	4.24 (459)	6.00 (175)	2.44 (854)	3.83 (482)	2.61 (2544)	3.70 (755)
QSN p_{re}=0.7	4.83 (16)	5.11 (117)	4.14 (378)	4.33 (288)	3.65 (1320)	4.03 (281)	4.35 (400)
QSN p_{re}=0.8	6.62 (13)	5.42 (88)	4.99 (236)	6.62 (46)	7.13 (123)	5.81 (351)	6.10 (143)
QSN p_{re}=0.9	8.57 (3)	7.83 (33)	8.15 (135)	8.42 (30)	8.92 (233)	9.22 (106)	8.52 (90)
QSN p_{re}=1.0	10.25 (8)	8.76 (37)	7.15 (348)	10.31 (11)	8.94 (94)	8.73 (85)	9.02 (97)

Table S6. [N=1000, $\langle k \rangle$=6.069] Comparison of the original QSN and the QSN with redirected edges in terms of structural controllability (SC).

SC	R_N	TB_N	TD_N	R_E	TB_E	TD_E	Average
QSN	10.04<br>(31)	7.30<br>(125)	7.79<br>(132)	10.58<br>(14)	8.52<br>(283)	9.98<br>(25)	9.03<br>(102)
QSN<br>p_{re}=0.1	8.39<br>(4)	6.89<br>(92)	6.05<br>(157)	8.36<br>(41)	7.28<br>(454)	8.61<br>(295)	7.60<br>(174)
QSN<br>p_{re}=0.2	5.88<br>(9)	7.20<br>(32)	5.26<br>(156)	6.45<br>(111)	6.49<br>(225)	6.92<br>(150)	6.37<br>(114)
QSN<br>p_{re}=0.3	4.16<br>(21)	5.11<br>(77)	5.99<br>(218)	4.58<br>(111)	4.43<br>(705)	4.92<br>(474)	4.86<br>(268)
QSN<br>p_{re}=0.4	2.79<br>(74)	4.24<br>(157)	5.31<br>(318)	2.22<br>(1410)	3.17<br>(690)	2.34<br>(1947)	3.34<br>(766)
QSN<br>p_{re}=0.5	1.40<br>(883)	3.90<br>(260)	5.15<br>(364)	1.69<br>(4219)	3.60<br>(2971)	2.83<br>(2227)	*3.09*<br>*(1821)*
QSN<br>p_{re}=0.6	3.09<br>(12)	4.24<br>(458)	6.00<br>(174)	2.44<br>(853)	3.86<br>(482)	2.61<br>(2575)	3.71<br>(759)
QSN<br>p_{re}=0.7	4.84<br>(16)	5.11<br>(117)	4.15<br>(376)	4.33<br>(288)	3.67<br>(1170)	4.03<br>(281)	4.36<br>(375)
QSN<br>p_{re}=0.8	6.61<br>(13)	5.42<br>(88)	4.99<br>(236)	6.62<br>(46)	7.13<br>(125)	5.81<br>(351)	6.10<br>(143)
QSN<br>p_{re}=0.9	8.57<br>(3)	7.83<br>(33)	8.15<br>(135)	8.42<br>(30)	8.89<br>(239)	9.22<br>(106)	8.51<br>(91)
QSN<br>p_{re}=1.0	10.25<br>(8)	8.76<br>(37)	7.16<br>(348)	10.29<br>(11)	8.96<br>(61)	8.73<br>(85)	9.03<br>(92)

Table S7. [N=1000, $\langle k \rangle$=10] Comparison of the original QSN and the QSN with redirected edges in terms of exact controllability (EC).

EC	R_N	TB_N	TD_N	R_E	TB_E	TD_E	Average
QSN	10.22<br>(2)	9.13<br>(13)	8.15<br>(266)	10.61<br>(22)	8.65<br>(83)	10.32<br>(34)	9.51<br>(70)
QSN<br>p_{re}=0.1	7.99<br>(11)	6.74<br>(50)	6.32<br>(216)	8.20<br>(242)	7.80<br>(535)	7.87<br>(405)	7.49<br>(243)
QSN<br>p_{re}=0.2	6.41<br>(47)	6.27<br>(92)	5.67<br>(265)	6.52<br>(487)	6.69<br>(328)	6.33<br>(1233)	6.31<br>(409)
QSN<br>p_{re}=0.3	4.07<br>(105)	3.93<br>(264)	4.68<br>(420)	4.66<br>(1404)	4.05<br>(602)	4.40<br>(590)	4.30<br>(564)
QSN<br>p_{re}=0.4	2.21<br>(474)	5.29<br>(210)	4.84<br>(566)	2.57<br>(3321)	3.61<br>(2827)	2.66<br>(1230)	3.53<br>(1438)
QSN<br>p_{re}=0.5	2.50<br>(373)	3.22<br>(584)	4.92<br>(408)	2.02<br>(7349)	4.59<br>(3047)	1.53<br>(9364)	*3.13*<br>*(3521)*
QSN<br>p_{re}=0.6	2.46<br>(518)	4.04<br>(365)	5.19<br>(304)	2.54<br>(3349)	4.10<br>(211)	2.99<br>(963)	3.55<br>(952)
QSN<br>p_{re}=0.7	4.83<br>(71)	5.23<br>(52)	5.08<br>(339)	3.98<br>(2152)	4.80<br>(1160)	5.26<br>(470)	4.86<br>(707)
QSN<br>p_{re}=0.8	6.18<br>(75)	6.58<br>(116)	4.98<br>(314)	5.96<br>(1527)	6.29<br>(1618)	5.90<br>(535)	5.98<br>(698)
QSN<br>p_{re}=0.9	8.61<br>(15)	7.57<br>(52)	7.60<br>(132)	8.61<br>(168)	6.89<br>(812)	8.37<br>(569)	7.94<br>(291)
QSN<br>p_{re}=1.0	10.53<br>(1)	7.99<br>(206)	8.58<br>(92)	10.35<br>(6)	8.54<br>(3)	10.37<br>(67)	9.39<br>(63)

Table S8. [N=1000, $\langle k \rangle$=10] Comparison of the original QSN and the QSN with redirected edges in terms of structural controllability (SC).

SC	R_N	TB_N	TD_N	R_E	TB_E	TD_E	Average
QSN	10.22 (2)	9.11 (14)	8.15 (266)	10.61 (22)	8.64 (83)	10.32 (37)	9.51 (71)
QSN p_{re}=0.1	7.96 (11)	6.77 (50)	6.35 (211)	8.21 (242)	7.80 (539)	7.87 (405)	7.49 (243)
QSN p_{re}=0.2	6.38 (48)	6.24 (92)	5.68 (263)	6.51 (487)	6.70 (328)	6.33 (1233)	6.31 (409)
QSN p_{re}=0.3	4.08 (105)	3.94 (264)	4.67 (423)	4.65 (1404)	4.02 (716)	4.39 (590)	4.29 (584)
QSN p_{re}=0.4	2.19 (481)	5.26 (210)	4.80 (566)	2.57 (3321)	3.59 (2797)	2.66 (1201)	3.51 (1429)
QSN p_{re}=0.5	2.48 (382)	3.23 (584)	4.90 (409)	2.02 (7348)	4.61 (3109)	1.53 (9385)	*3.13 (3536)*
QSN p_{re}=0.6	2.48 (502)	4.05 (366)	5.22 (304)	2.54 (3351)	4.11 (222)	2.99 (963)	3.57 (951)
QSN p_{re}=0.7	4.83 (70)	5.24 (52)	5.08 (339)	3.98 (2152)	4.84 (1105)	5.27 (470)	4.87 (698)
QSN p_{re}=0.8	6.21 (75)	6.59 (116)	4.99 (314)	5.97 (1527)	6.28 (1637)	5.90 (535)	5.99 (701)
QSN p_{re}=0.9	8.64 (15)	7.58 (52)	7.58 (132)	8.60 (168)	6.90 (816)	8.37 (569)	7.94 (292)
QSN p_{re}=1.0	10.53 (1)	7.99 (206)	8.58 (92)	10.34 (6)	8.52 (3)	10.37 (67)	9.39 (63)

Table S9. [N=1000, $\langle k \rangle$=20] Comparison of the original QSN and the QSN with redirected edges in terms of exact controllability (EC).

EC	R_N	TB_N	TD_N	R_E	TB_E	TD_E	Average
QSN	10.00 (4)	9.71 (22)	9.04 (109)	10.28 (34)	10.21 (18)	10.13 (35)	9.89 (37)
QSN p_{re}=0.1	8.48 (95)	6.85 (199)	7.57 (154)	7.72 (2852)	7.68 (1183)	8.57 (1733)	7.81 (1036)
QSN p_{re}=0.2	5.97 (318)	5.80 (226)	5.03 (483)	5.71 (7389)	4.89 (536)	6.44 (3280)	5.64 (2039)
QSN p_{re}=0.3	4.02 (575)	4.14 (405)	4.88 (610)	4.33 (9175)	5.07 (1155)	4.39 (4746)	4.47 (2778)
QSN p_{re}=0.4	3.30 (690)	4.08 (502)	5.08 (630)	2.95 (13729)	4.47 (360)	2.96 (6415)	3.81 (3721)
QSN p_{re}=0.5	3.02 (813)	3.48 (801)	4.79 (684)	2.71 (18468)	2.49 (10989)	2.04 (15683)	*3.09 (7906)*
QSN p_{re}=0.6	3.47 (667)	4.09 (342)	4.01 (784)	3.48 (10868)	3.25 (5058)	2.75 (9256)	3.51 (4496)
QSN p_{re}=0.7	4.12 (585)	5.02 (398)	4.73 (581)	4.23 (10462)	4.00 (1213)	4.32 (4593)	4.40 (2972)
QSN p_{re}=0.8	5.33 (482)	6.35 (87)	4.80 (532)	5.70 (7831)	7.61 (638)	5.85 (3752)	5.94 (2220)
QSN p_{re}=0.9	7.58 (150)	7.54 (85)	6.22 (320)	8.20 (3274)	6.81 (522)	8.31 (1729)	7.44 (1013)
QSN p_{re}=1.0	10.72 (2)	8.95 (120)	9.87 (47)	10.68 (13)	9.53 (13)	10.26 (9)	10.00 (34)

Table S10. [N=1000, $\langle k \rangle$=20] Comparison of the original QSN and the QSN with redirected edges in terms of structural controllability (SC).

SC	R_N	TB_N	TD_N	R_E	TB_E	TD_E	Average
QSN	10.01<br>(4)	9.70<br>(24)	9.06<br>(109)	10.30<br>(34)	10.21<br>(18)	10.12<br>(36)	9.90<br>(38)
QSN<br>p_{re}=0.1	8.49<br>(95)	6.87<br>(201)	7.58<br>(148)	7.72<br>(2852)	7.67<br>(1275)	8.57<br>(1733)	7.82<br>(1051)
QSN<br>p_{re}=0.2	5.96<br>(318)	5.80<br>(226)	5.04<br>(474)	5.71<br>(7389)	4.84<br>(948)	6.44<br>(3280)	5.63<br>(2106)
QSN<br>p_{re}=0.3	4.01<br>(575)	4.15<br>(405)	4.88<br>(610)	4.33<br>(9175)	5.11<br>(1150)	4.39<br>(4746)	4.48<br>(2777)
QSN<br>p_{re}=0.4	3.31<br>(688)	4.09<br>(497)	5.07<br>(630)	2.95<br>(13719)	4.44<br>(360)	2.96<br>(6409)	3.80<br>(3717)
QSN<br>p_{re}=0.5	3.02<br>(812)	3.47<br>(808)	4.81<br>(669)	2.71<br>(18487)	2.51<br>(10989)	2.04<br>(15709)	*3.09*<br>*(7912)*
QSN<br>p_{re}=0.6	3.47<br>(666)	4.10<br>(340)	3.95<br>(800)	3.48<br>(10868)	3.29<br>(5066)	2.74<br>(9241)	3.50<br>(4497)
QSN<br>p_{re}=0.7	4.13<br>(585)	5.03<br>(398)	4.69<br>(583)	4.22<br>(10462)	4.01<br>(1121)	4.31<br>(4593)	4.40<br>(2957)
QSN<br>p_{re}=0.8	5.33<br>(482)	6.36<br>(87)	4.82<br>(532)	5.70<br>(7831)	7.61<br>(634)	5.85<br>(3768)	5.95<br>(2222)
QSN<br>p_{re}=0.9	7.57<br>(150)	7.49<br>(85)	6.24<br>(321)	8.21<br>(3274)	6.79<br>(341)	8.31<br>(1731)	7.43<br>(984)
QSN<br>p_{re}=1.0	10.72<br>(2)	8.95<br>(117)	9.85<br>(47)	10.67<br>(13)	9.53<br>(13)	10.27<br>(9)	10.00<br>(34)

2.3 Network Size N=2000

Table S11. [N=2000, $\langle k \rangle$=6.759] Comparison of the original QSN and the QSN with redirected edges in terms of exact controllability (EC).

EC	R_N	TB_N	TD_N	R_E	TB_E	TD_E	Average
QSN	10.16<br>(2)	7.74<br>(214)	7.66<br>(615)	10.08<br>(1)	9.24<br>(3)	9.34<br>(5)	9.04<br>(140)
QSN<br>p_{re}=0.1	8.80<br>(6)	6.56<br>(139)	7.56<br>(265)	8.50<br>(3)	7.33<br>(7)	7.81<br>(12)	7.76<br>(72)
QSN<br>p_{re}=0.2	6.30<br>(5)	5.69<br>(229)	6.94<br>(290)	6.51<br>(3)	5.40<br>(6)	5.79<br>(10)	6.10<br>(91)
QSN<br>p_{re}=0.3	4.89<br>(15)	3.80<br>(258)	4.90<br>(564)	4.50<br>(4)	3.62<br>(46)	3.93<br>(31)	4.27<br>(153)
QSN<br>p_{re}=0.4	2.38<br>(241)	4.71<br>(401)	5.70<br>(601)	2.33<br>(28)	2.69<br>(37)	2.61<br>(42)	3.41<br>(225)
QSN<br>p_{re}=0.5	1.55<br>(1363)	2.91<br>(1034)	4.10<br>(1057)	1.48<br>(184)	2.76<br>(96)	2.03<br>(144)	*2.47*<br>*(646)*
QSN<br>p_{re}=0.6	2.35<br>(420)	6.56<br>(40)	5.15<br>(550)	2.88<br>(17)	3.56<br>(32)	3.16<br>(35)	3.94<br>(182)
QSN<br>p_{re}=0.7	4.54<br>(13)	5.30<br>(117)	3.78<br>(560)	4.22<br>(19)	4.97<br>(3)	5.08<br>(11)	4.65<br>(121)
QSN<br>p_{re}=0.8	6.30<br>(38)	6.06<br>(91)	5.55<br>(299)	6.42<br>(2)	6.89<br>(23)	6.95<br>(5)	6.36<br>(76)
QSN<br>p_{re}=0.9	8.14<br>(1)	6.94<br>(199)	6.65<br>(262)	8.24<br>(2)	8.80<br>(2)	8.66<br>(4)	7.91<br>(78)
QSN<br>p_{re}=1.0	10.59<br>(1)	9.72<br>(22)	8.00<br>(315)	10.82<br>(1)	10.74<br>(1)	10.65<br>(1)	10.08<br>(57)

Table S12. [N=2000, $\langle k \rangle$=6.759] Comparison of the original QSN and the QSN with redirected edges in terms of structural controllability (SC).

**SC**	R_N	TB_N	TD_N	R_E	TB_E	TD_E	**Average**
QSN	10.16 (2)	7.74 (214)	7.64 (615)	10.08 (1)	9.24 (3)	9.34 (5)	9.03 (140)
QSN p_{re}=0.1	8.80 (6)	6.53 (139)	7.55 (265)	8.50 (3)	7.33 (7)	7.81 (12)	7.76 (72)
QSN p_{re}=0.2	6.29 (5)	5.70 (229)	6.94 (290)	6.50 (3)	5.40 (6)	5.80 (10)	6.10 (91)
QSN p_{re}=0.3	4.88 (15)	3.80 (258)	4.90 (564)	4.50 (4)	3.61 (46)	3.93 (31)	4.27 (153)
QSN p_{re}=0.4	2.38 (241)	4.72 (401)	5.71 (601)	2.33 (29)	2.69 (36)	2.61 (42)	3.41 (225)
QSN p_{re}=0.5	1.55 (1362)	2.91 (1034)	4.10 (1057)	1.49 (183)	2.75 (96)	2.01 (149)	*2.47 (647)*
QSN p_{re}=0.6	2.35 (420)	6.57 (40)	5.15 (550)	2.88 (17)	3.56 (29)	3.17 (35)	3.95 (182)
QSN p_{re}=0.7	4.55 (13)	5.30 (117)	3.78 (560)	4.22 (19)	4.98 (3)	5.08 (11)	4.65 (121)
QSN p_{re}=0.8	6.30 (38)	6.08 (91)	5.54 (299)	6.43 (2)	6.89 (23)	6.95 (5)	6.37 (76)
QSN p_{re}=0.9	8.13 (1)	6.94 (199)	6.66 (262)	8.24 (2)	8.80 (2)	8.66 (4)	7.91 (78)
QSN p_{re}=1.0	10.59 (1)	9.71 (22)	8.02 (315)	10.82 (1)	10.74 (1)	10.65 (1)	10.09 (57)

Table S13. [N=2000, $\langle k \rangle$=10] Comparison of the original QSN and the QSN with redirected edges in terms of exact controllability (EC).

**EC**	R_N	TB_N	TD_N	R_E	TB_E	TD_E	**Average**
QSN	10.69 (4)	7.62 (213)	8.40 (385)	10.08 (0)	9.24 (2)	9.69 (3)	9.29 (101)
QSN p_{re}=0.1	8.29 (18)	8.05 (31)	7.68 (191)	8.50 (4)	7.56 (3)	8.06 (13)	8.02 (43)
QSN p_{re}=0.2	6.25 (48)	7.16 (144)	6.15 (379)	6.37 (7)	4.97 (19)	5.97 (19)	6.14 (103)
QSN p_{re}=0.3	4.07 (310)	5.83 (241)	4.33 (661)	4.62 (22)	3.45 (49)	4.28 (25)	4.43 (218)
QSN p_{re}=0.4	2.29 (672)	4.41 (419)	3.85 (1259)	2.96 (45)	3.13 (32)	2.32 (77)	3.16 (417)
QSN p_{re}=0.5	1.67 (1570)	4.08 (964)	5.29 (692)	1.58 (173)	2.34 (88)	2.36 (93)	*2.89 (597)*
QSN p_{re}=0.6	3.41 (116)	3.45 (748)	5.06 (517)	2.38 (48)	3.92 (8)	3.00 (46)	3.54 (247)
QSN p_{re}=0.7	4.51 (165)	4.13 (287)	4.18 (782)	4.06 (31)	5.20 (9)	4.78 (16)	4.47 (215)
QSN p_{re}=0.8	6.34 (128)	5.55 (285)	5.54 (276)	6.42 (8)	6.73 (10)	6.55 (14)	6.19 (120)
QSN p_{re}=0.9	8.37 (7)	6.64 (148)	6.34 (438)	8.27 (2)	8.75 (6)	8.37 (10)	7.79 (102)
QSN p_{re}=1.0	10.12 (1)	9.08 (163)	9.17 (188)	10.76 (0)	10.73 (1)	10.64 (1)	10.08 (59)

Table S14. [N=2000, $\langle k \rangle$=10] Comparison of the original QSN and the QSN with redirected edges in terms of structural controllability (SC).

SC	R_N	TB_N	TD_N	R_E	TB_E	TD_E	Average
QSN	10.67<br>(4)	7.62<br>(206)	8.41<br>(385)	10.08<br>(0)	9.24<br>(2)	9.69<br>(3)	9.29<br>(100)
QSN<br>p_{re}=0.1	8.29<br>(18)	8.05<br>(31)	7.69<br>(191)	8.50<br>(4)	7.56<br>(3)	8.05<br>(13)	8.02<br>(43)
QSN<br>p_{re}=0.2	6.26<br>(48)	7.16<br>(144)	6.15<br>(379)	6.37<br>(7)	4.97<br>(18)	5.98<br>(19)	6.15<br>(103)
QSN<br>p_{re}=0.3	4.07<br>(310)	5.83<br>(241)	4.30<br>(661)	4.61<br>(22)	3.47<br>(46)	4.28<br>(25)	4.43<br>(218)
QSN<br>p_{re}=0.4	2.30<br>(672)	4.41<br>(420)	3.86<br>(1248)	2.95<br>(45)	3.12<br>(31)	2.32<br>(77)	3.16<br>(416)
QSN<br>p_{re}=0.5	1.67<br>(1571)	4.08<br>(968)	5.27<br>(696)	1.58<br>(173)	2.33<br>(88)	2.36<br>(94)	*2.88*<br>*(598)*
QSN<br>p_{re}=0.6	3.41<br>(115)	3.46<br>(742)	5.07<br>(517)	2.39<br>(48)	3.91<br>(8)	3.00<br>(47)	3.54<br>(246)
QSN<br>p_{re}=0.7	4.50<br>(163)	4.13<br>(287)	4.18<br>(782)	4.06<br>(31)	5.20<br>(9)	4.78<br>(16)	4.47<br>(215)
QSN<br>p_{re}=0.8	6.34<br>(128)	5.55<br>(285)	5.55<br>(276)	6.42<br>(8)	6.73<br>(9)	6.55<br>(14)	6.19<br>(120)
QSN<br>p_{re}=0.9	8.36<br>(7)	6.64<br>(158)	6.35<br>(438)	8.27<br>(2)	8.75<br>(6)	8.37<br>(10)	7.79<br>(104)
QSN<br>p_{re}=1.0	10.14<br>(1)	9.08<br>(162)	9.16<br>(188)	10.76<br>(0)	10.73<br>(1)	10.64<br>(1)	10.09<br>(59)

Table S15. [N=2000, $\langle k \rangle$=20] Comparison of the original QSN and the QSN with redirected edges in terms of exact controllability (EC).

EC	R_N	TB_N	TD_N	R_E	TB_E	TD_E	Average
QSN	10.09<br>(2)	8.71<br>(225)	8.91<br>(237)	10.11<br>(0)	9.63<br>(1)	6.92<br>(52)	9.06<br>(86)
QSN<br>p_{re}=0.1	7.88<br>(107)	7.43<br>(215)	7.63<br>(329)	8.33<br>(9)	7.05<br>(12)	9.59<br>(1)	7.99<br>(112)
QSN<br>p_{re}=0.2	5.40<br>(684)	5.55<br>(469)	5.59<br>(816)	6.11<br>(57)	5.54<br>(4)	6.56<br>(29)	5.79<br>(343)
QSN<br>p_{re}=0.3	3.86<br>(1077)	4.39<br>(581)	4.57<br>(1197)	4.27<br>(99)	3.83<br>(16)	4.77<br>(44)	4.28<br>(502)
QSN<br>p_{re}=0.4	3.15<br>(1178)	3.95<br>(1196)	4.12<br>(1338)	2.57<br>(176)	3.36<br>(40)	3.17<br>(51)	3.39<br>(663)
QSN<br>p_{re}=0.5	2.63<br>(1744)	3.87<br>(1228)	4.24<br>(1415)	2.77<br>(138)	2.34<br>(83)	2.06<br>(152)	*2.99*<br>*(793)*
QSN<br>p_{re}=0.6	2.84<br>(1316)	4.28<br>(1063)	5.15<br>(1145)	3.26<br>(108)	3.23<br>(20)	3.29<br>(46)	3.68<br>(616)
QSN<br>p_{re}=0.7	4.31<br>(999)	4.82<br>(686)	4.63<br>(1082)	4.15<br>(80)	4.50<br>(35)	4.69<br>(47)	4.52<br>(488)
QSN<br>p_{re}=0.8	6.23<br>(364)	6.11<br>(204)	4.27<br>(1113)	5.42<br>(67)	7.26<br>(3)	6.68<br>(34)	5.99<br>(298)
QSN<br>p_{re}=0.9	8.78<br>(69)	6.96<br>(266)	7.10<br>(429)	8.29<br>(10)	8.58<br>(10)	8.15<br>(19)	7.98<br>(134)
QSN<br>p_{re}=1.0	10.82<br>(1)	9.91<br>(15)	9.79<br>(102)	10.73<br>(1)	10.70<br>(1)	10.14<br>(1)	10.35<br>(20)

Table S16. [N=2000, ⟨k⟩=20] Comparison of the original QSN and the QSN with redirected edges in terms of structural controllability (SC).

SC	R_N	TB_N	TD_N	R_E	TB_E	TD_E	Average
QSN	10.10 (2)	8.69 (228)	8.91 (237)	10.12 (0)	9.64 (1)	6.89 (52)	9.06 (87)
QSN p_{re}=0.1	7.87 (107)	7.43 (207)	7.63 (329)	8.34 (9)	7.05 (12)	9.59 0	7.98 (111)
QSN p_{re}=0.2	5.41 (683)	5.56 (467)	5.59 (812)	6.10 (57)	5.55 (4)	6.56 (28)	5.80 (342)
QSN p_{re}=0.3	3.85 (1077)	4.38 (598)	4.57 (1197)	4.27 (99)	3.85 (16)	4.76 (43)	4.28 (505)
QSN p_{re}=0.4	3.15 (1180)	3.96 (1196)	4.12 (1336)	2.59 (174)	3.37 (40)	3.18 (50)	3.39 (663)
QSN p_{re}=0.5	2.64 (1736)	3.88 (1228)	4.24 (1417)	2.75 (140)	2.38 (82)	2.07 (151)	*2.99* *(792)*
QSN p_{re}=0.6	2.84 (1323)	4.26 (1063)	5.14 (1145)	3.27 (108)	3.18 (22)	3.28 (45)	3.66 (618)
QSN p_{re}=0.7	4.31 (999)	4.83 (686)	4.63 (1082)	4.14 (80)	4.50 (35)	4.71 (46)	4.52 (488)
QSN p_{re}=0.8	6.23 (364)	6.12 (204)	4.27 (1113)	5.42 (67)	7.23 (3)	6.68 (33)	5.99 (297)
QSN p_{re}=0.9	8.79 (69)	6.97 (264)	7.10 (429)	8.28 (10)	8.58 (10)	8.15 (18)	7.98 (133)
QSN p_{re}=1.0	10.81 (1)	9.91 (15)	9.79 (102)	10.71 (1)	10.69 (1)	10.14 (0)	10.34 (20)

3 QSN with p_{re}=0.5 vs. Other Network Topologies Comparison Tables

The comparisons of the overall best QSN variants (QSN with p_{re}=0.5) and other network topologies are given in Tables. S17 to S32. The network size is set to N=500, N=1000, and N=2000, respectively. For N=500, the average degree of networks is set to ⟨k⟩=6.069 and ⟨k⟩=10, respectively. For N=1000 and N=2000, the average degree is set to ⟨k⟩=6.069, ⟨k⟩=10, and ⟨k⟩=20, respectively. When ⟨k⟩=6.069, QSN with $p_{re} = 0.5$ is compared to random graph (RG), multiplex congruence network (MCN), random triangle network (RTN), and random rectangle network (RRN). When ⟨k⟩=10 and ⟨k⟩=20, QSN with p_{re}=0.5 is compared to RG, RTN, and RRN only. MCN is excluded because the average degree of an MCN cannot be set manually.

In each cell of the table, the real number represents the average rank, and the integer inside the parentheses mean the number of winning times. Italic real numbers (with gray-shaded) represent the minimum average rank, and italic numbers inside parentheses (with gray-shaded) mean the maximum average number of winning times.

3.1 Network Size N=500

Table S17. [N=500, ⟨k⟩=5.38] Comparison of robustness of exact controllability (EC) among 5 networks.

EC	R_N	TB_N	TD_N	R_E	TB_E	TD_E	Average
RG	3.07 (7)	2.38 (80)	2.69 (202)	2.54 (3)	3.72 (67)	3.36 (370)	2.96 (122)
RTN	3.73 (2)	3.75 (12)	4.49 (91)	3.84 (64)	1.97 (868)	2.55 (395)	3.39 (239)
RRN	1.95 (140)	2.34 (177)	3.45 (148)	2.76 (160)	1.61 (1481)	1.38 (2167)	2.25 (712)
MCN	4.96 (4)	4.95 (6)	2.36 (267)	4.81 (2)	5.00 (2)	4.99 (5)	4.51 (48)
QSN p_{re}=0.5	1.30 (361)	1.58 (306)	2.01 (263)	1.05 (2654)	2.71 (477)	2.72 (429)	*1.89* *(748)*

Table S18. [N=500, ⟨k⟩=5.38] Comparison of robustness of structural controllability (SC) among 5 networks.

SC	R_N	TB_N	TD_N	R_E	TB_E	TD_E	Average
RG	3.08<br>(7)	2.38<br>(80)	2.69<br>(202)	2.55<br>(3)	3.70<br>(67)	3.36<br>(370)	2.96<br>(122)
RTN	3.72<br>(2)	3.75<br>(12)	4.49<br>(91)	3.83<br>(73)	2.00<br>(868)	2.55<br>(395)	3.39<br>(240)
RRN	1.96<br>(136)	2.35<br>(170)	3.45<br>(148)	2.76<br>(160)	1.57<br>(1500)	1.38<br>(2167)	2.24<br>(714)
MCN	4.96<br>(4)	4.95<br>(6)	2.38<br>(263)	4.80<br>(2)	5.00<br>(2)	4.99<br>(5)	4.51<br>(47)
QSN<br>p_{re}=0.5	1.29<br>(367)	1.57<br>(307)	2.00<br>(264)	1.05<br>(2654)	2.73<br>(426)	2.72<br>(429)	*1.89*<br>*(741)*

Table S19. [N=500, ⟨k⟩=10] Comparison of robustness of exact controllability (EC) among 5 networks.

EC	R_N	TB_N	TD_N	R_E	TB_E	TD_E	Average
RG	2.43<br>(157)	1.85<br>(312)	2.23<br>(283)	2.12<br>(393)	3.29<br>(746)	3.46<br>(574)	2.56<br>(411)
RTN	3.31<br>(145)	3.52<br>(101)	3.46<br>(177)	3.78<br>(347)	2.57<br>(681)	2.54<br>(1074)	3.20<br>(421)
RRN	2.61<br>(176)	2.30<br>(203)	2.58<br>(237)	2.99<br>(345)	1.83<br>(2620)	1.42<br>(4376)	2.29<br>(1326)
QSN<br>p_{re}=0.5	1.65<br>(436)	2.32<br>(191)	1.73<br>(477)	1.11<br>(4998)	2.31<br>(1553)	2.58<br>(1290)	*1.95*<br>*(1491)*

Table S20. [N=500, ⟨k⟩=10] Comparison of robustness of structural controllability (SC) among 5 networks.

SC	R_N	TB_N	TD_N	R_E	TB_E	TD_E	Average
RG	2.41<br>(164)	1.87<br>(312)	2.23<br>(283)	2.12<br>(393)	3.29<br>(746)	3.46<br>(574)	2.56<br>(412)
RTN	3.31<br>(143)	3.52<br>(101)	3.46<br>(177)	3.78<br>(347)	2.58<br>(642)	2.54<br>(1074)	3.20<br>(414)
RRN	2.61<br>(176)	2.29<br>(204)	2.58<br>(237)	2.99<br>(345)	1.83<br>(2611)	1.42<br>(4376)	2.29<br>(1325)
QSN<br>p_{re}=0.5	1.67<br>(429)	2.31<br>(189)	1.73<br>(477)	1.11<br>(4998)	2.30<br>(1539)	2.58<br>(1290)	*1.95*<br>*(1487)*

3.2 Network Size N=1000

Table S21. [N=1000, $\langle k\rangle$=6.069] Comparison of robustness of exact controllability (EC) among 5 networks.

EC	R_N	TB_N	TD_N	R_E	TB_E	TD_E	Average
RG	3.02 (57)	2.71 (66)	2.95 (244)	2.34 (1)	3.28 (683)	3.54 (8)	2.97 (177)
RTN	3.50 (31)	3.25 (80)	4.40 (174)	4.04 (63)	2.28 (1237)	2.50 (1481)	3.33 (511)
RRN	2.29 (73)	2.84 (96)	2.98 (295)	2.77 (296)	1.31 (4494)	1.22 (5424)	2.24 *(1780)*
MCN	4.98 (4)	4.96 (4)	3.02 (284)	4.81 (5)	5.00 (7)	5.00 (4)	4.63 (51)
QSN p_{re}=0.5	1.21 (878)	1.24 (901)	1.65 (800)	1.03 (6051)	3.14 (77)	2.74 (470)	*1.83* (1530)

Table S22. [N=1000, $\langle k\rangle$=6.069] Comparison of robustness of structural controllability (SC) among 5 networks.

SC	R_N	TB_N	TD_N	R_E	TB_E	TD_E	Average
RG	3.02 (56)	2.72 (66)	2.95 (244)	2.34 (1)	3.28 (651)	3.55 (8)	2.98 (171)
RTN	3.50 (31)	3.24 (80)	4.40 (174)	4.05 (63)	2.27 (1237)	2.50 (1481)	3.33 (511)
RRN	2.30 (68)	2.84 (96)	2.98 (295)	2.78 (296)	1.31 (4511)	1.22 (5424)	2.24 *(1782)*
MCN	4.98 (4)	4.96 (4)	3.02 (284)	4.80 (5)	5.00 (7)	5.00 (4)	4.63 (51)
QSN p_{re}=0.5	1.20 (883)	1.24 (901)	1.65 (798)	1.03 (6051)	3.14 (77)	2.73 (470)	*1.83* (1530)

Table S23. [N=1000, $\langle k\rangle$=10] Comparison of robustness of exact controllability (EC) among 4 networks.

EC	R_N	TB_N	TD_N	R_E	TB_E	TD_E	Average
RG	1.96 (554)	1.72 (624)	1.93 (537)	3.90 (636)	3.00 (1962)	3.19 (2530)	2.62 (1141)
RTN	3.52 (192)	3.69 (104)	3.41 (275)	2.73 (1994)	2.65 (458)	2.39 (2996)	3.07 (1003)
RRN	2.27 (391)	2.61 (317)	2.98 (280)	1.67 (6073)	1.40 (7434)	2.03 (4744)	*2.16 (3207)*
QSN p_{re}=0.5	2.25 (461)	1.99 (470)	1.67 (840)	1.70 (4990)	2.96 (436)	2.39 (3912)	*2.16* (1852)

Table S24. [N=1000, $\langle k \rangle$=10] Comparison of robustness of structural controllability (SC) among 4 networks.

**SC**	**R$_N$**	**TB$_N$**	**TD$_N$**	**R$_E$**	**TB$_E$**	**TD$_E$**	**Average**
RG	1.97 (551)	1.72 (625)	1.93 (537)	3.90 (636)	3.00 (1958)	3.19 (2530)	2.62 (1140)
RTN	3.53 (192)	3.69 (104)	3.41 (275)	2.73 (1994)	2.64 (482)	2.40 (2996)	3.07 (1007)
RRN	2.25 (398)	2.60 (319)	2.98 (280)	1.67 (6073)	1.40 (7471)	2.03 (4744)	*2.16 (3214)*
QSN p_{re}=0.5	2.26 (454)	1.99 (466)	1.67 (840)	1.70 (4990)	2.96 (486)	2.38 (3912)	*2.16* (1858)

Table S25. [N=1000, $\langle k \rangle$=20] Comparison of robustness of exact controllability (EC) among 4 networks.

**EC**	**R$_N$**	**TB$_N$**	**TD$_N$**	**R$_E$**	**TB$_E$**	**TD$_E$**	**Average**
RG	2.48 (727)	2.10 (649)	1.90 (999)	2.24 (1192)	3.67 (199)	2.72 (7177)	2.52 (1824)
RTN	2.92 (631)	3.04 (445)	3.09 (565)	3.91 (1171)	2.61 (1688)	2.07 (7479)	2.94 (1997)
RRN	2.42 (713)	2.96 (352)	2.73 (597)	2.75 (1261)	1.54 (11622)	2.18 (11641)	2.43 (4364)
QSN p_{re}=0.5	2.19 (797)	1.91 (798)	2.27 (640)	1.09 (19998)	2.17 (8566)	3.03 (6304)	*2.11 (6184)*

Table S26. [N=1000, $\langle k \rangle$=20] Comparison of robustness of structural controllability (SC) among 4 networks.

**SC**	**R$_N$**	**TB$_N$**	**TD$_N$**	**R$_E$**	**TB$_E$**	**TD$_E$**	**Average**
RG	2.47 (726)	2.10 (648)	1.90 (999)	2.24 (1192)	3.68 (146)	2.72 (7177)	2.52 (1815)
RTN	2.93 (631)	3.04 (445)	3.09 (565)	3.91 (1171)	2.61 (1780)	2.07 (7499)	2.94 (2015)
RRN	2.41 (708)	2.96 (352)	2.73 (597)	2.75 (1261)	1.52 (12254)	2.18 (11641)	2.43 (4469)
QSN p_{re}=0.5	2.18 (803)	1.91 (801)	2.27 (640)	1.09 (19998)	2.19 (8421)	3.03 (6304)	*2.11 (6161)*

3.3 Network Size N=2000

Table S27. [N=2000, $\langle k \rangle$=6.759] Comparison of robustness of exact controllability (EC) among 5 networks.

EC	R_N	TB_N	TD_N	R_E	TB_E	TD_E	Average
RG	3.21<br>(57)	2.41<br>(321)	2.61<br>(304)	3.32<br>(1)	3.89<br>(1)	3.77<br>(1)	3.20<br>(114)
RTN	3.67<br>(34)	3.55<br>(51)	4.28<br>(304)	3.60<br>(8)	1.89<br>(49)	1.79<br>(64)	3.13<br>(85)
RRN	1.78<br>(601)	2.69<br>(456)	3.14<br>(462)	1.93<br>(27)	1.22<br>(179)	1.25<br>(186)	2.00<br>(319)
MCN	4.99<br>(1)	4.97<br>(19)	3.61<br>(363)	4.99<br>(1)	4.99<br>(1)	4.99<br>(1)	4.76<br>(64)
QSN<br>p_{re}=0.5	1.36<br>(1409)	1.38<br>(1456)	1.37<br>(1959)	1.16<br>(208)	3.01<br>(2)	3.20<br>(7)	*1.91*<br>*(840)*

Table S28. [N=2000, $\langle k \rangle$=6.759] Comparison of robustness of structural controllability (SC) among 5 networks.

SC	R_N	TB_N	TD_N	R_E	TB_E	TD_E	Average
RG	3.21<br>(56)	2.41<br>(321)	2.60<br>(304)	3.32<br>(1)	3.89<br>(1)	3.77<br>(1)	3.20<br>(114)
RTN	3.67<br>(34)	3.55<br>(51)	4.28<br>(304)	3.60<br>(8)	1.89<br>(49)	1.79<br>(64)	3.13<br>(85)
RRN	1.76<br>(629)	2.69<br>(456)	3.14<br>(462)	1.93<br>(27)	1.22<br>(179)	1.25<br>(186)	2.00<br>(323)
MCN	4.99<br>(1)	4.97<br>(19)	3.61<br>(363)	4.99<br>(1)	4.99<br>(1)	4.99<br>(1)	4.76<br>(64)
QSN<br>p_{re}=0.5	1.37<br>(1384)	1.38<br>(1456)	1.37<br>(1959)	1.16<br>(208)	3.01<br>(2)	3.20<br>(7)	*1.92*<br>*(836)*

Table S29. [N=2000, $\langle k \rangle$=10] Comparison of robustness of exact controllability (EC) among 4 networks.

EC	R_N	TB_N	TD_N	R_E	TB_E	TD_E	Average
RG	3.12<br>(130)	2.28<br>(494)	1.52<br>(1761)	2.35<br>(64)	3.68<br>(1)	3.50<br>(35)	2.74<br>(414)
RTN	3.64<br>(266)	3.58<br>(168)	3.51<br>(419)	3.85<br>(17)	2.02<br>(39)	1.76<br>(92)	3.06<br>(167)
RRN	1.64<br>(1124)	2.69<br>(632)	2.88<br>(510)	2.42<br>(24)	1.19<br>(168)	1.55<br>(119)	*2.06*<br>(430)
QSN<br>p_{re}=0.5	1.59<br>(1195)	1.46<br>(1583)	2.08<br>(923)	1.38<br>(175)	3.11<br>(2)	3.20<br>(8)	2.14<br>*(648)*

Table S30. [N=2000, $\langle k \rangle$=10] Comparison of robustness of structural controllability (SC) among 4 networks.

**SC**	**R_N**	**TB_N**	**TD_N**	**R_E**	**TB_E**	**TD_E**	**Average**
RG	3.12 (130)	2.28 (494)	1.53 (1756)	2.35 (64)	3.68 (1)	3.50 (35)	2.74 (413)
RTN	3.65 (266)	3.58 (168)	3.51 (419)	3.85 (17)	2.01 (39)	1.76 (92)	3.06 (167)
RRN	1.65 (1117)	2.69 (632)	2.88 (510)	2.42 (24)	1.19 (168)	1.55 (119)	*2.06* (428)
QSN p_{re}=0.5	1.59 (1204)	1.46 (1583)	2.08 (947)	1.38 (175)	3.12 (2)	3.20 (8)	2.14 *(653)*

Table S31. [N=2000, $\langle k \rangle$=20] Comparison of robustness of exact controllability (EC) among 4 networks.

**EC**	**R_N**	**TB_N**	**TD_N**	**R_E**	**TB_E**	**TD_E**	**Average**
RG	2.65 (1055)	1.97 (1542)	2.14 (1351)	2.00 (168)	3.37 (2)	2.88 (51)	2.50 (695)
RTN	2.85 (1198)	3.22 (766)	3.46 (719)	3.27 (98)	2.27 (9)	2.05 (77)	2.85 (478)
RRN	2.37 (1406)	2.46 (1052)	2.60 (1169)	2.17 (146)	1.05 (194)	2.01 (112)	*2.11* (680)
QSN p_{re}=0.5	2.12 (1497)	2.36 (1179)	1.81 (1987)	2.57 (120)	3.32 (2)	3.06 (64)	2.54 *(808)*

Table S32. [N=2000, $\langle k \rangle$=20] Comparison of robustness of structural controllability (SC) among 4 networks.

**SC**	**R_N**	**TB_N**	**TD_N**	**R_E**	**TB_E**	**TD_E**	**Average**
RG	2.66 (1052)	1.97 (1532)	2.14 (1351)	2.00 (168)	3.36 (2)	2.88 (51)	2.50 (693)
RTN	2.86 (1197)	3.22 (766)	3.46 (719)	3.27 (98)	2.26 (11)	2.05 (77)	2.85 (478)
RRN	2.36 (1407)	2.46 (1052)	2.60 (1169)	2.16 (147)	1.05 (194)	2.01 (112)	*2.11* (680)
QSN p_{re}=0.5	2.12 (1499)	2.36 (1179)	1.81 (1987)	2.57 (120)	3.33 (2)	3.06 (64)	2.54 *(809)*

4 QSN vs. QSN Variants Comparison Curves

The comparison curves of controllability robustness are shown in the following figures. The left subplot (a) shows the results of exact controllability (EC); and the right subplot (b) shows the results of structural controllability (SC).

4.1 Network Size N=500

4.1.1 Edge Random Attack

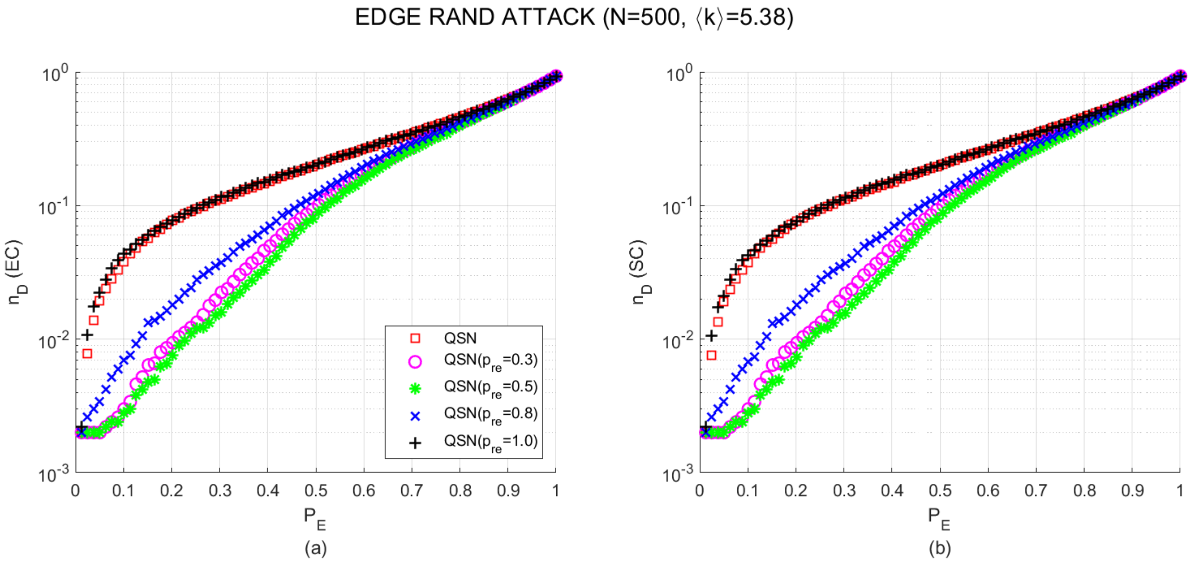

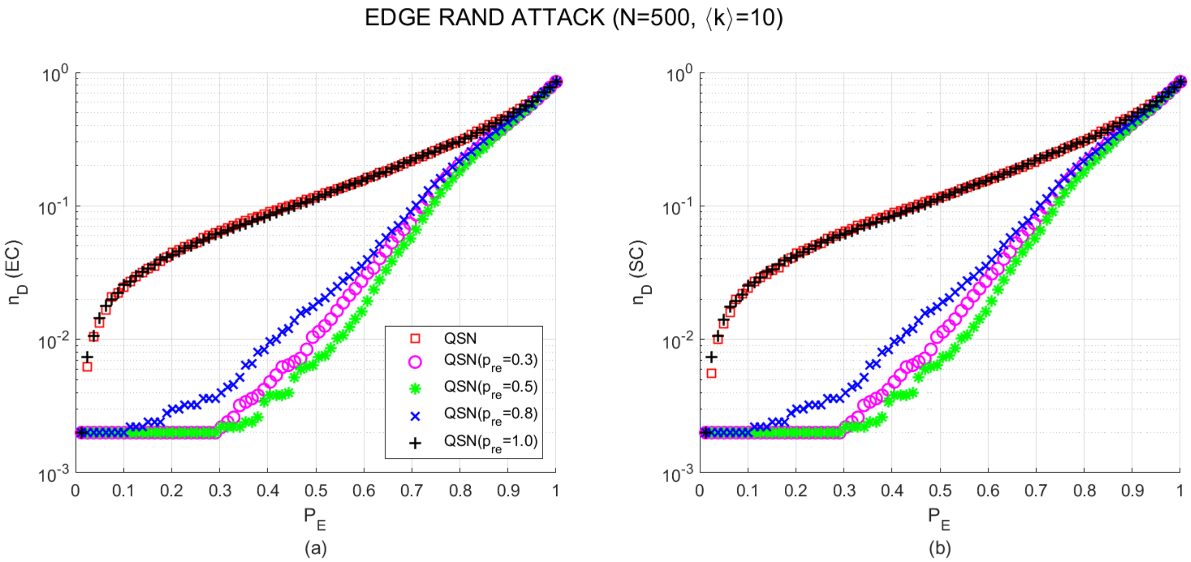

4.1.2 Edge Intentional (Betweenness-based) Attack

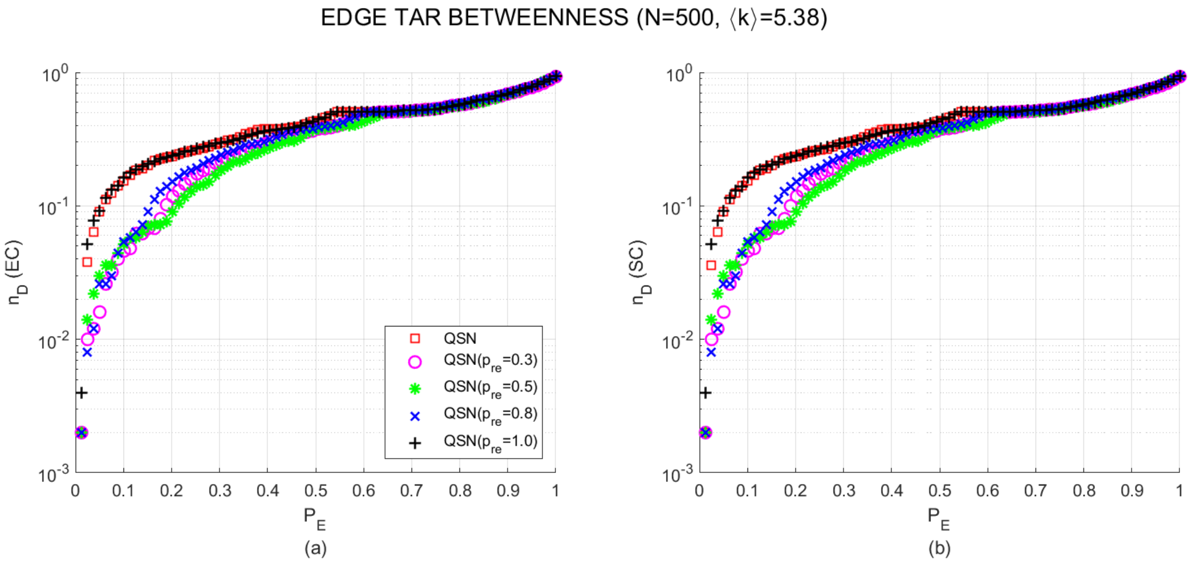

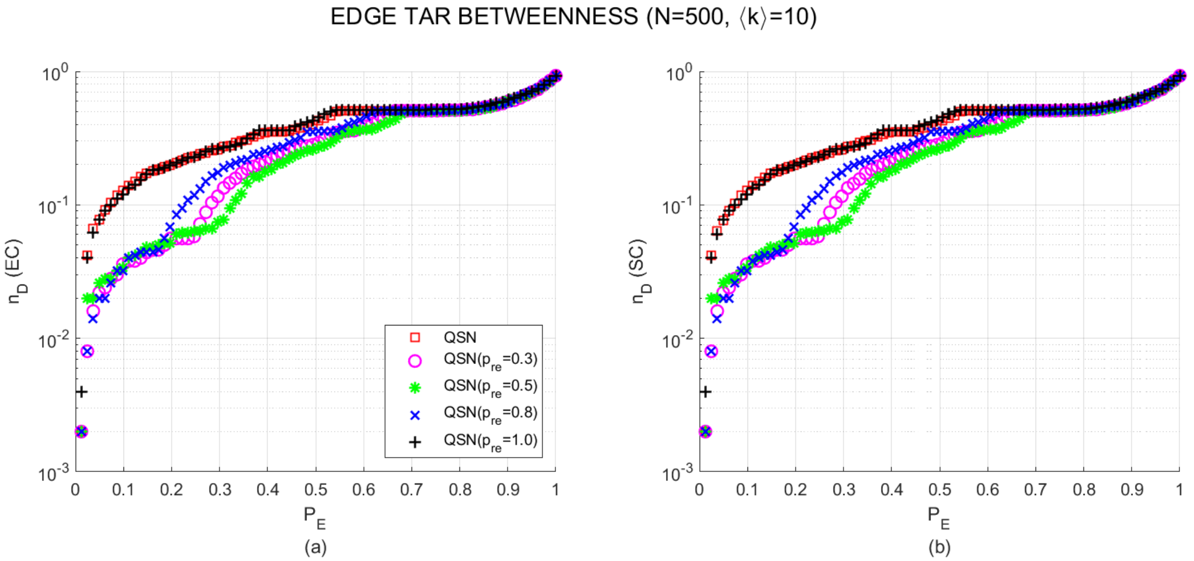

4.1.3 Edge Intentional (Degree-based) Attack

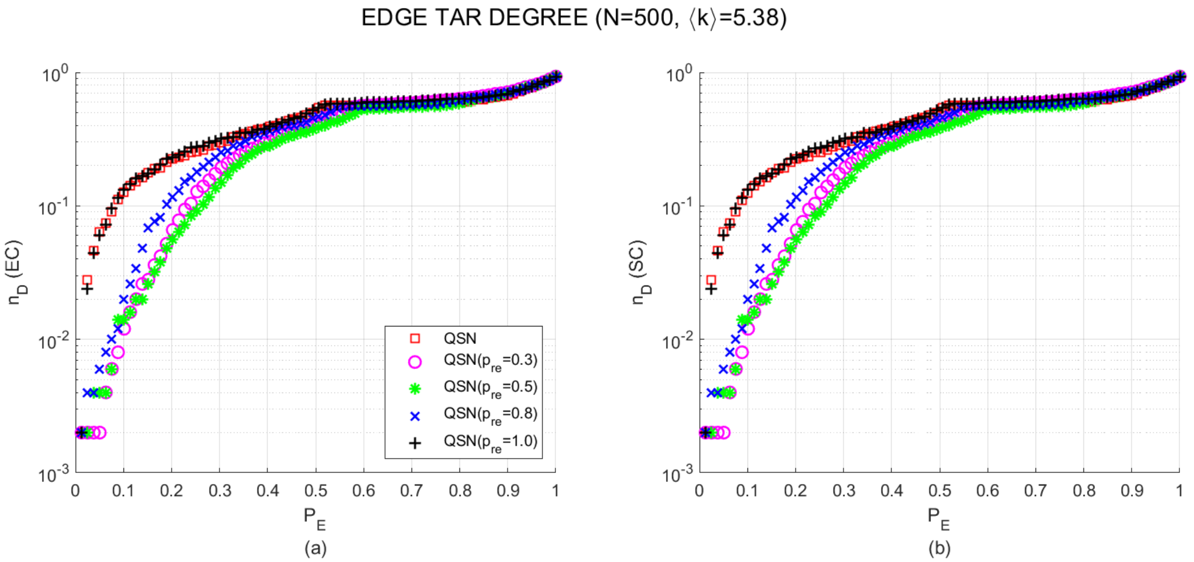

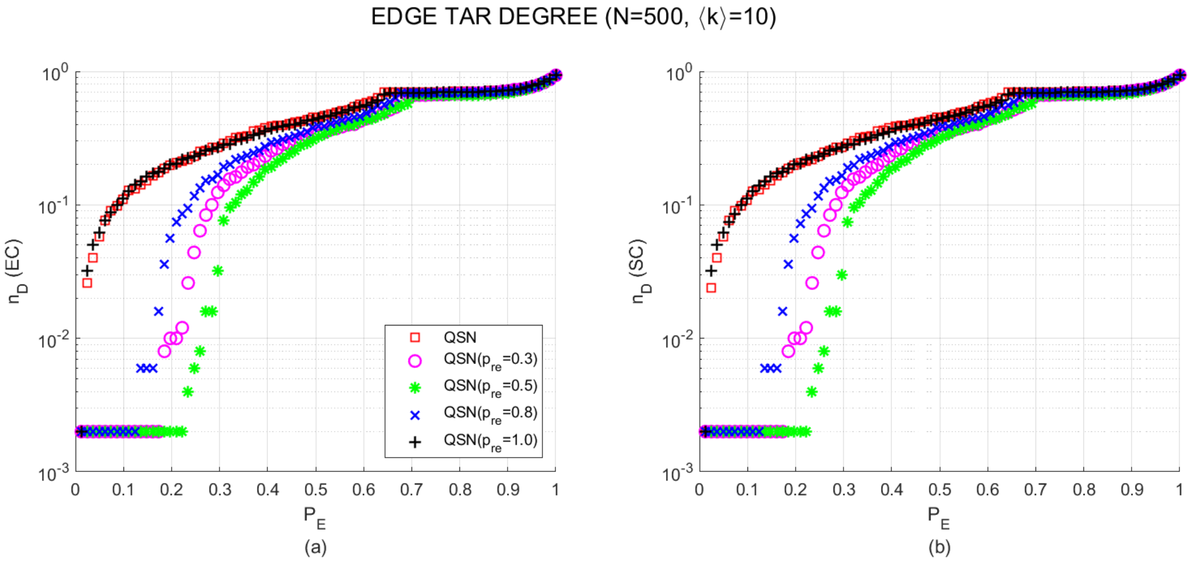

4.1.4 *Node Random Attack*

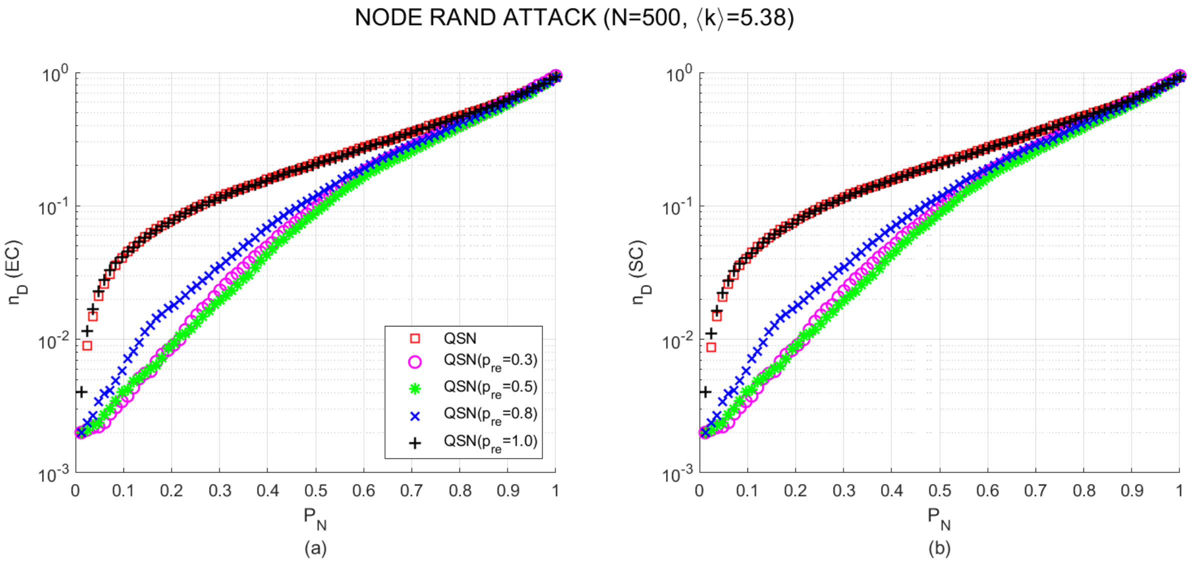

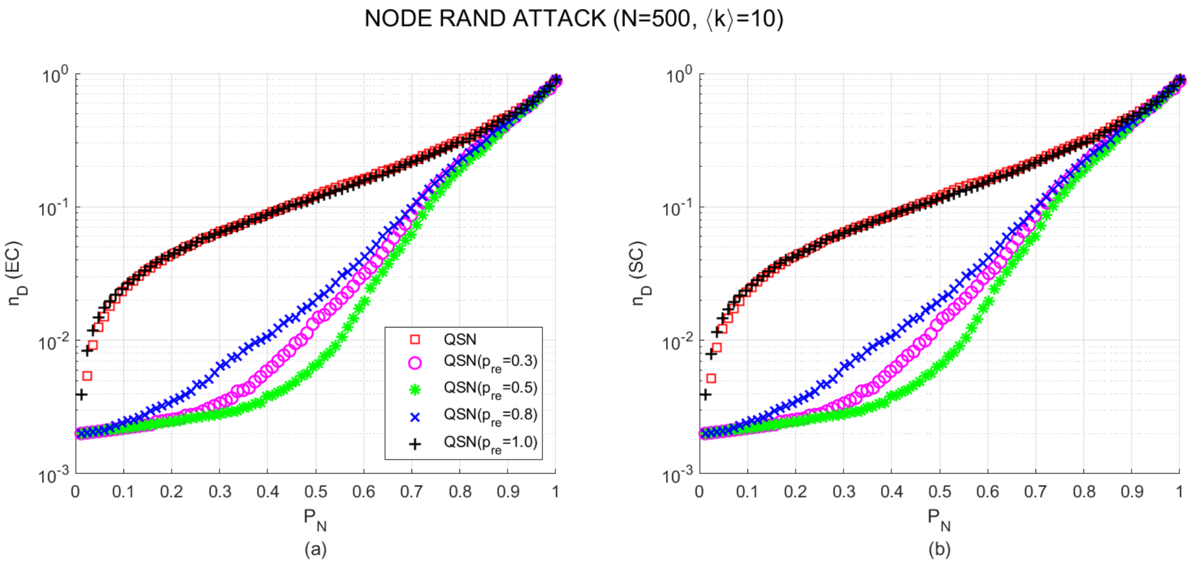

4.1.5 Node Intentional (Betweenness-based) Attack

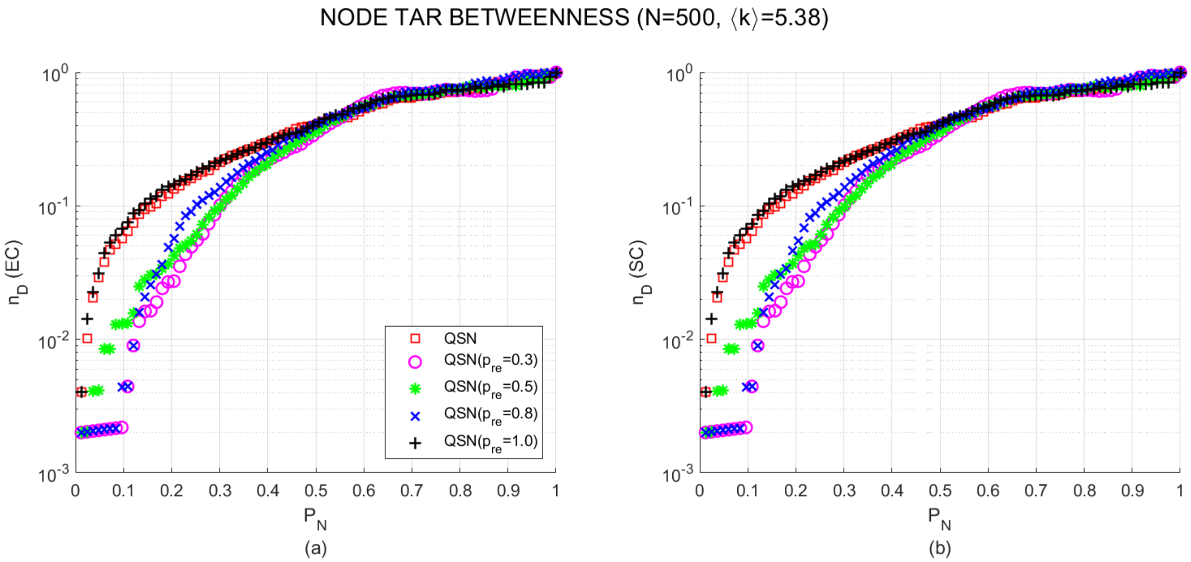

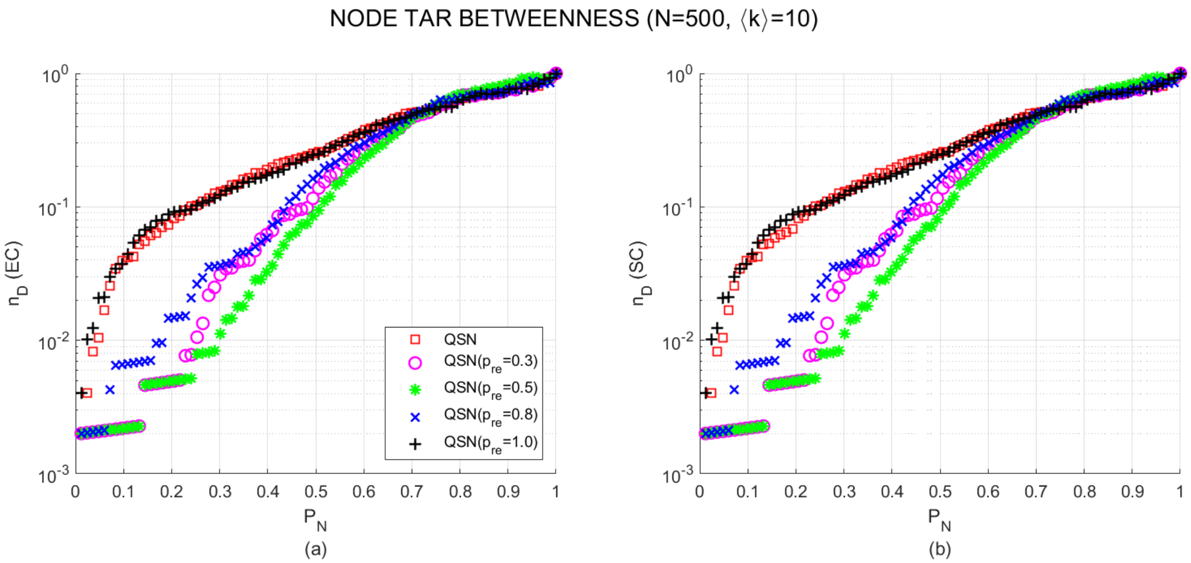

4.1.6 Node Intentional (Degree-based) Attack

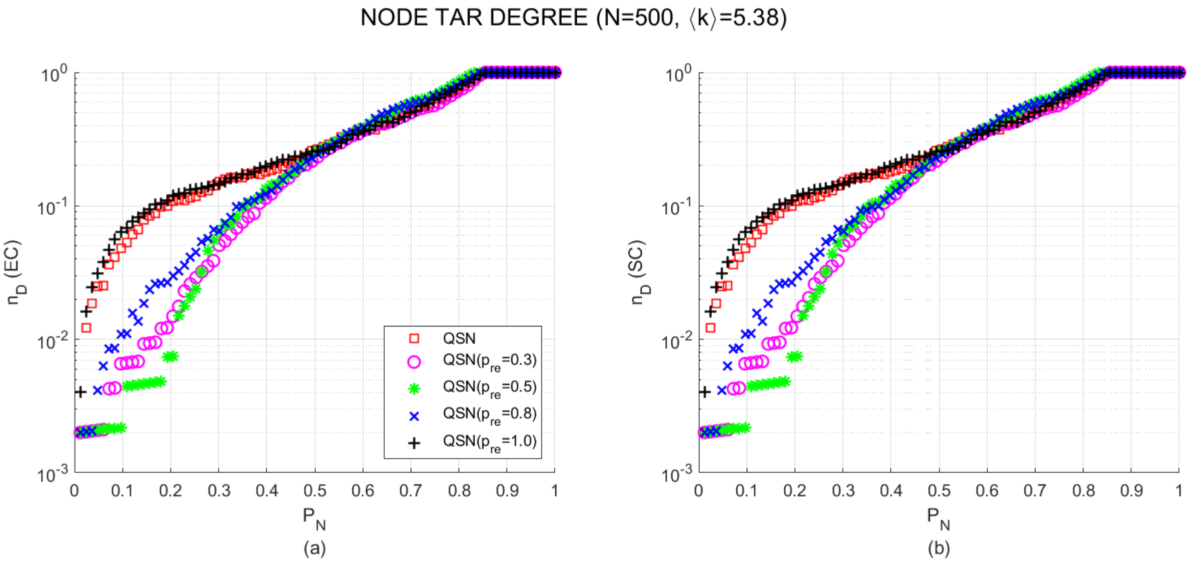

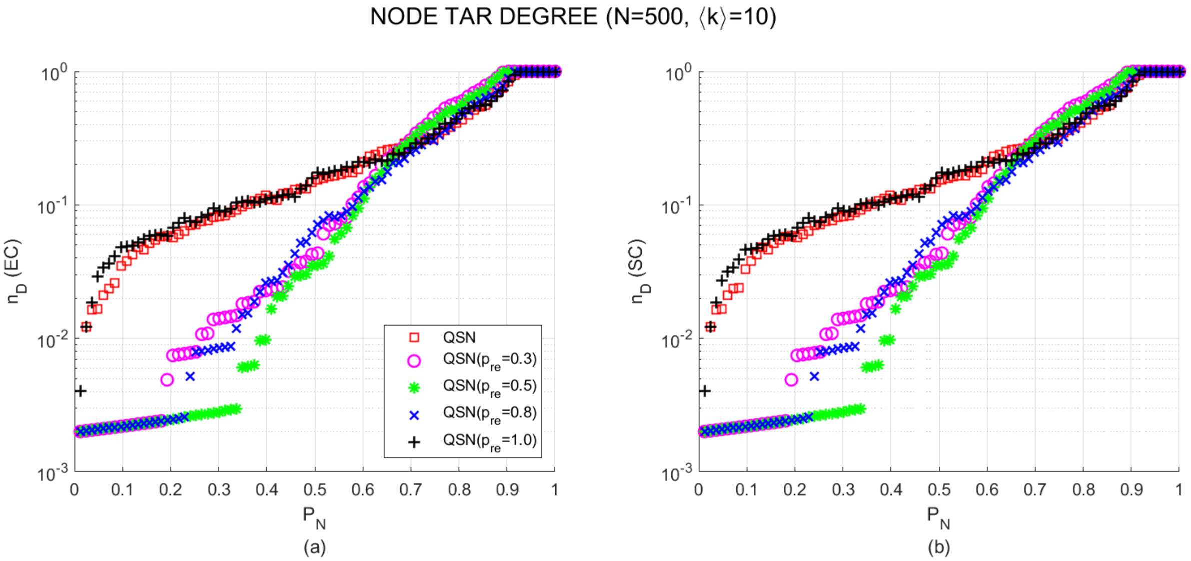

4.2 Network Size N=1000

4.2.1 Edge Random Attack

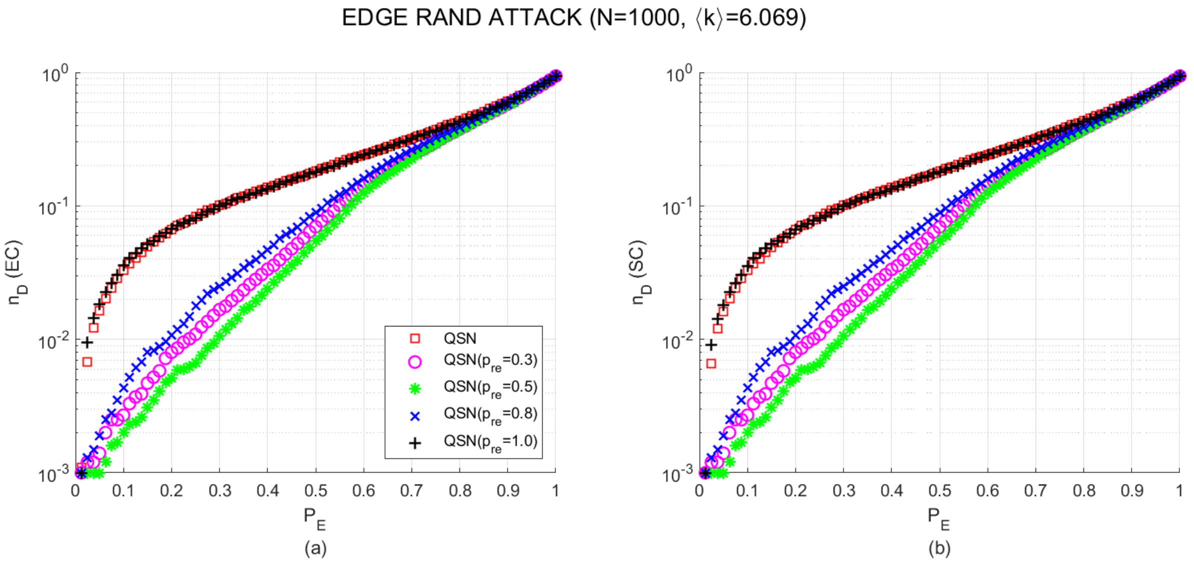

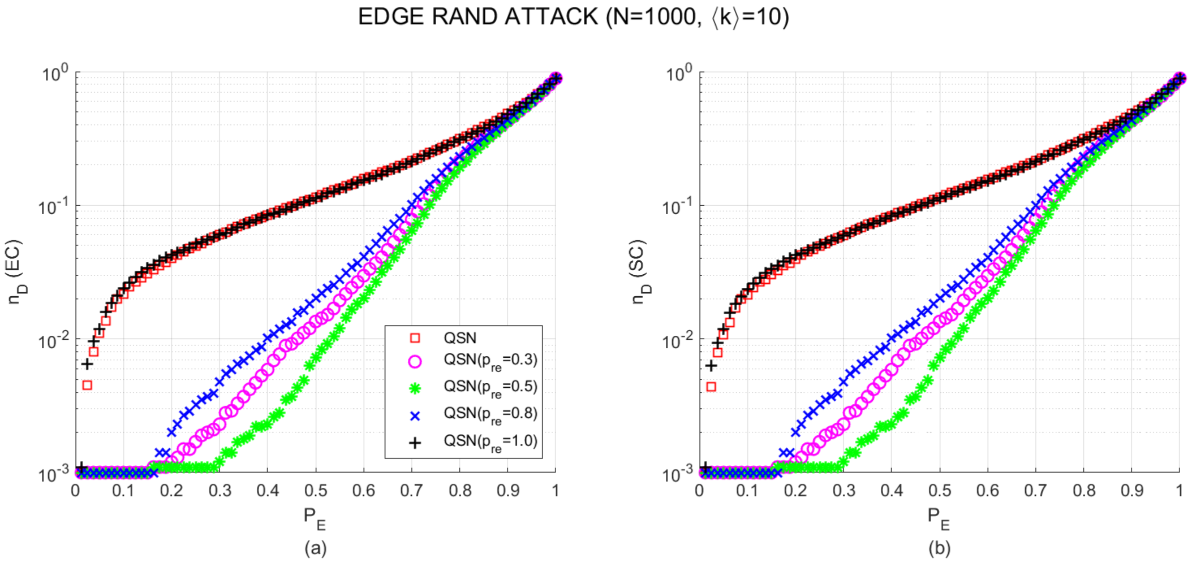

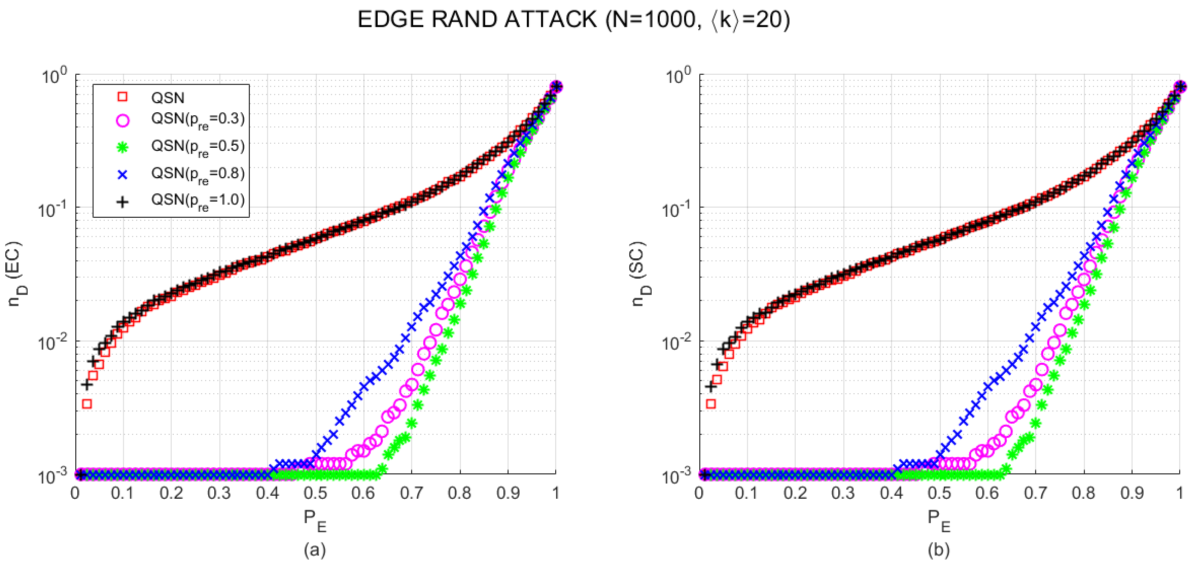
EDGE RAND ATTACK (N=1000, ⟨k⟩=20)
QSN
QSN(p_re=0.3)
QSN(p_re=0.5)
QSN(p_re=0.8)
QSN(p_re=1.0)
n_D (EC)
n_D (SC)
P_E
(a)
(b)

4.2.2 *Edge Intentional (Betweenness-based) Attack*

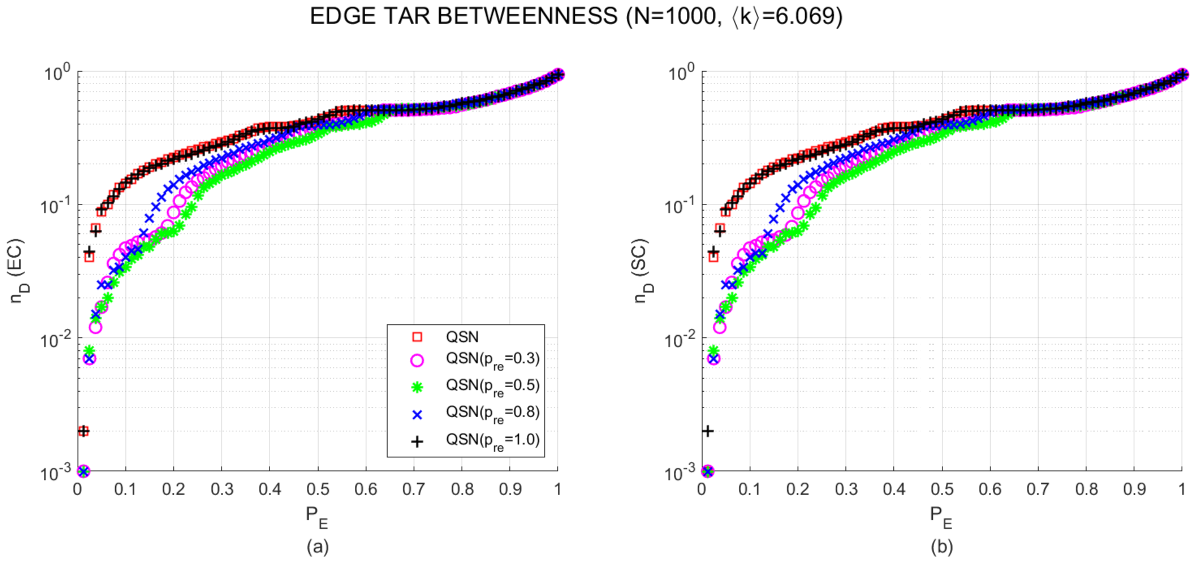

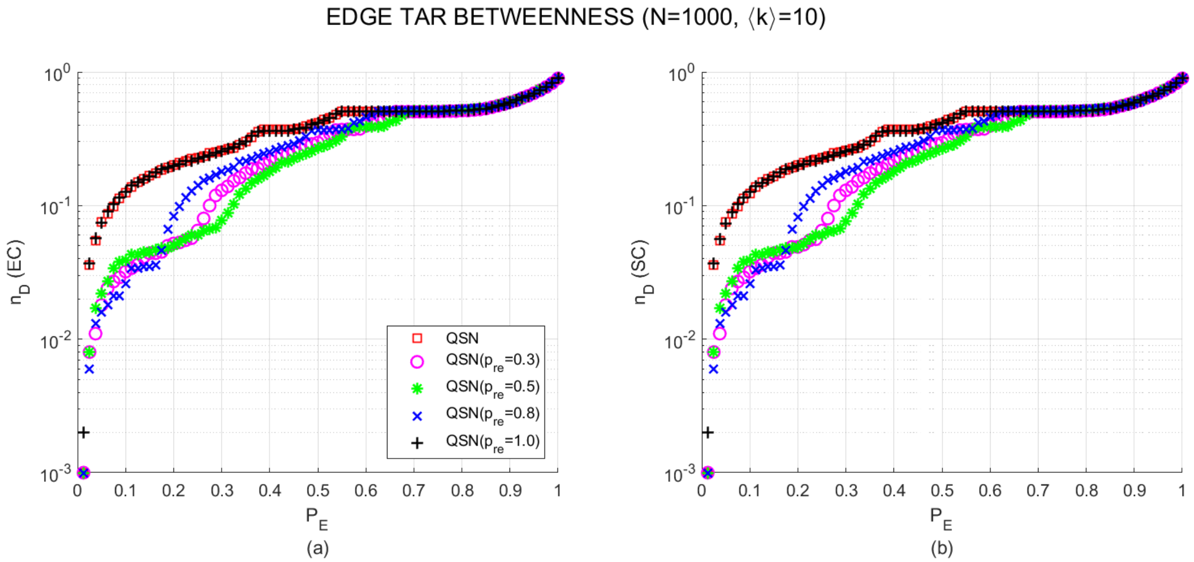

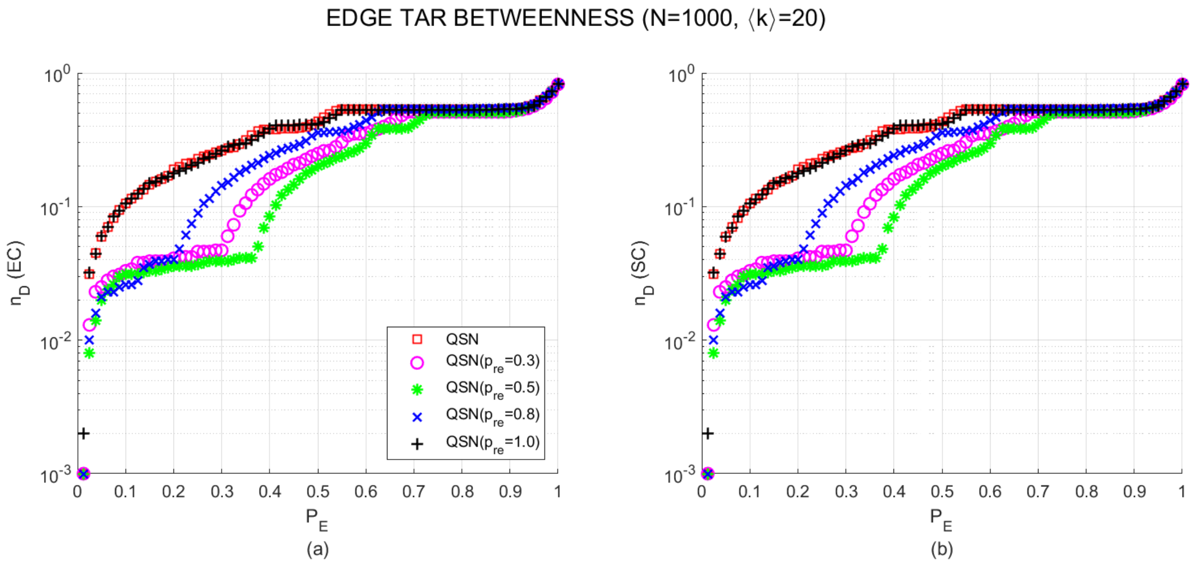

4.2.3 *Edge Intentional (Degree-based) Attack*

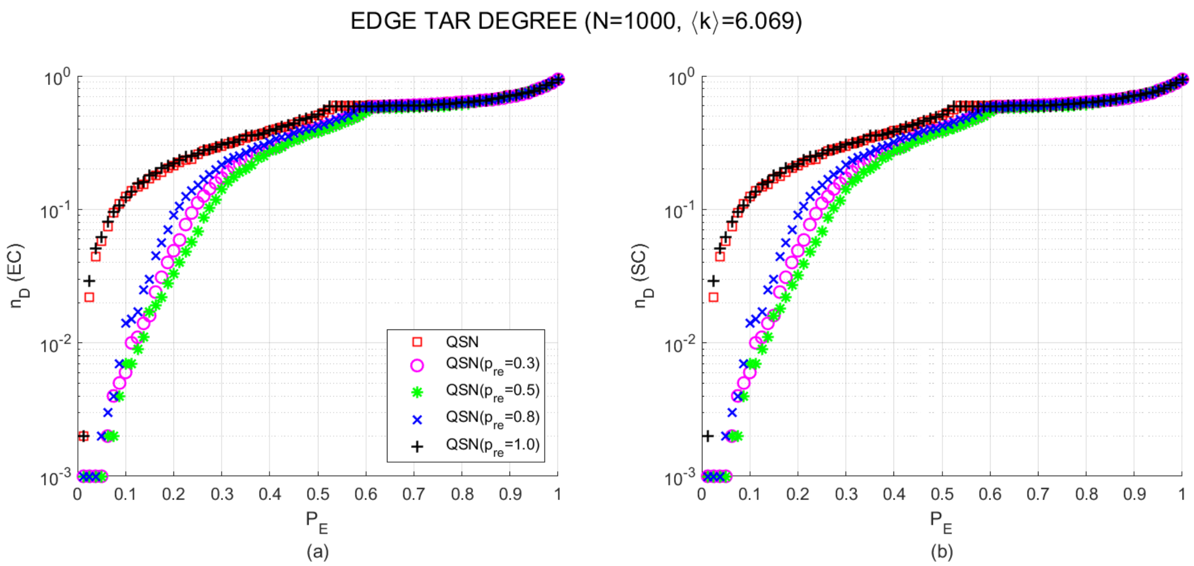

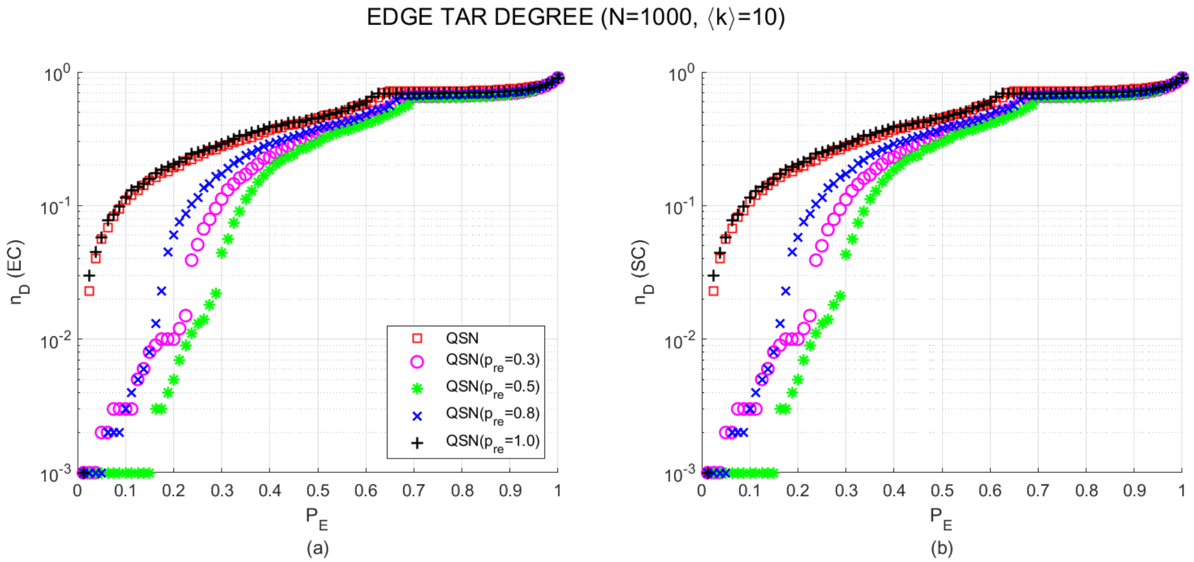

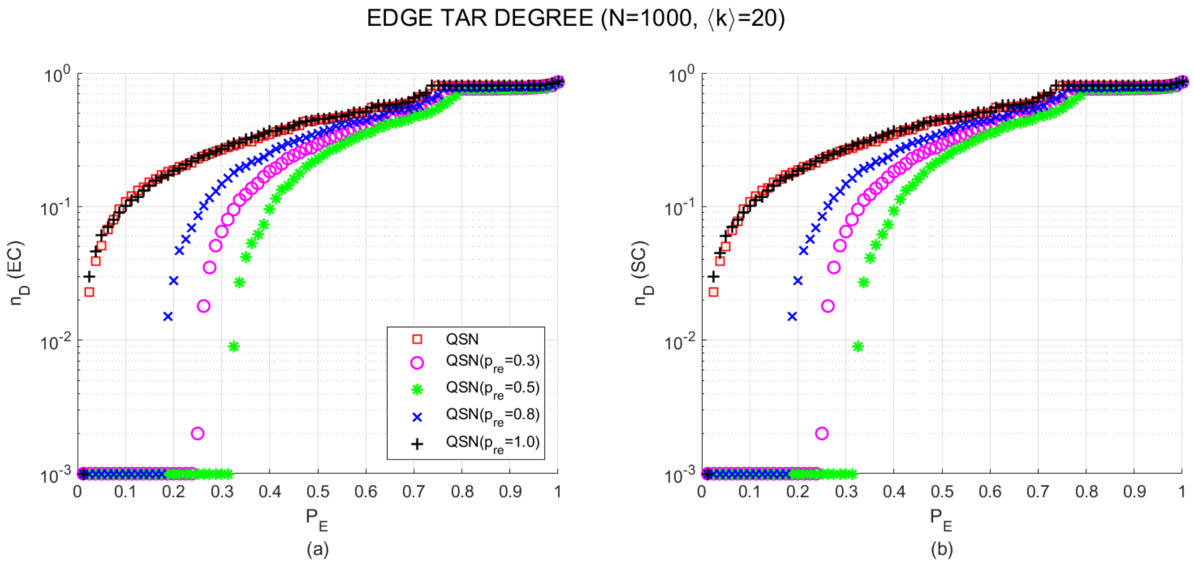

4.2.4 Node Random Attack

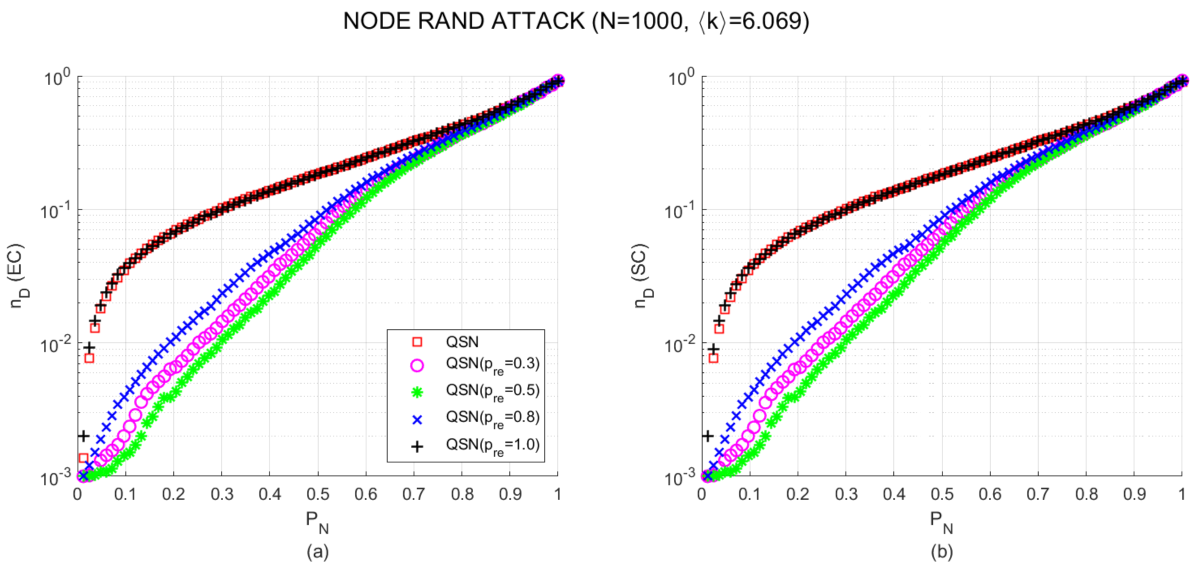

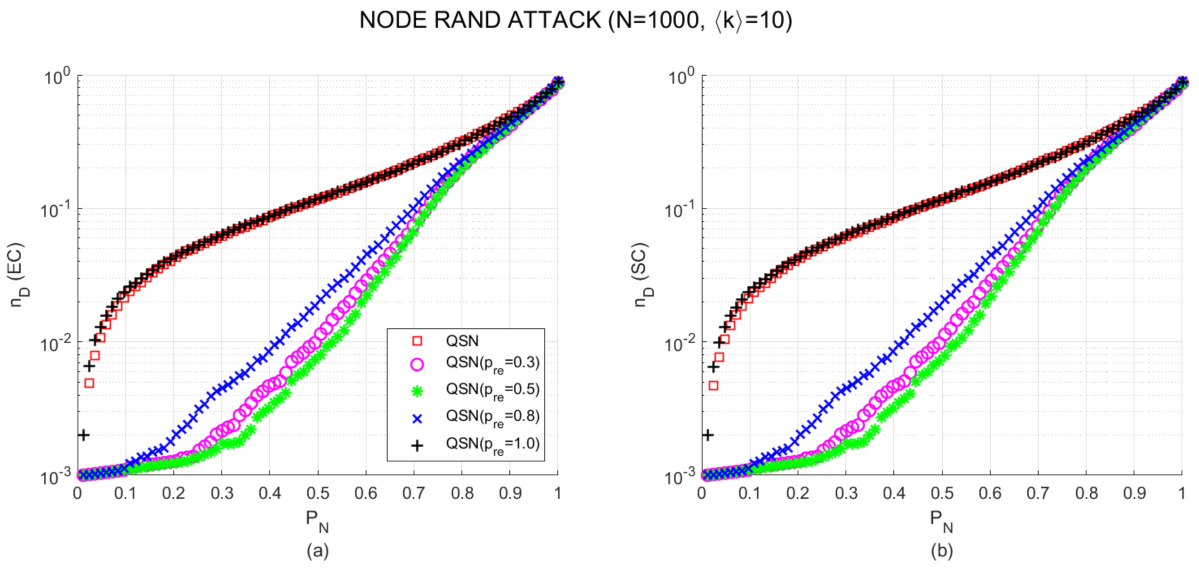

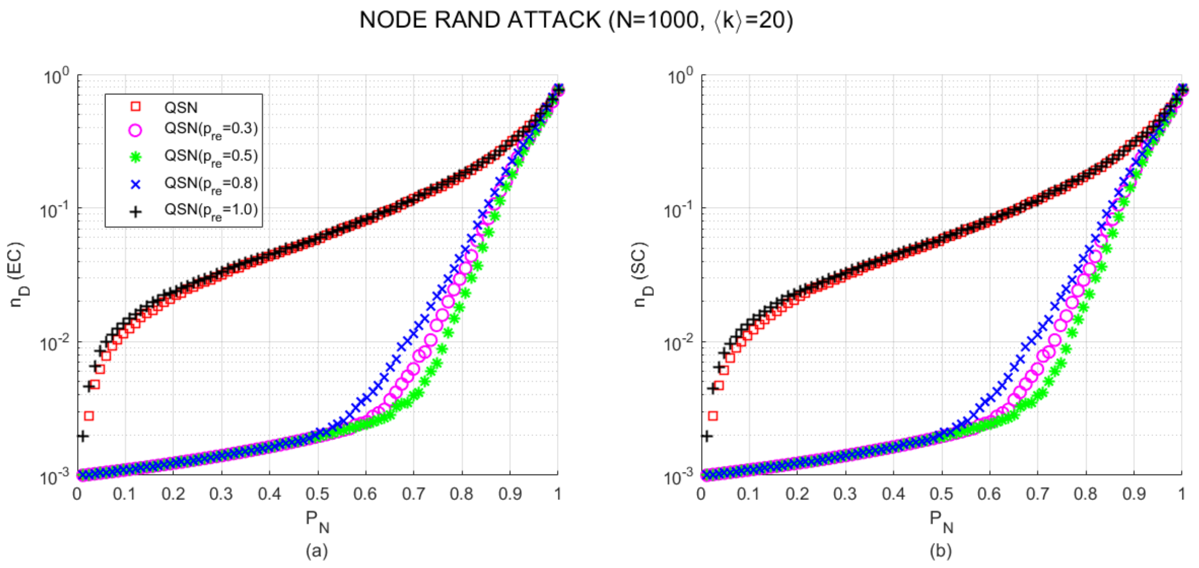

4.2.5 *Node Intentional (Betweenness-based) Attack*

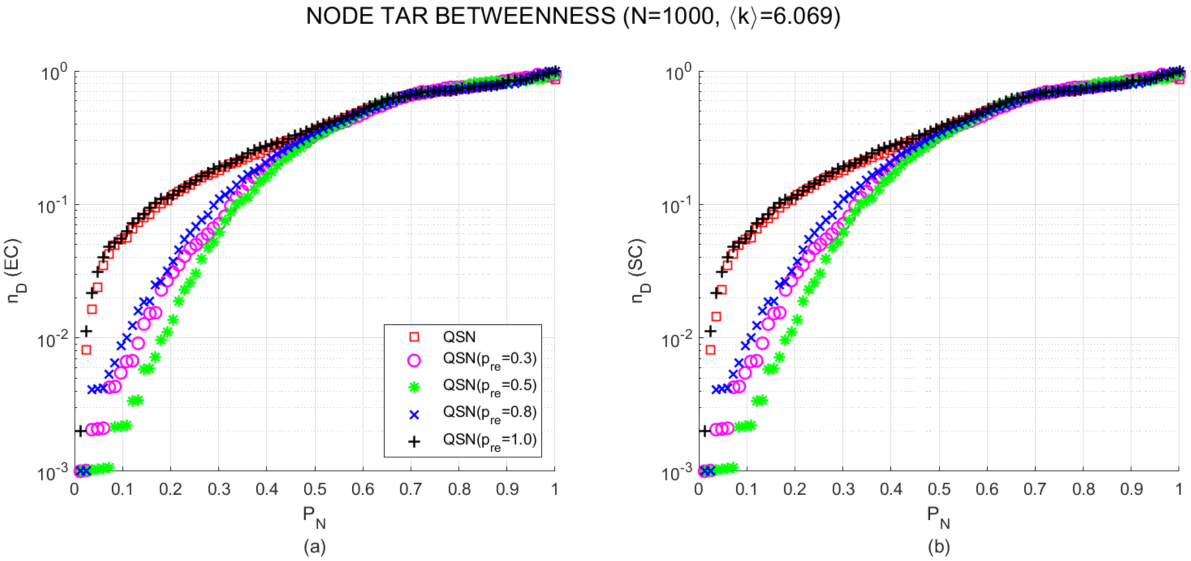

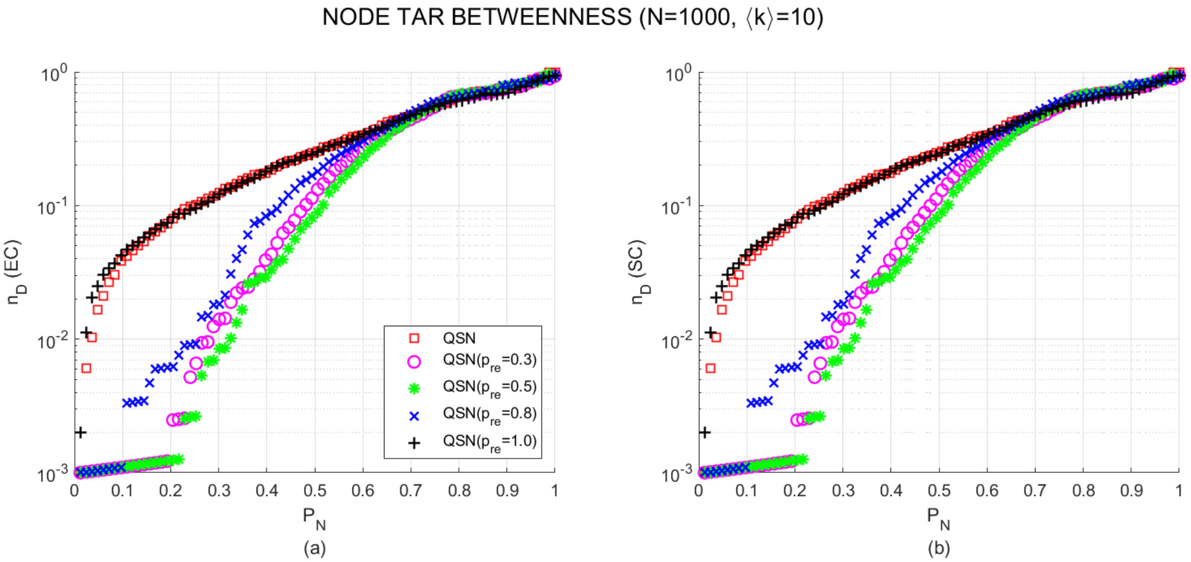

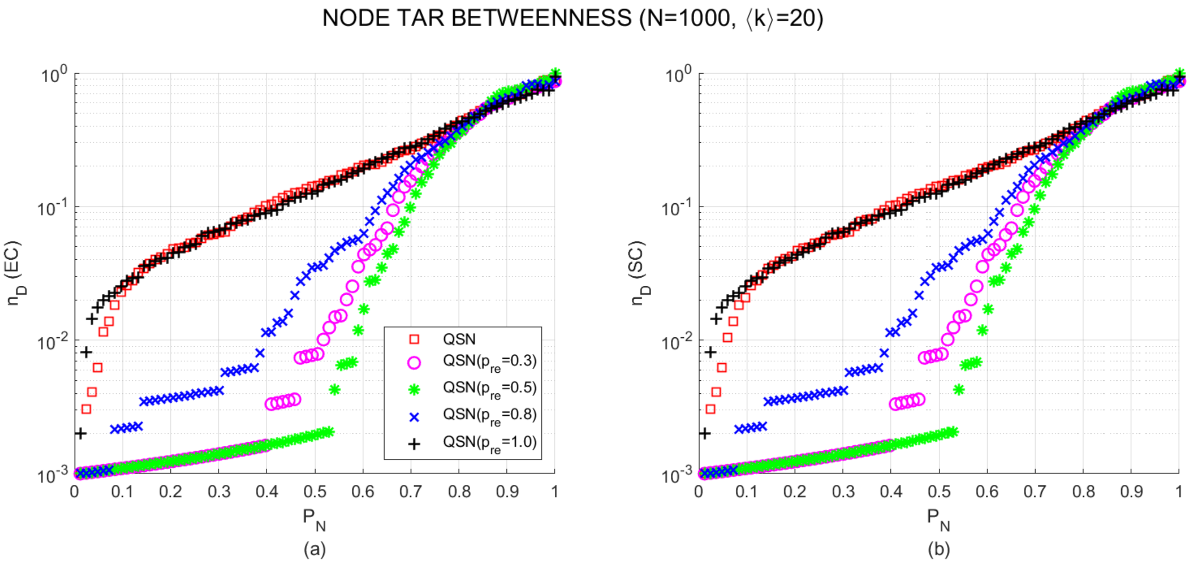

4.2.6 *Node Intentional (Degree-based) Attack*

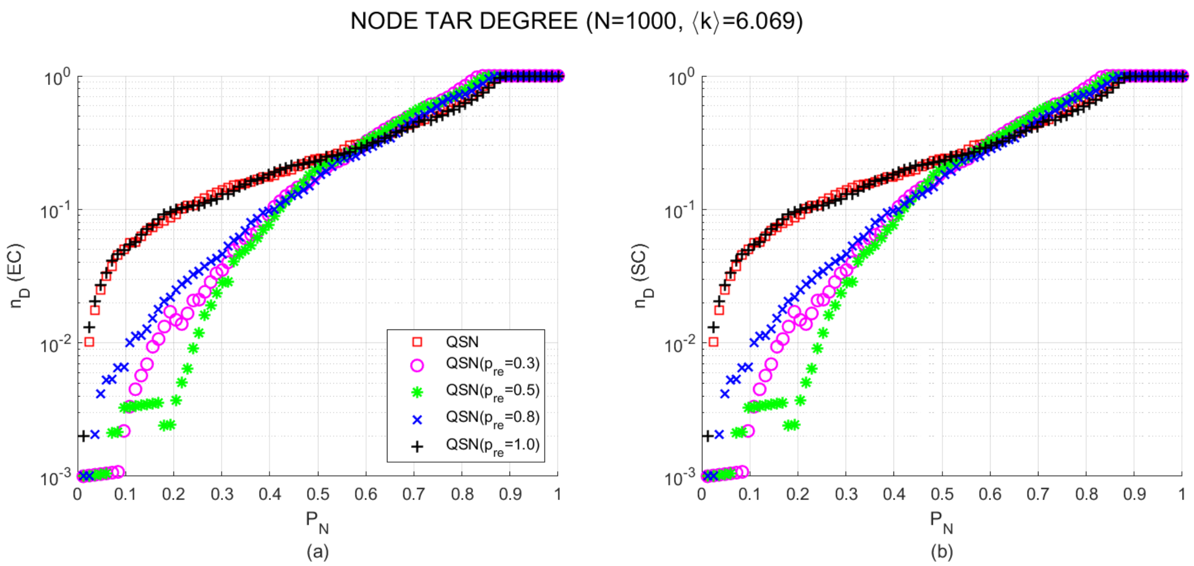

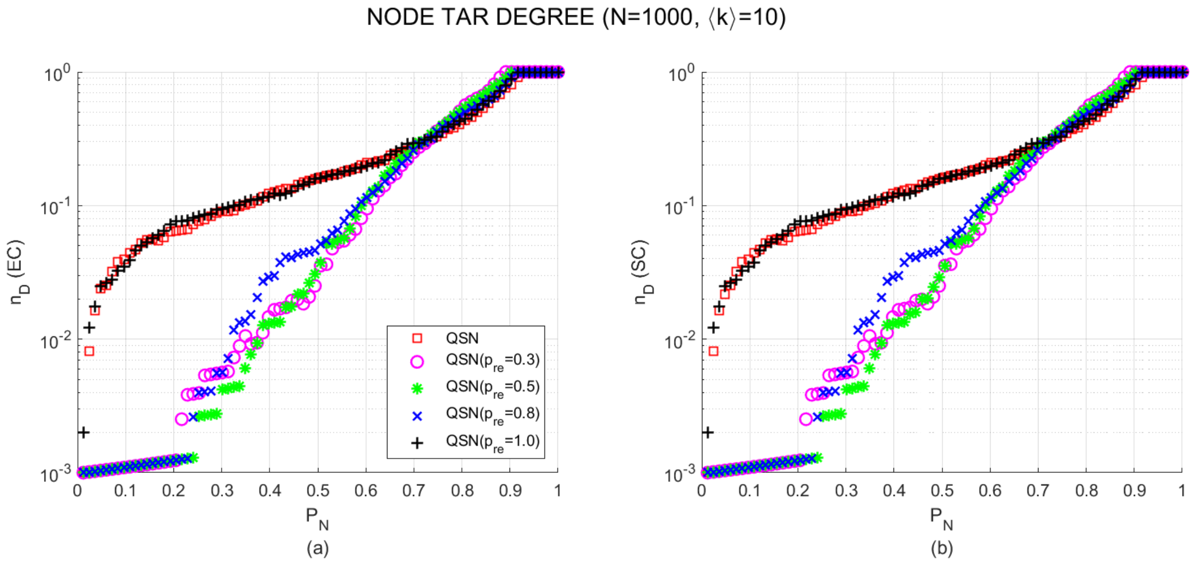

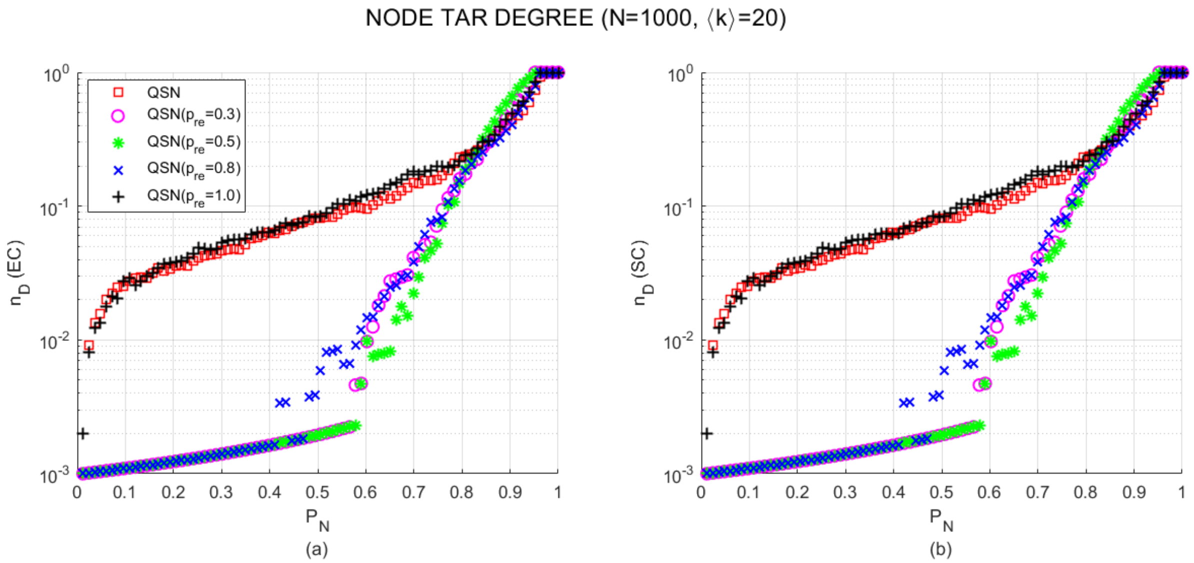

4.3 Network Size N=2000

4.3.1 Edge Random Attack

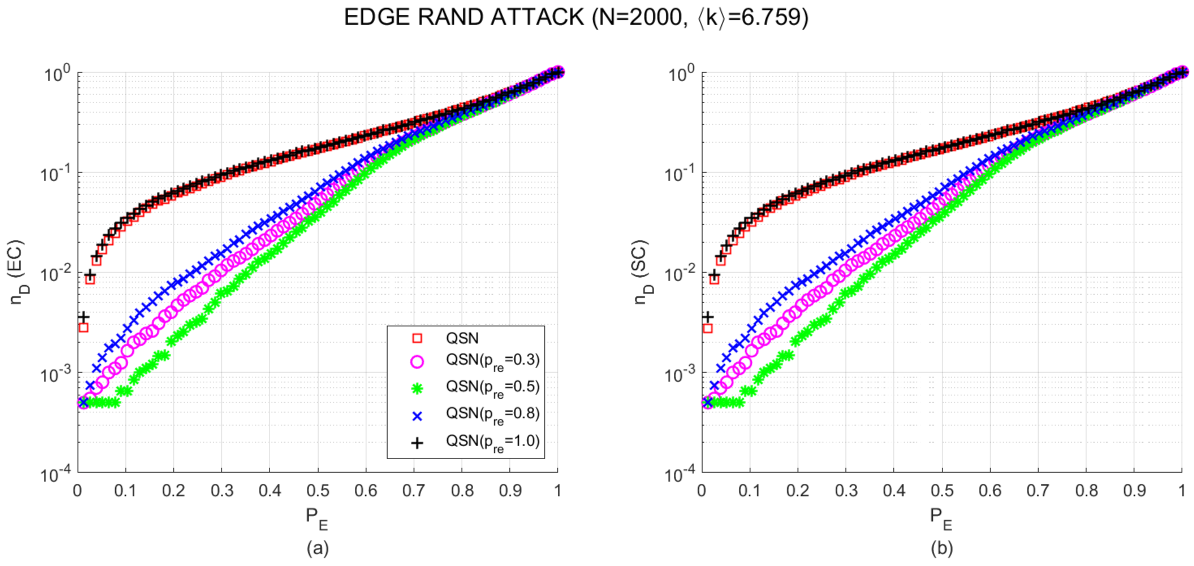

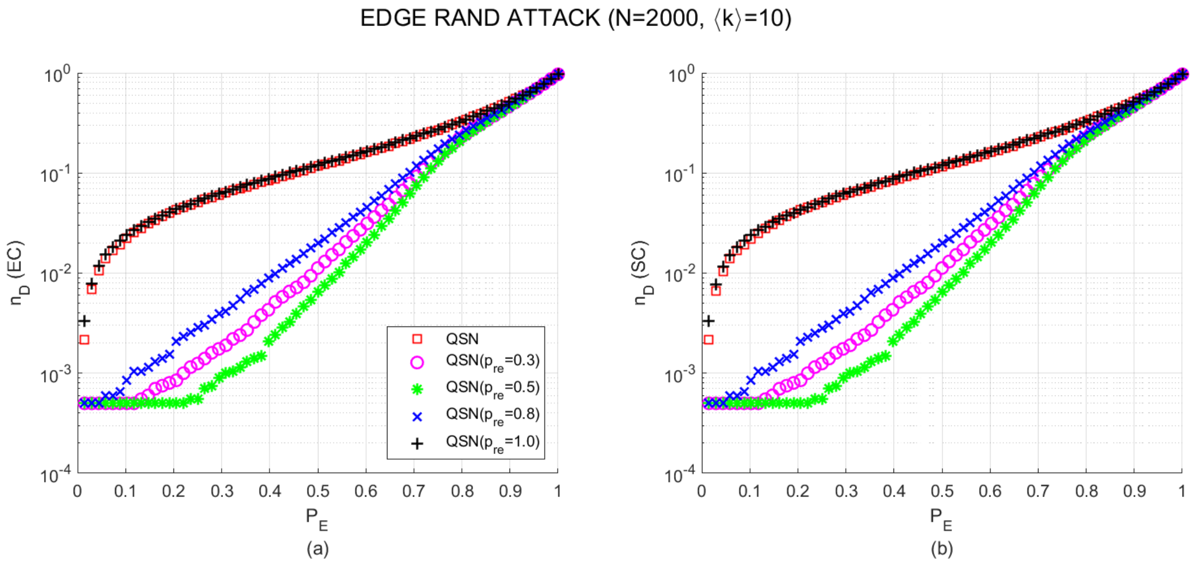

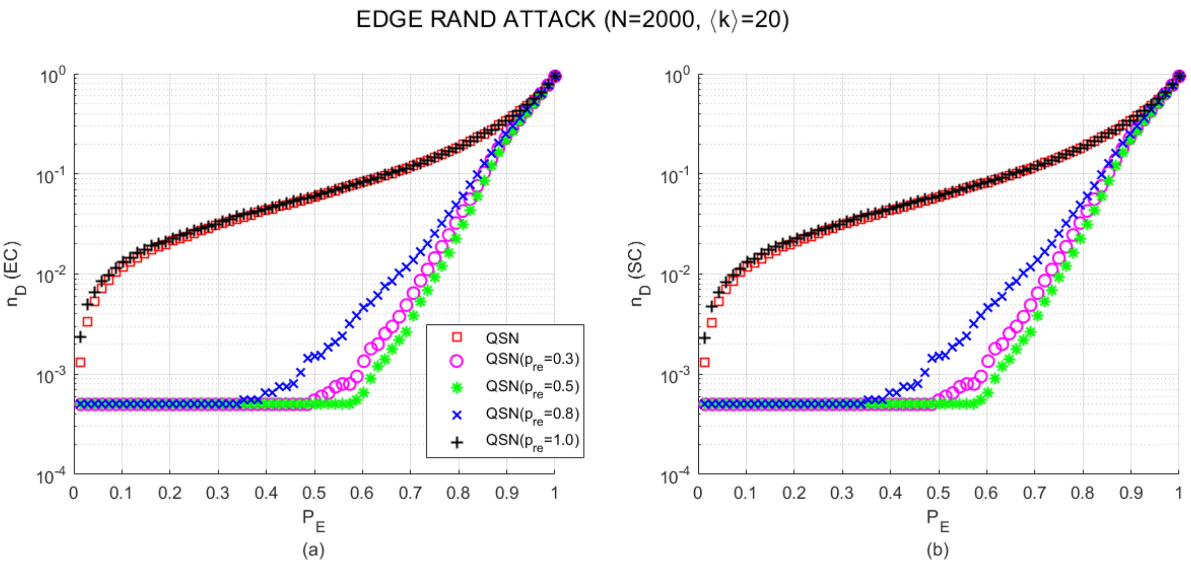
EDGE RAND ATTACK (N=2000, ⟨k⟩=20)
n_D (EC)
n_D (SC)
P_E
QSN
QSN(p_{re}=0.3)
QSN(p_{re}=0.5)
QSN(p_{re}=0.8)
QSN(p_{re}=1.0)
(a)
(b)

4.3.2 *Edge Intentional (Betweenness-based) Attack*

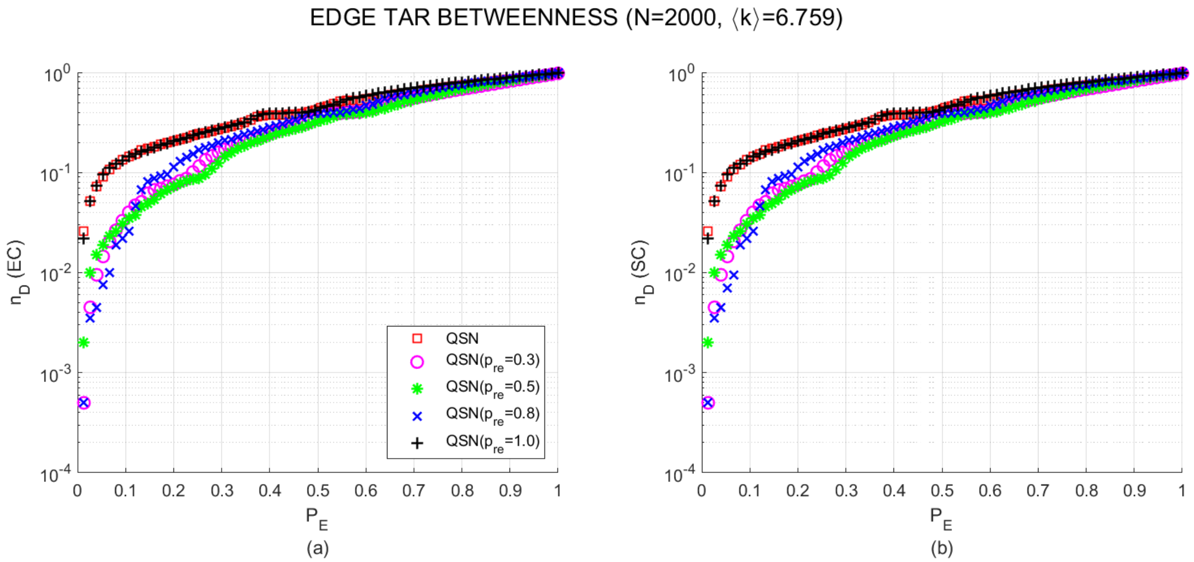

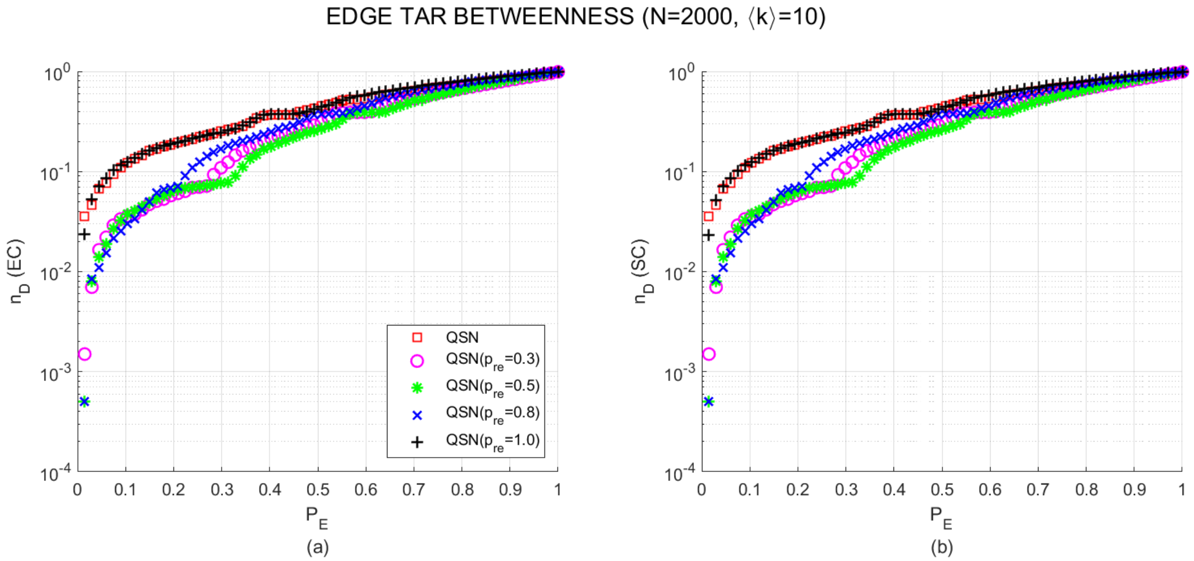

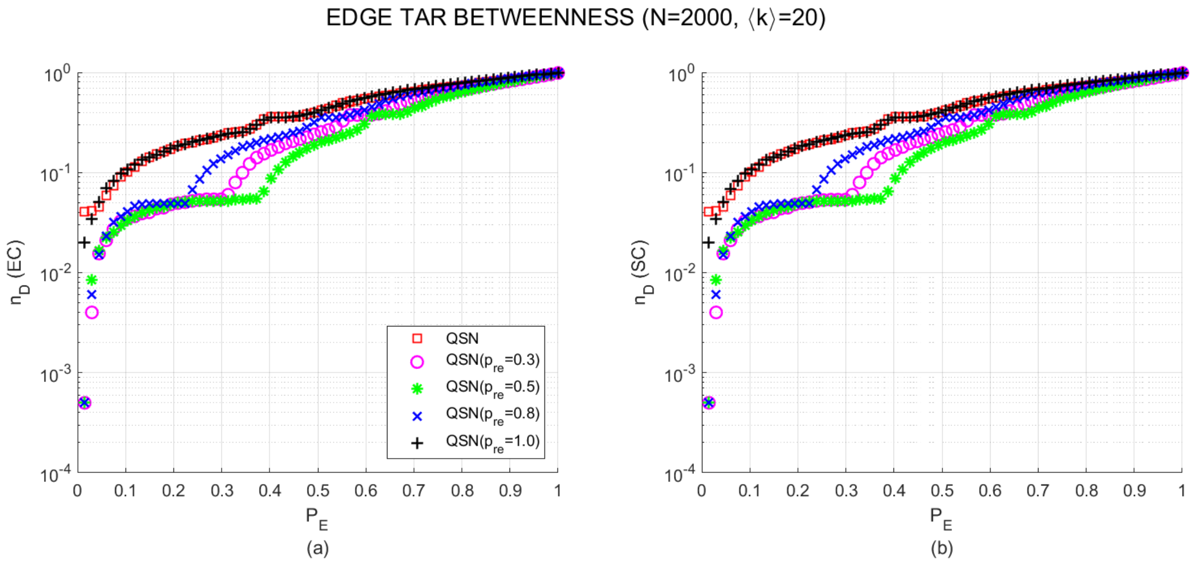

4.3.3 Edge Intentional (Degree-based) Attack

EDGE TAR DEGREE (N=2000, ⟨k⟩=6.759)

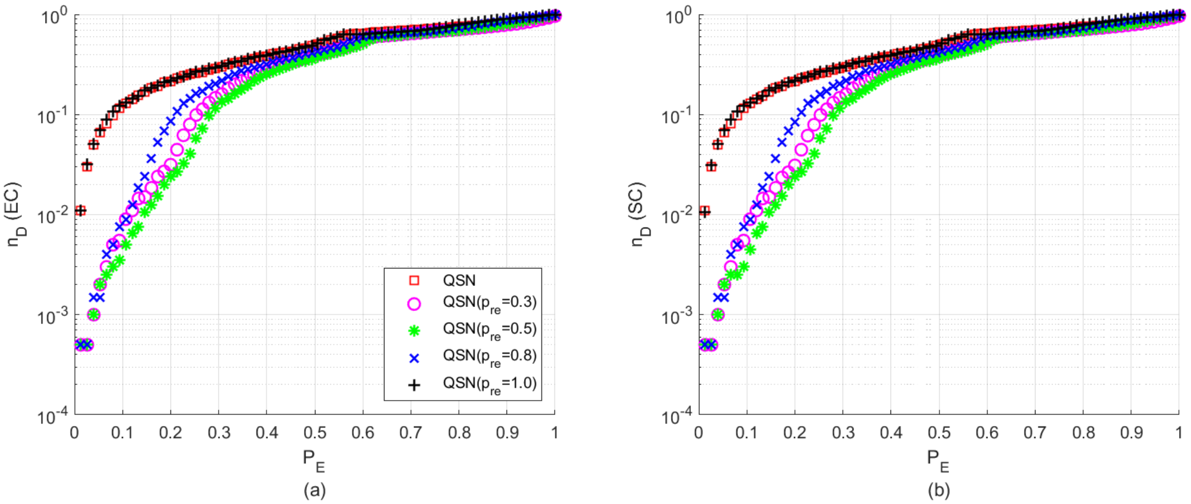

EDGE TAR DEGREE (N=2000, ⟨k⟩=10)

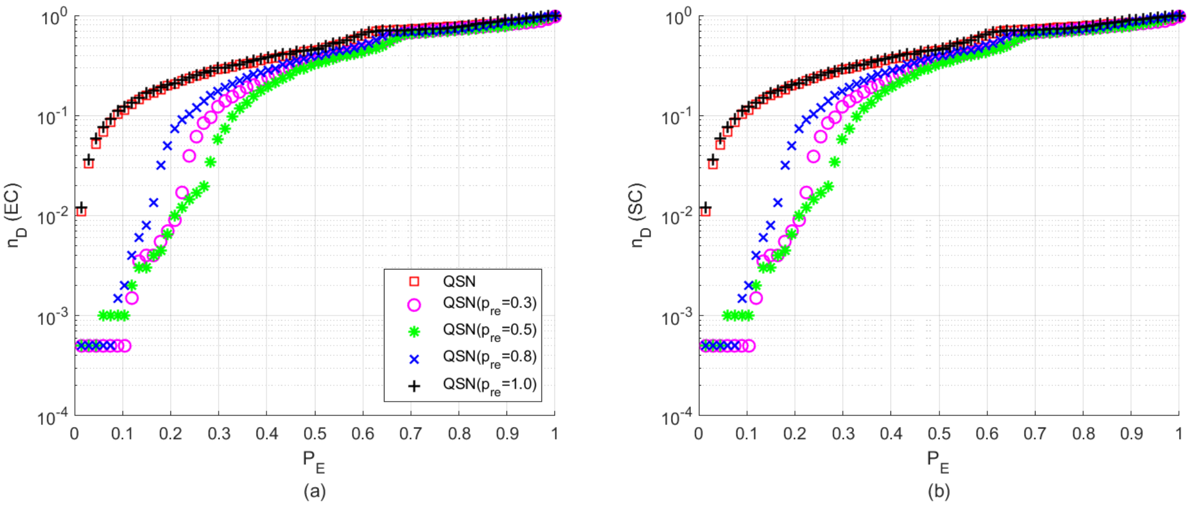

EDGE TAR DEGREE (N=2000, ⟨k⟩=20)

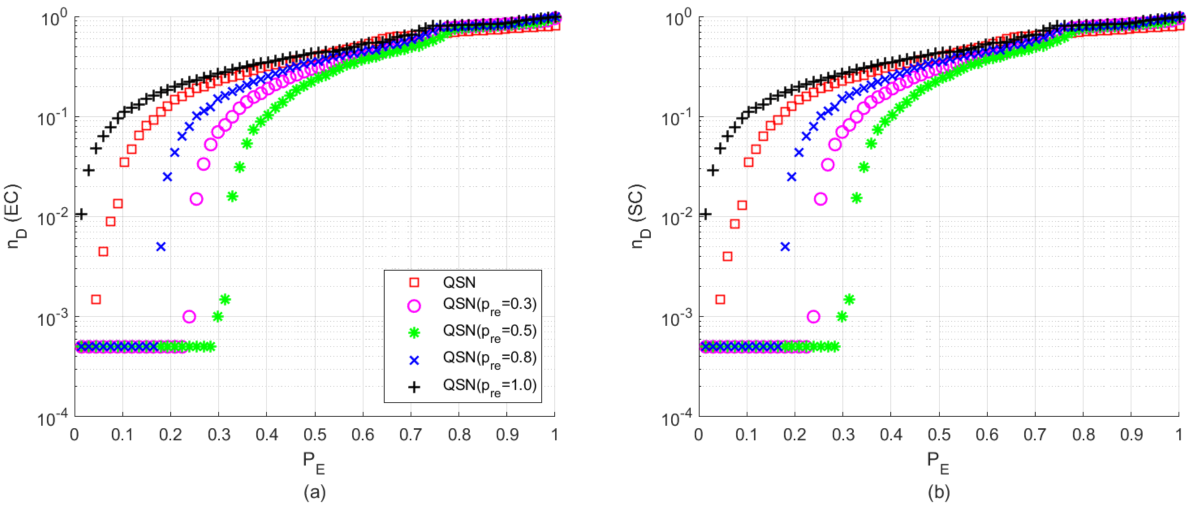

4.3.4 Node Random Attack

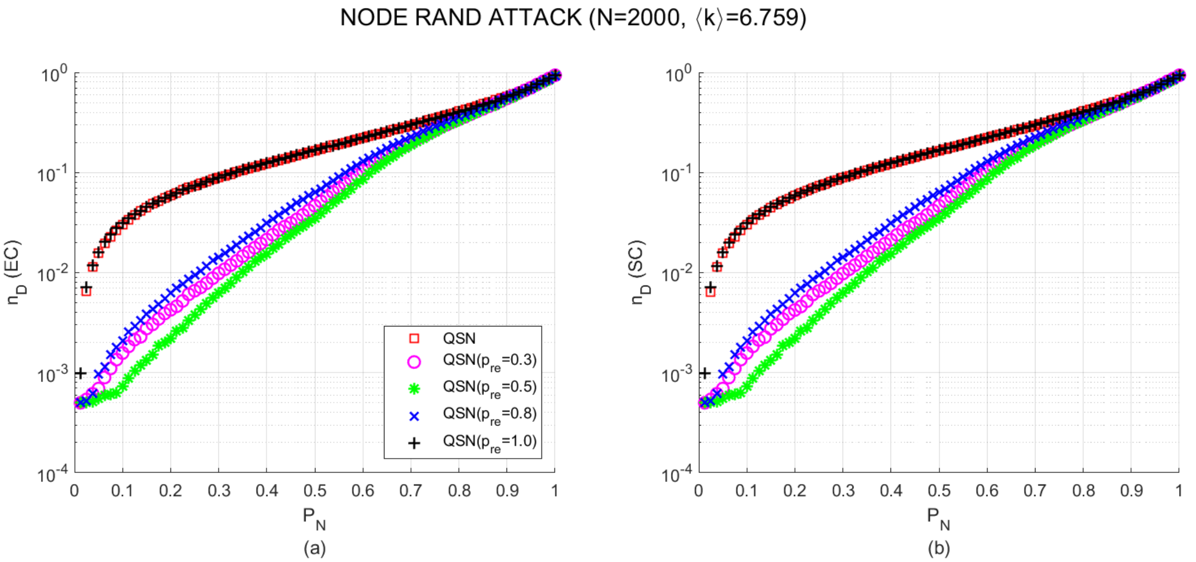

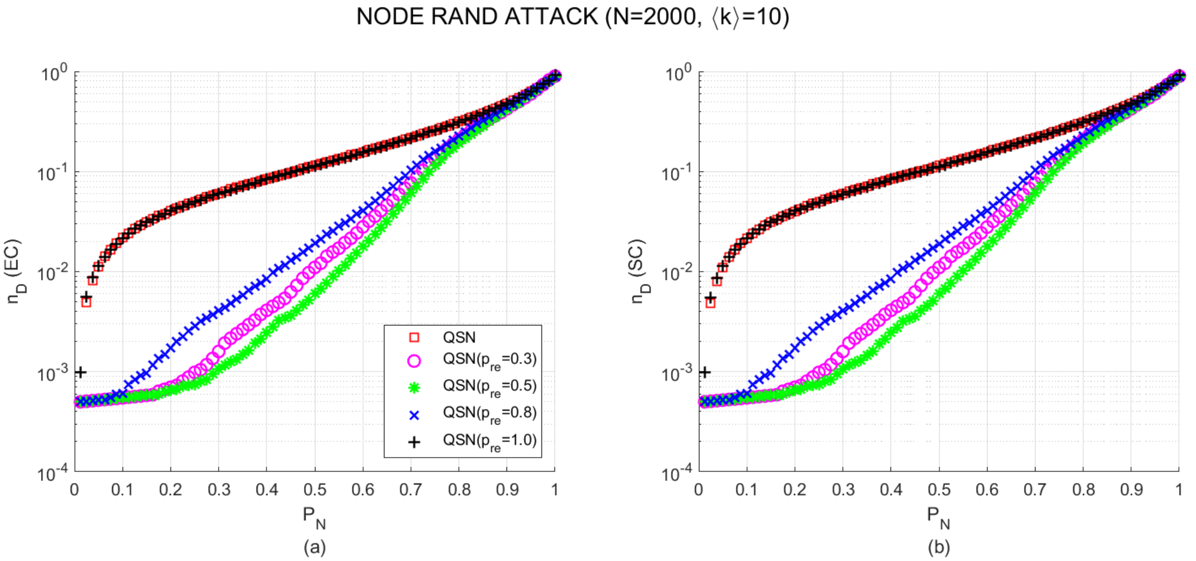

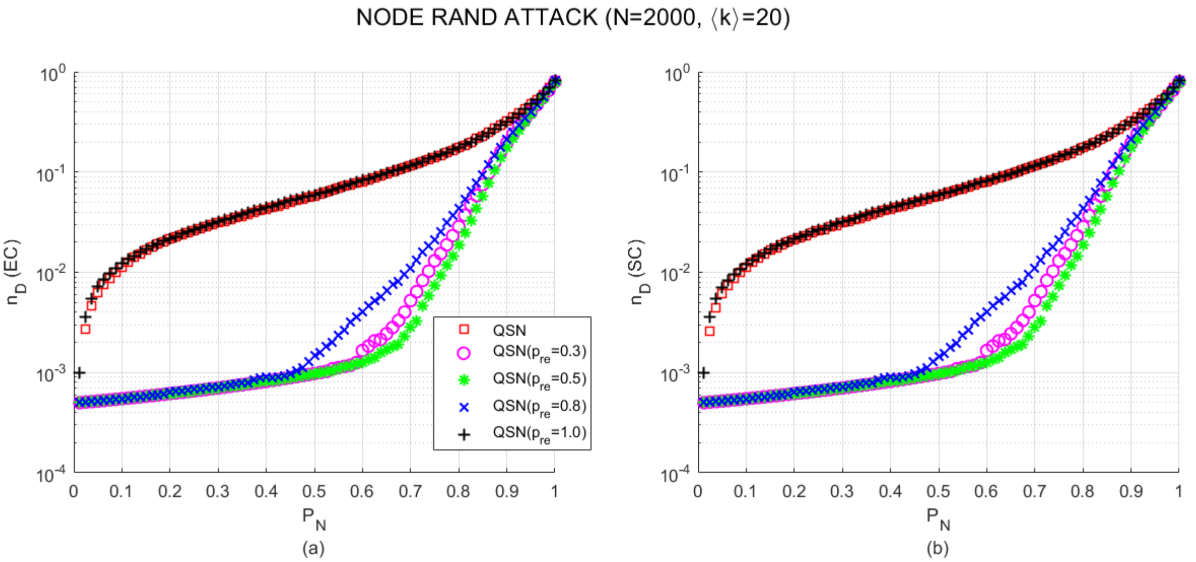

4.3.5 Node Intentional (Betweenness-based) Attack

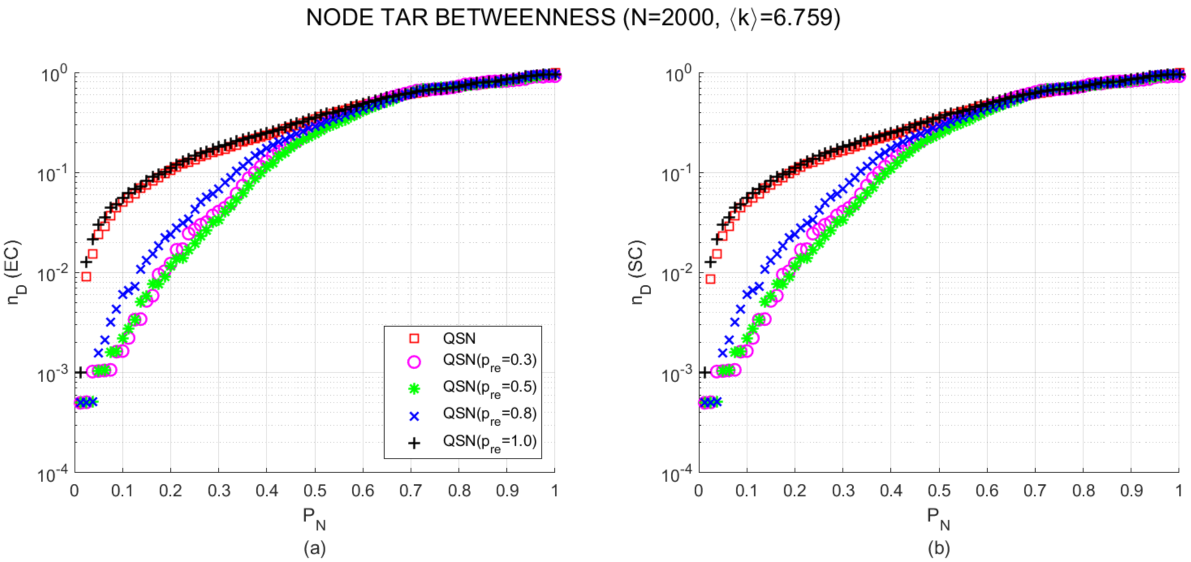

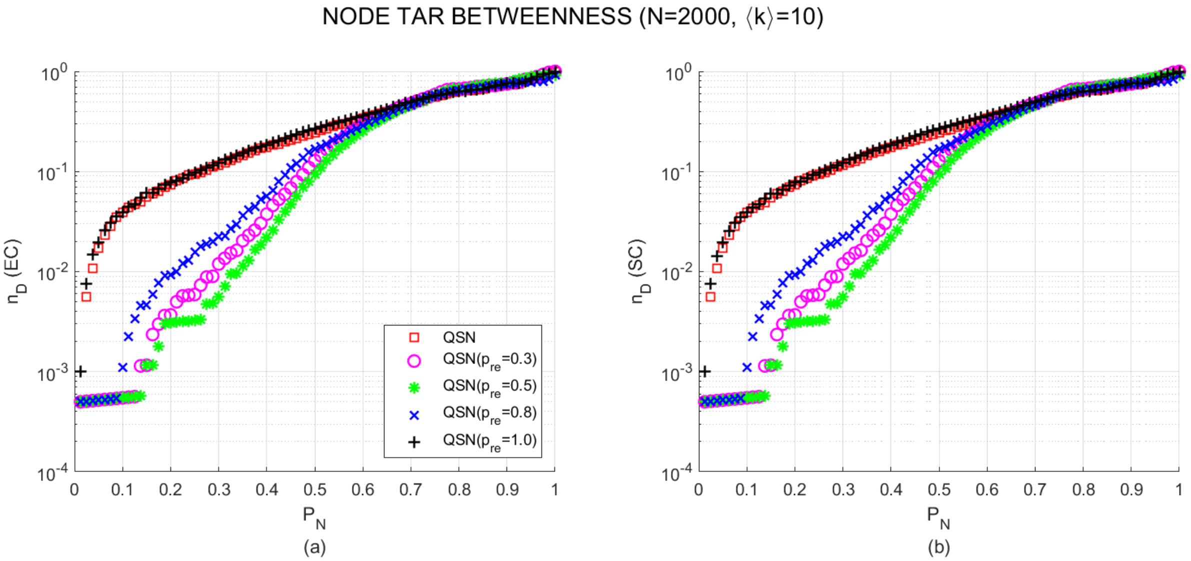

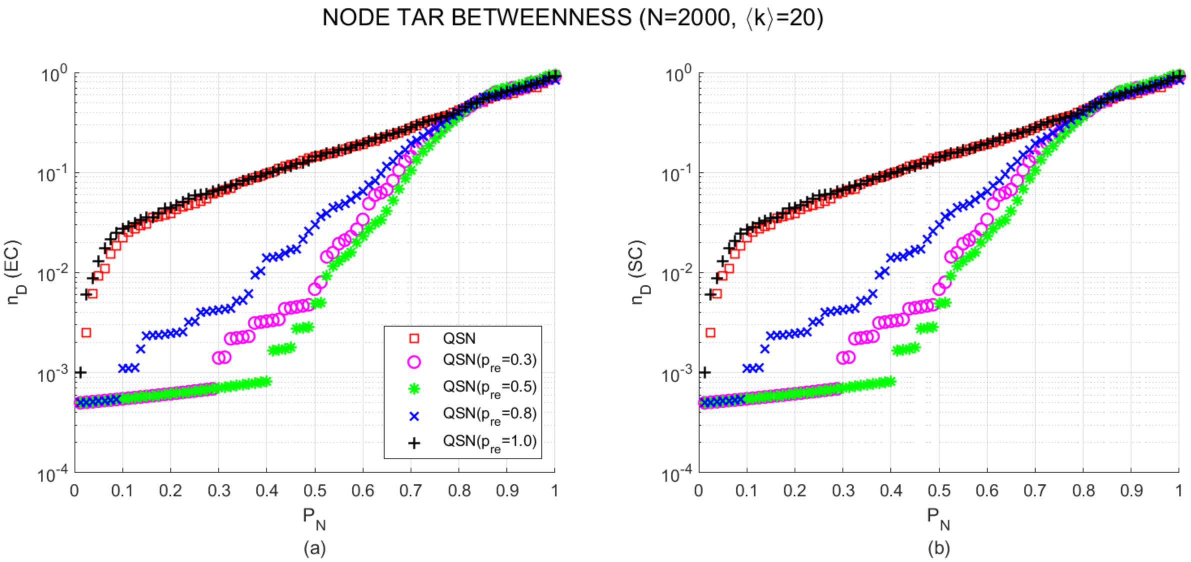

4.3.6 Node Intentional (Degree-based) Attack

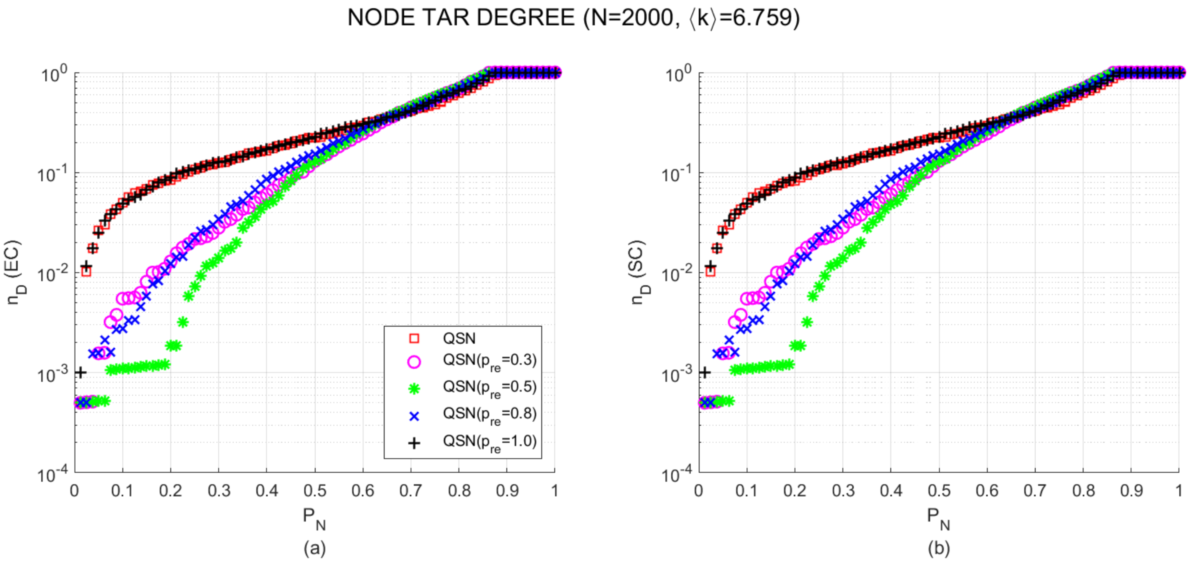

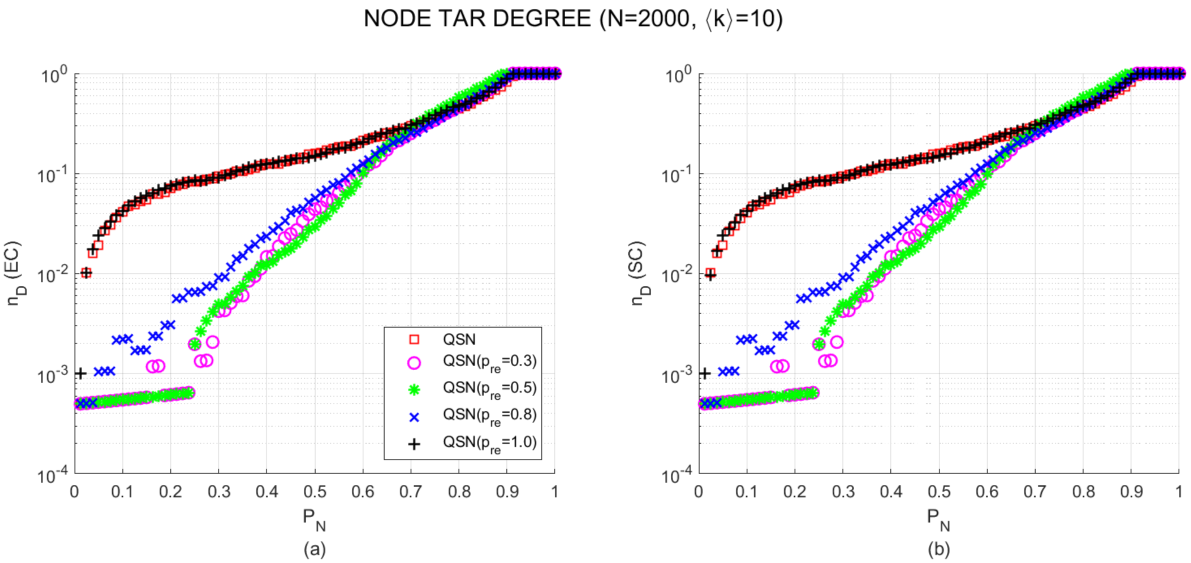

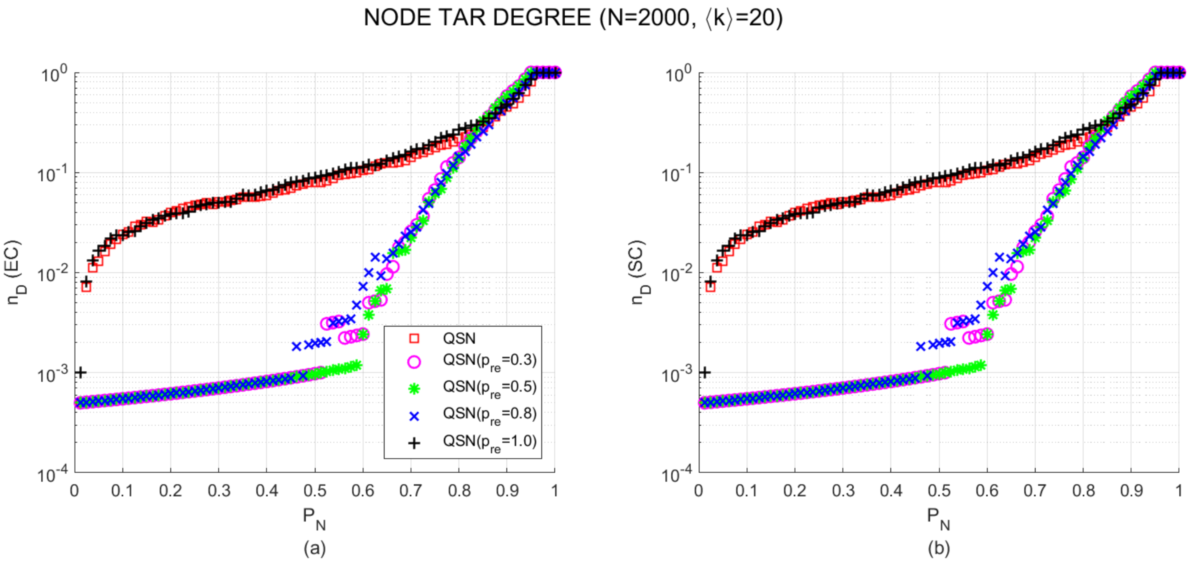

5 QSN with $p_{re} = 0.5$ vs. Other Network Topologies Comparison Curves

5.1 Network Size N=500

5.1.1 Edge Random Attack

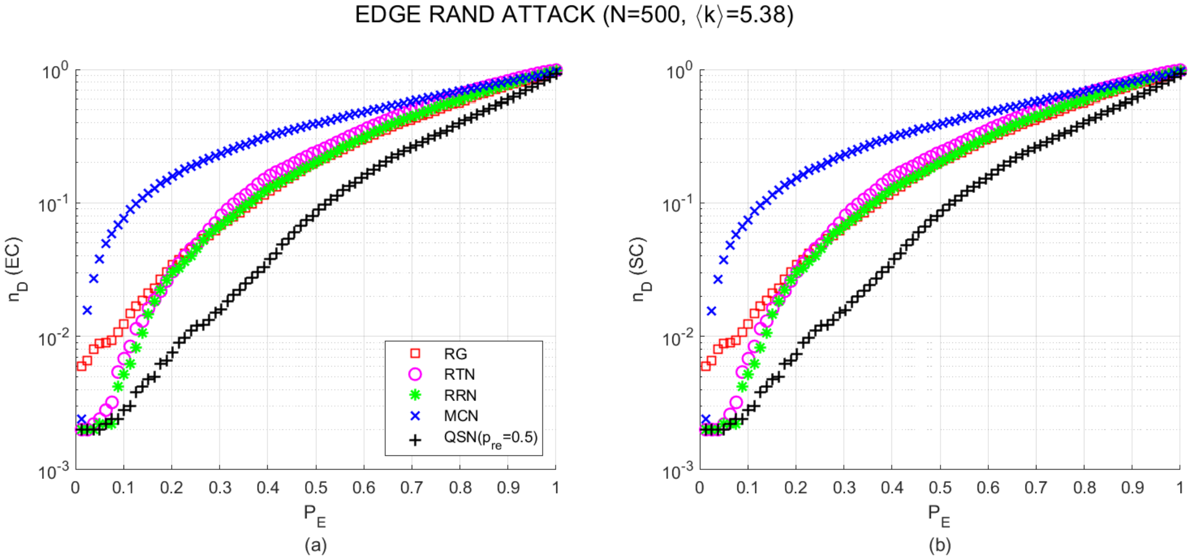

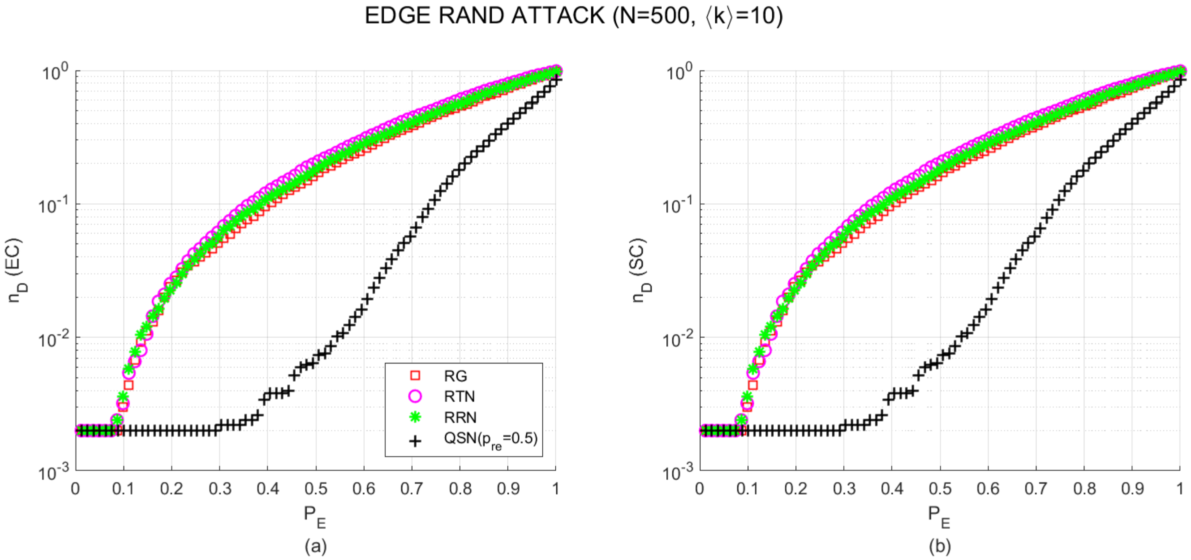

5.1.2 Edge Intentional (Betweenness-based) Attack

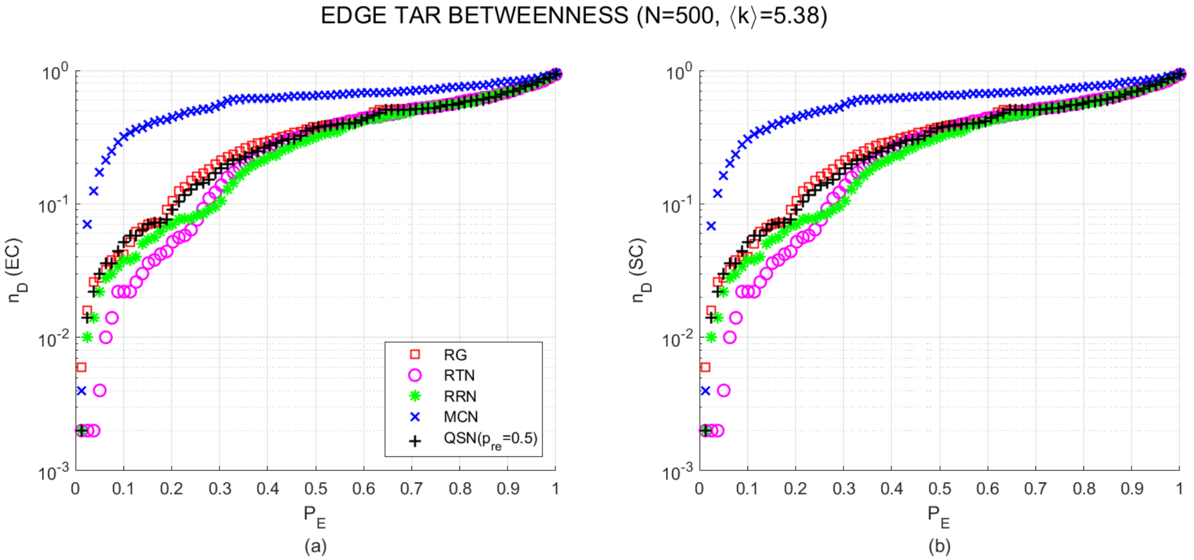

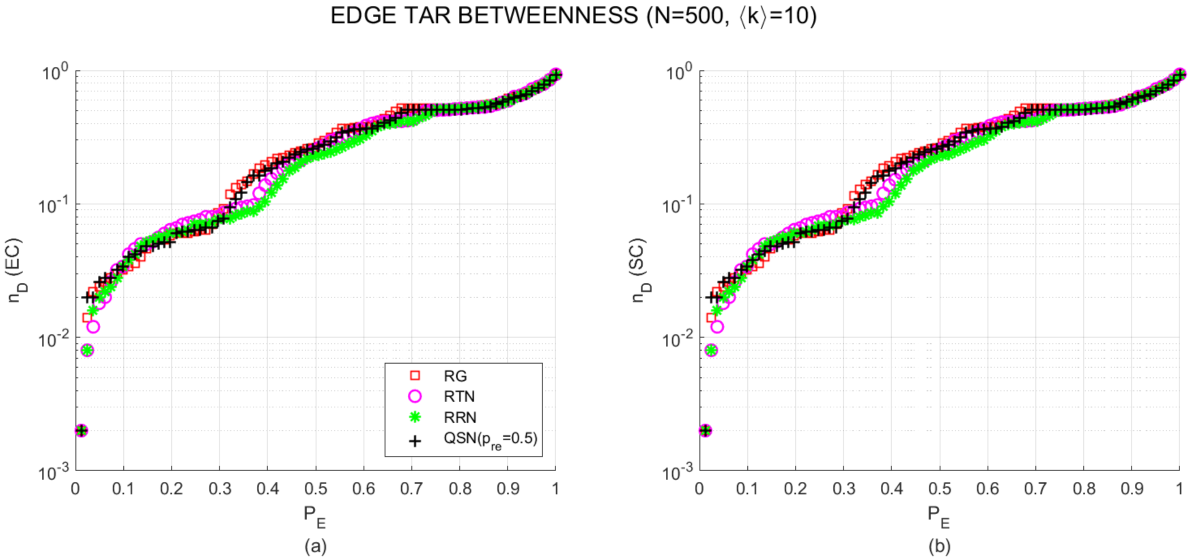

5.1.3 Edge Intentional (Degree-based) Attack

EDGE TAR DEGREE (N=500, ⟨k⟩=5.38)

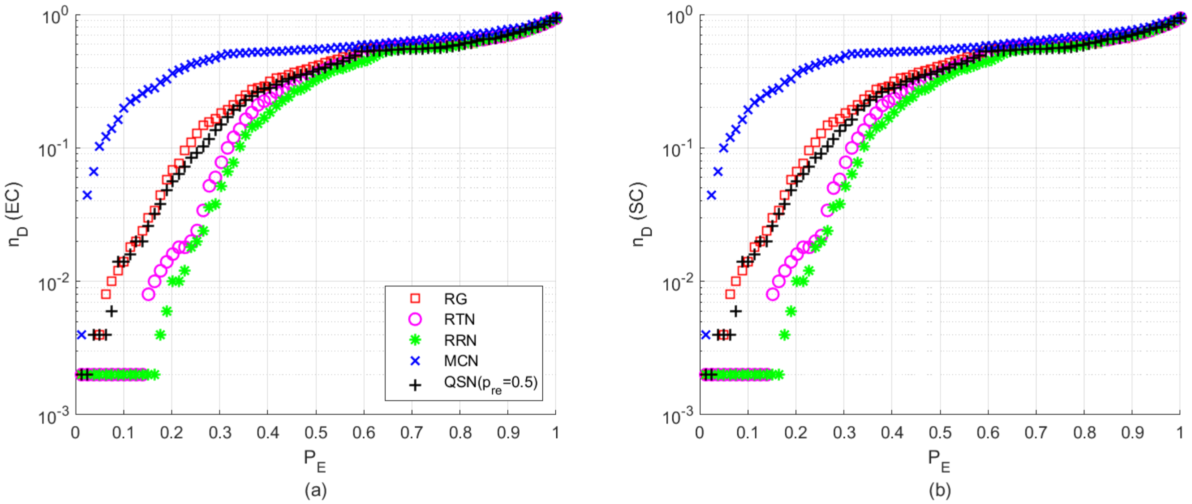

EDGE TAR DEGREE (N=500, ⟨k⟩=10)

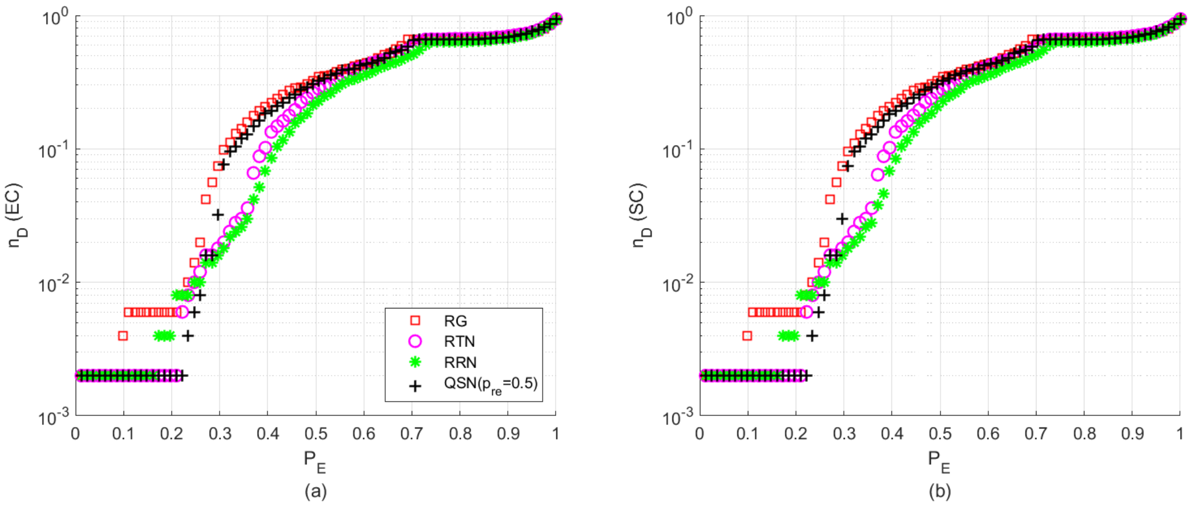

5.1.4 Node Random Attack

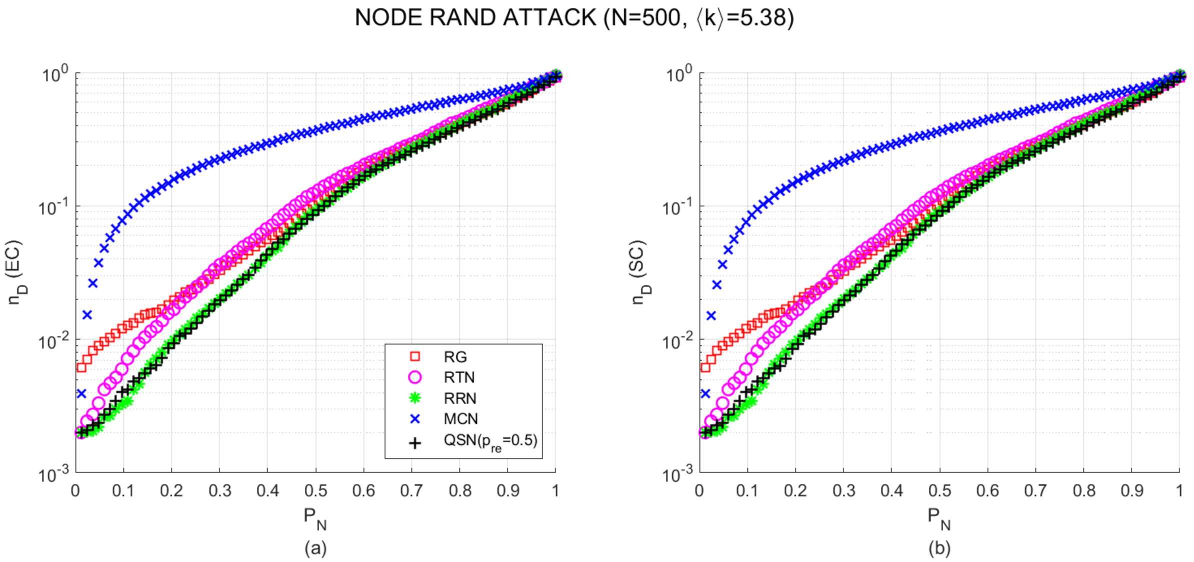

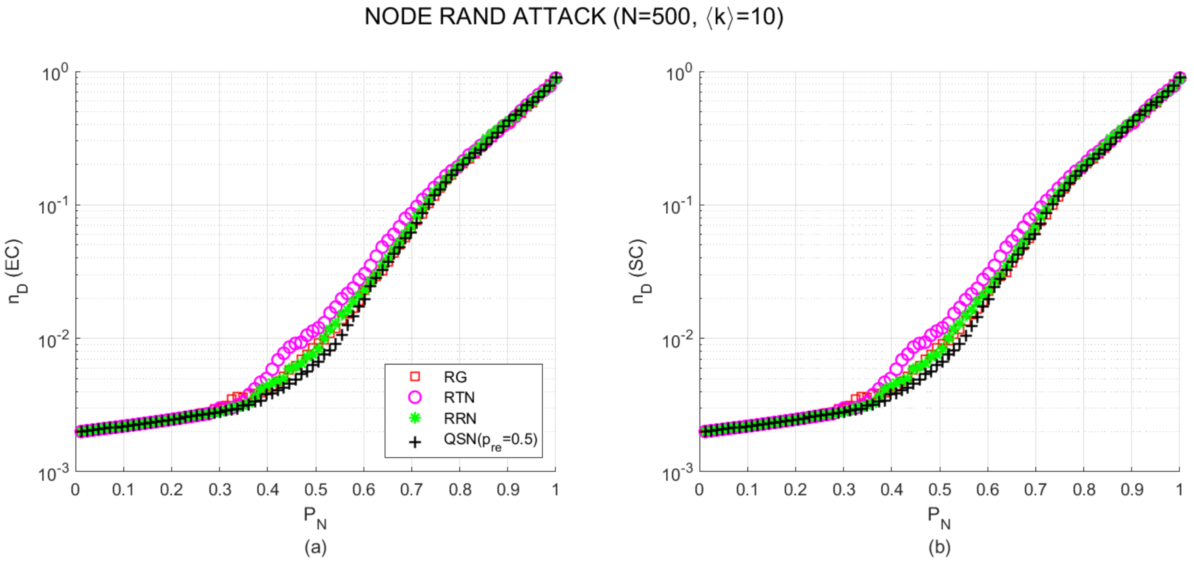

5.1.5 *Node Intentional (Betweenness-based) Attack*

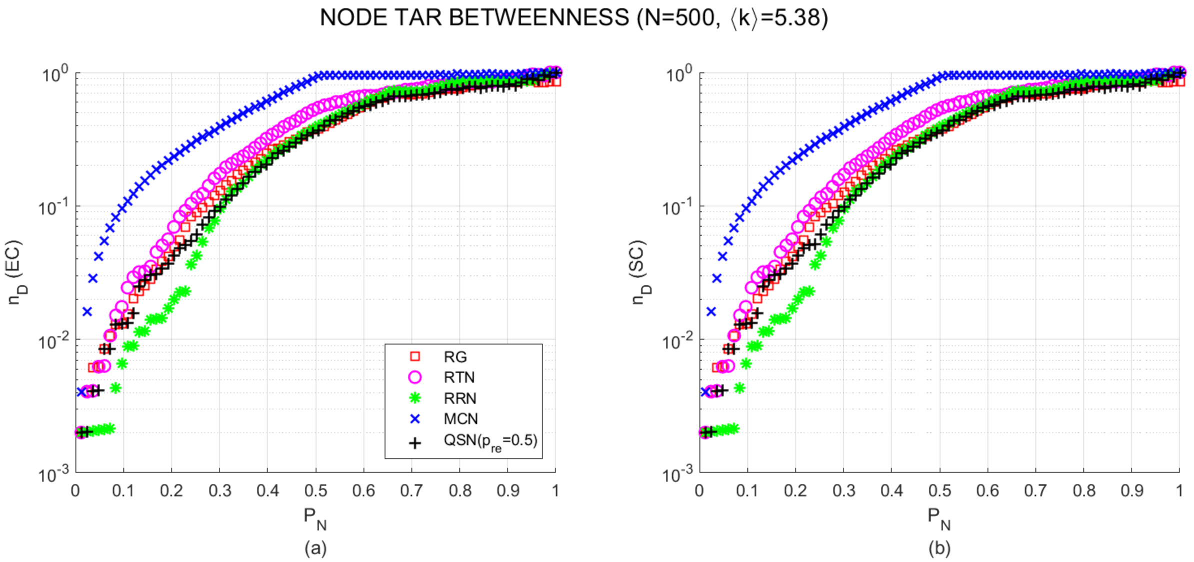

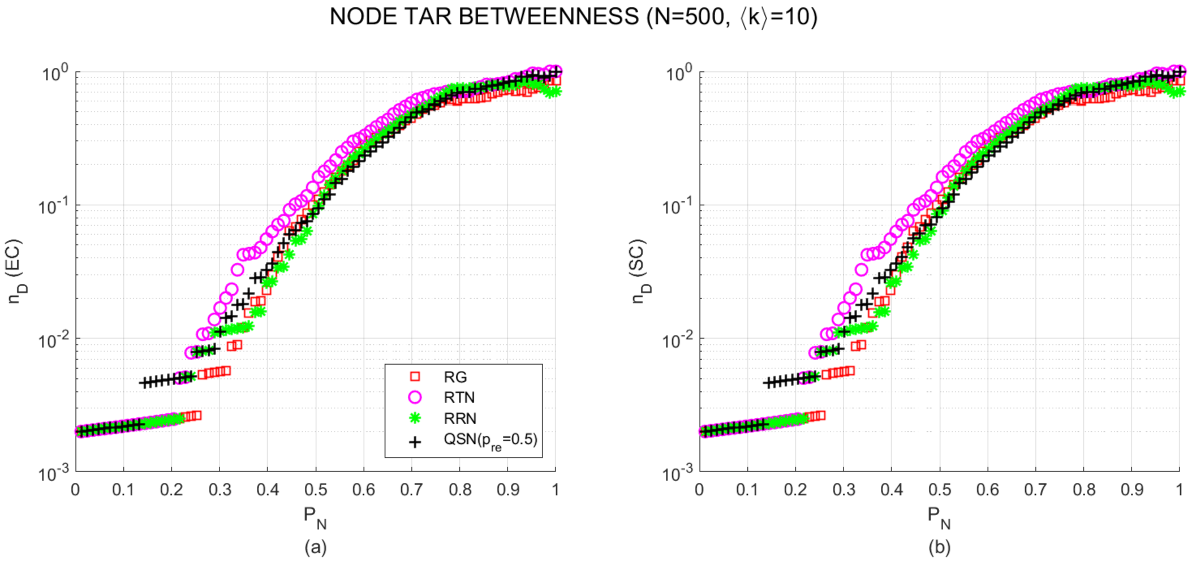

5.1.6 Node Intentional (Degree-based) Attack

NODE TAR DEGREE (N=500, ⟨k⟩=5.38)

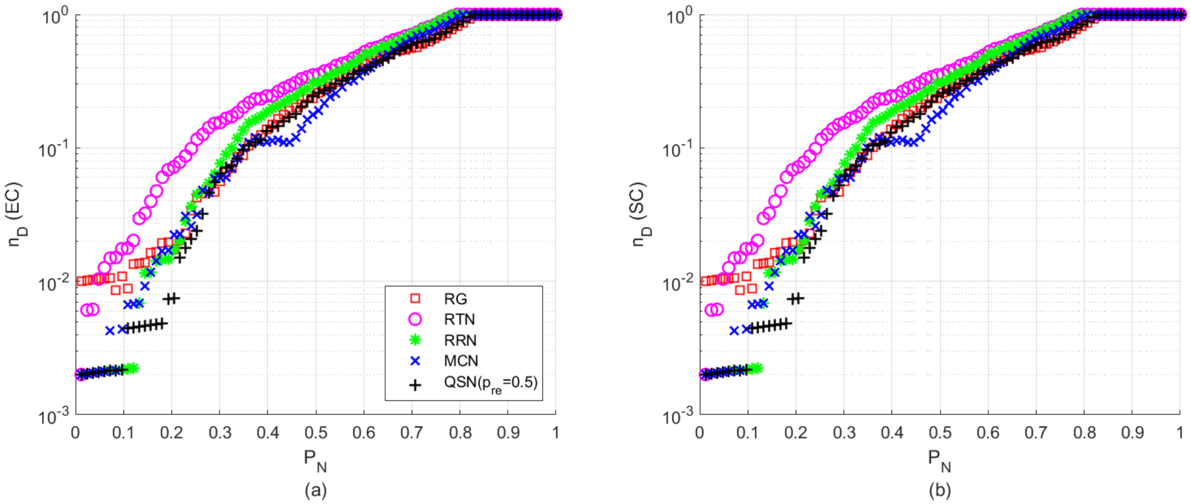

NODE TAR DEGREE (N=500, ⟨k⟩=10)

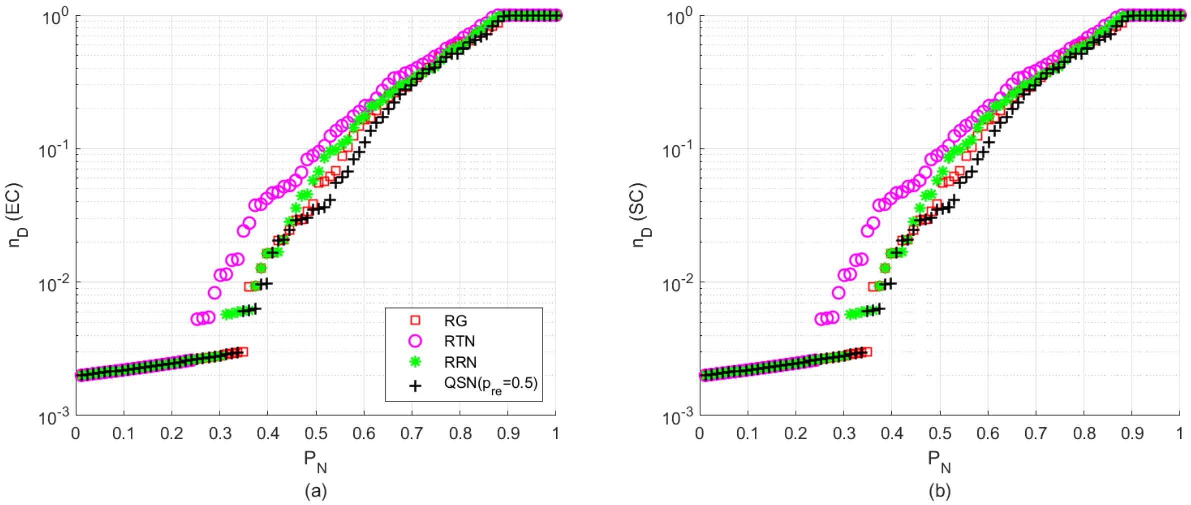

5.2 Network Size N=1000

5.2.1 Edge Random Attack

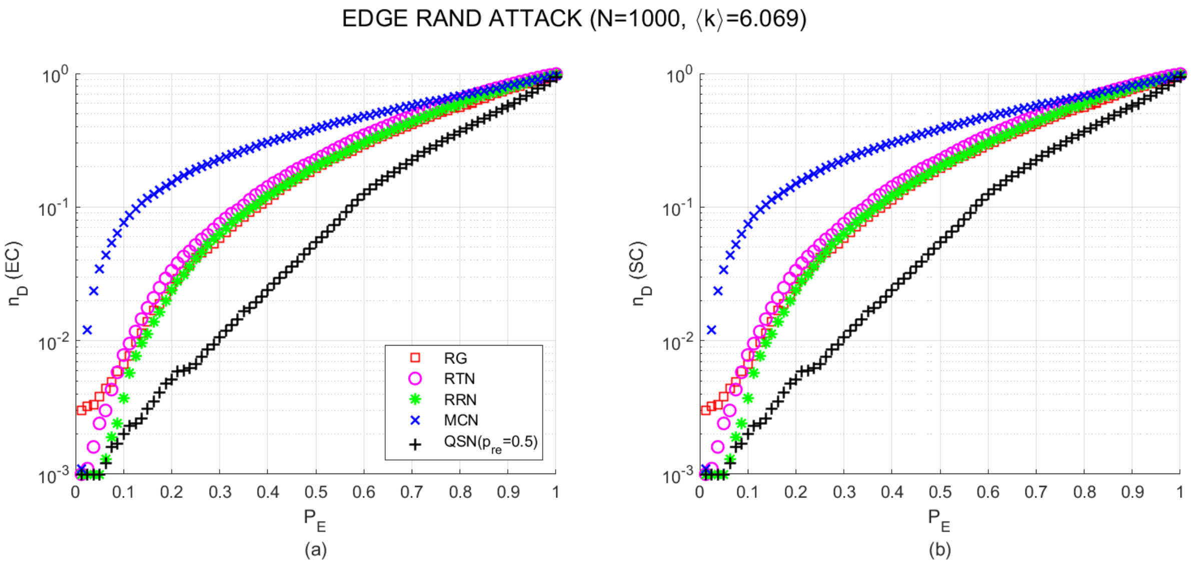

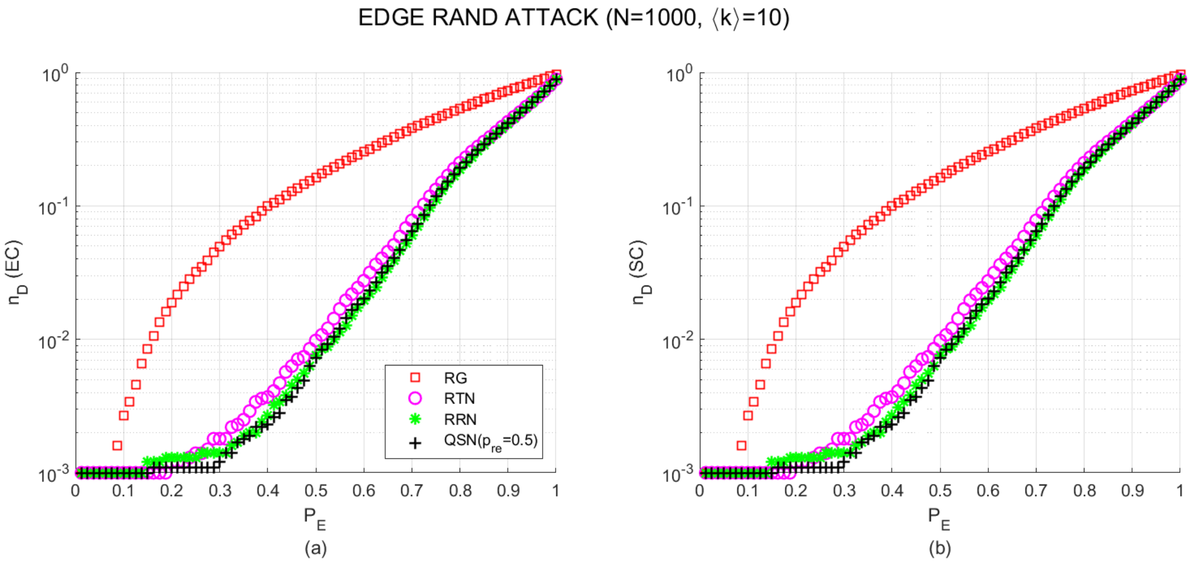

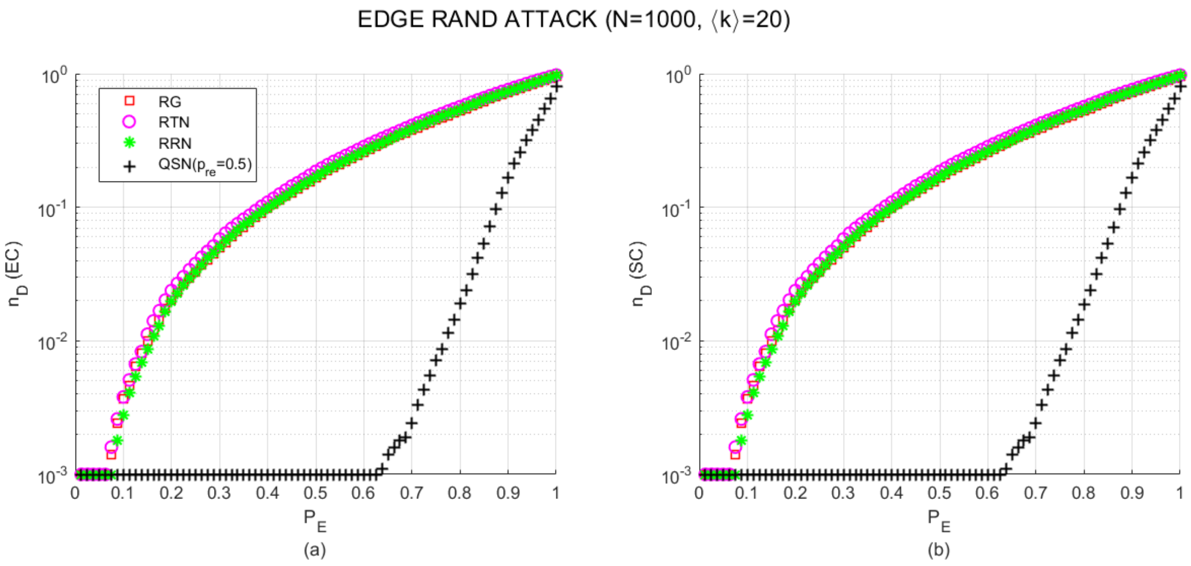
EDGE RAND ATTACK (N=1000, ⟨k⟩=20)
RG
RTN
RRN
QSN(p_re=0.5)
n_D (EC)
n_D (SC)
P_E
(a)
(b)

5.2.2 *Edge Intentional (Betweenness-based) Attack*

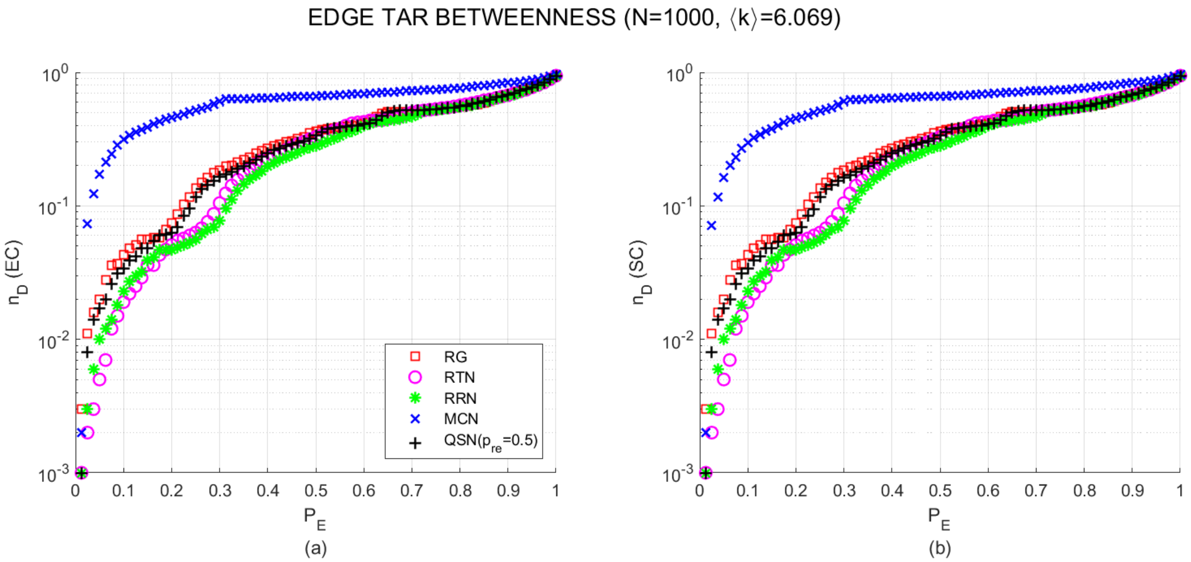

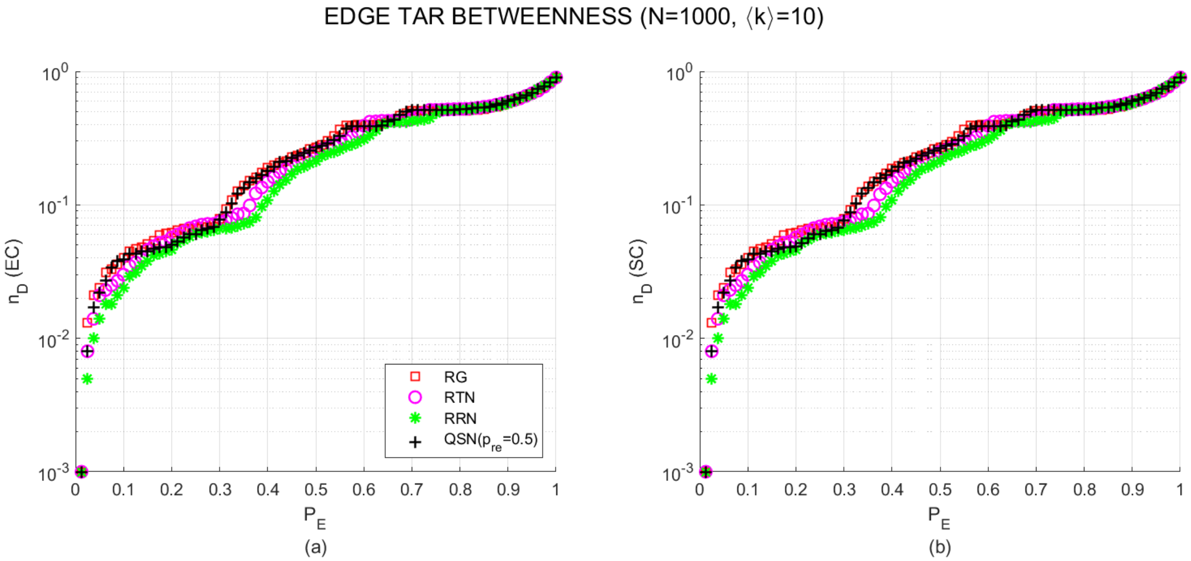

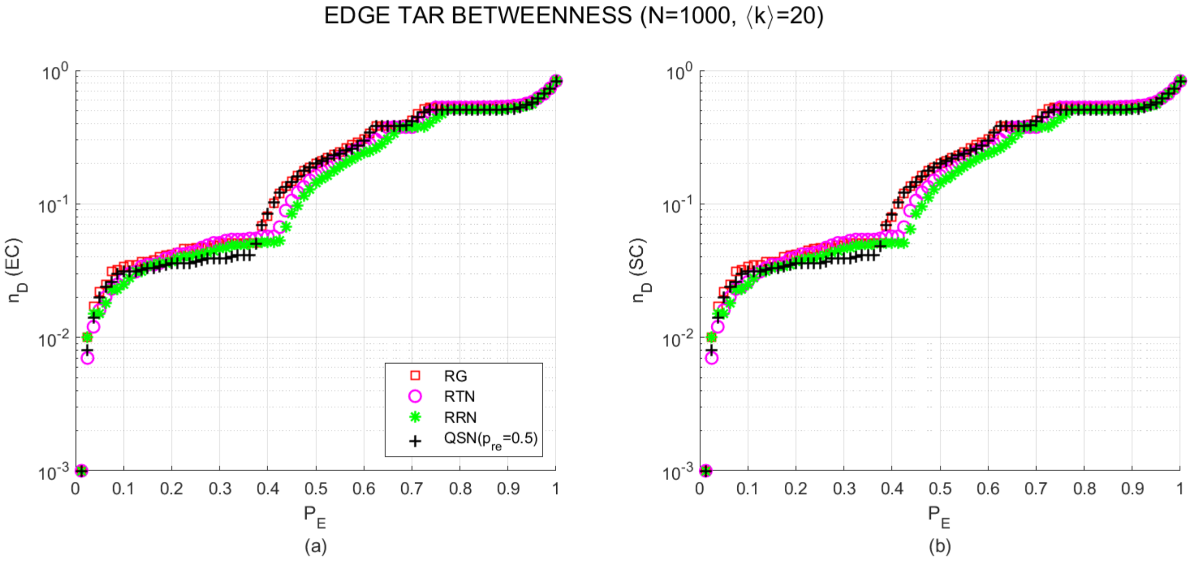

5.2.3 Edge Intentional (Degree-based) Attack

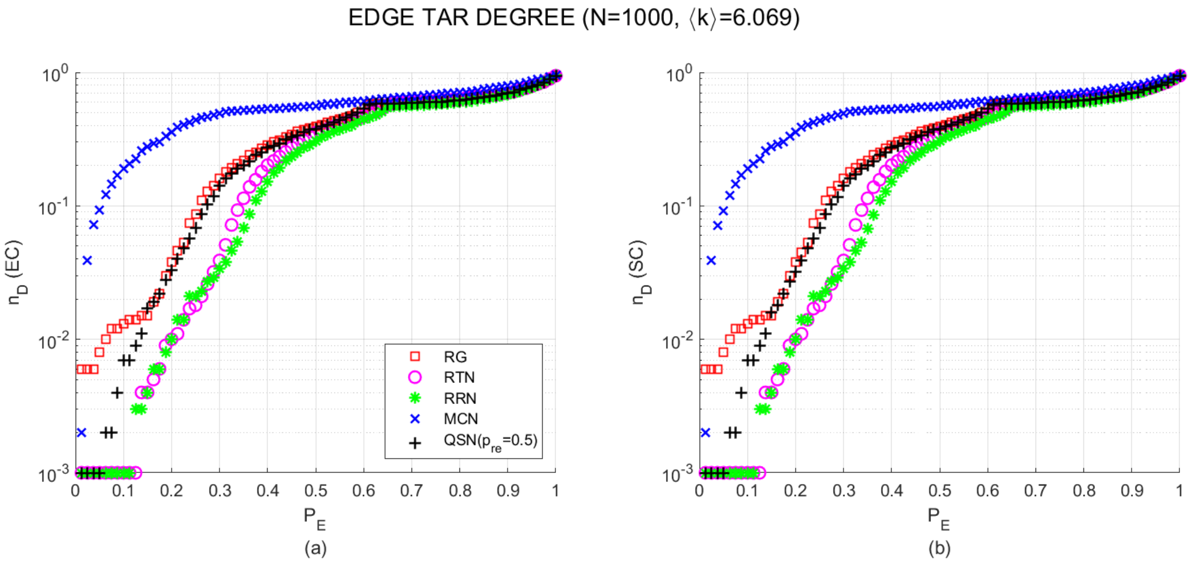

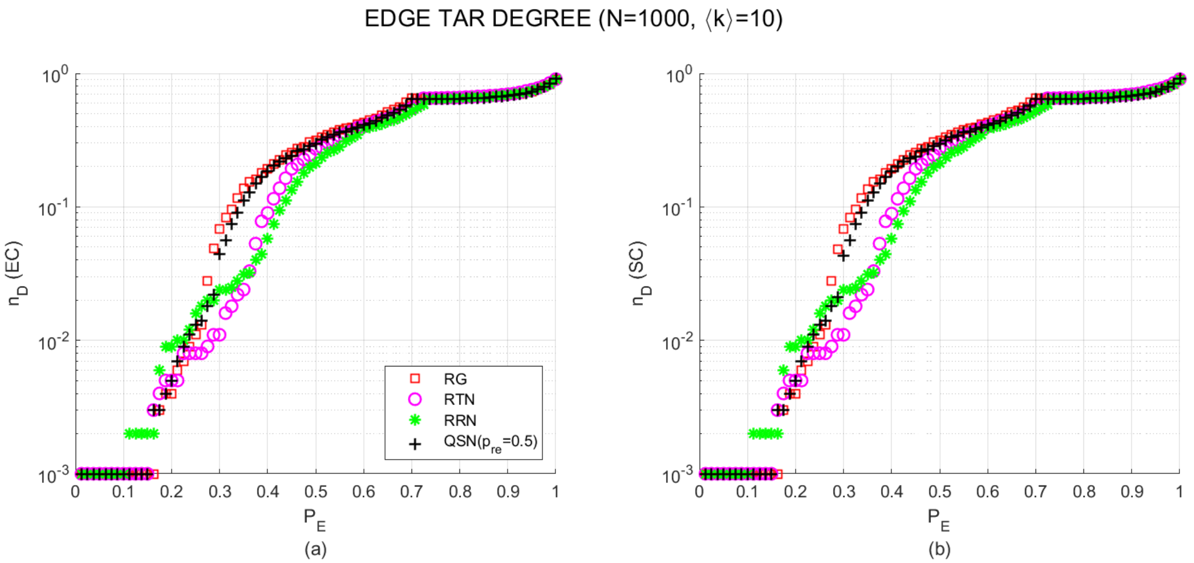

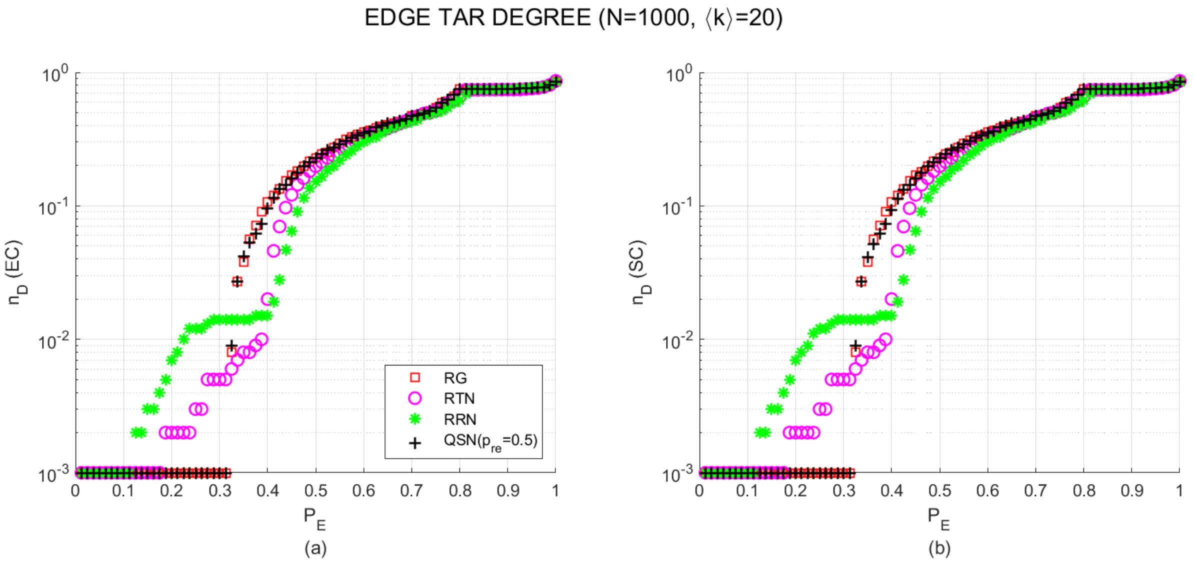

5.2.4 Node Random Attack

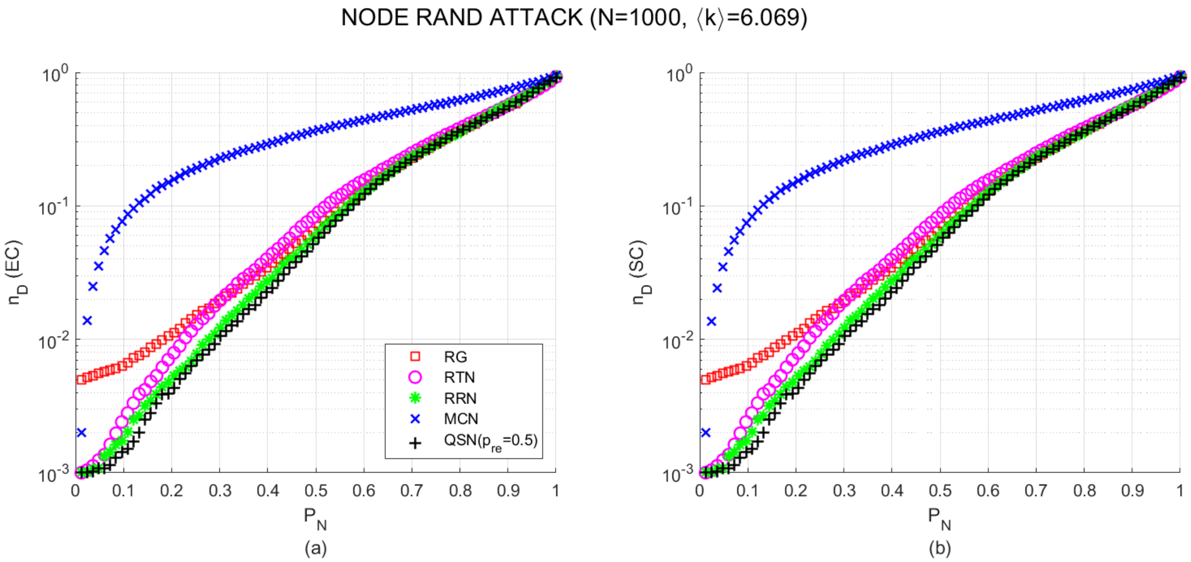

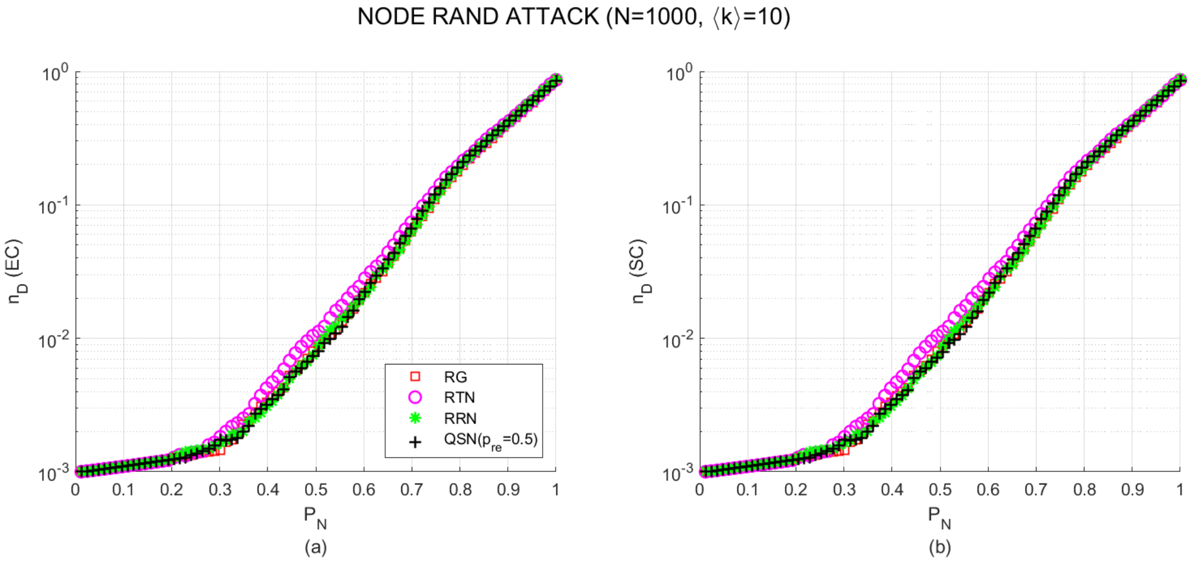

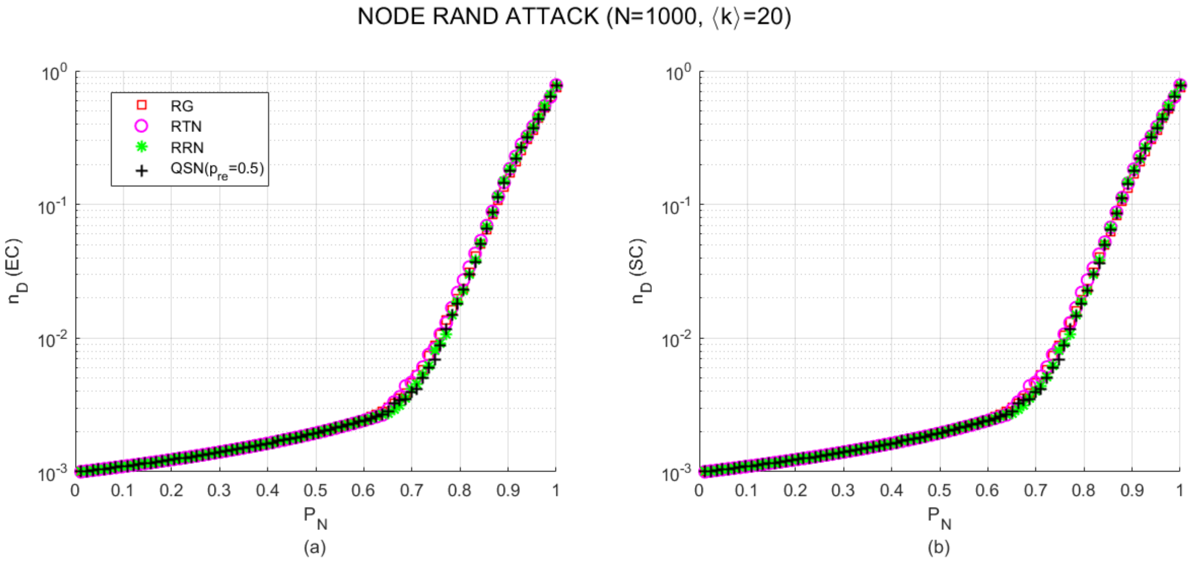

5.2.5 Node Intentional (Betweenness-based) Attack

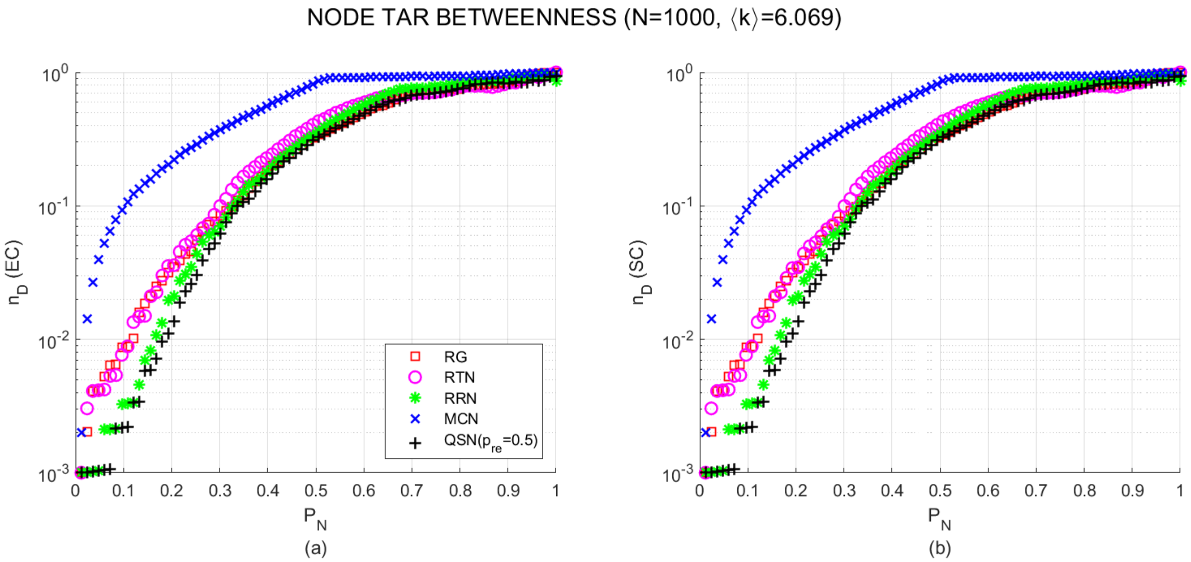

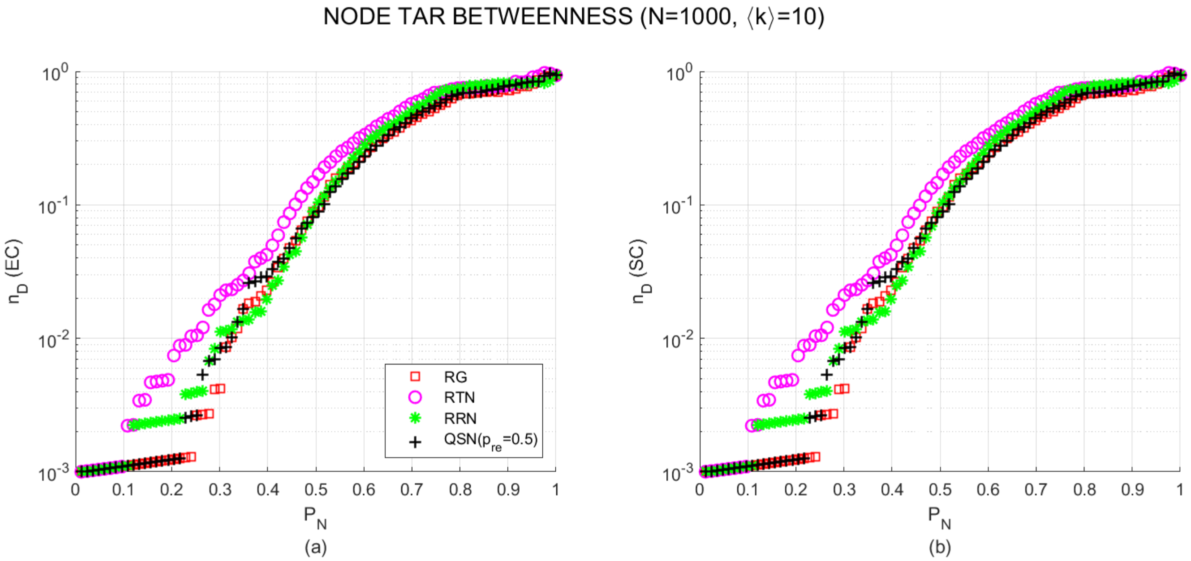

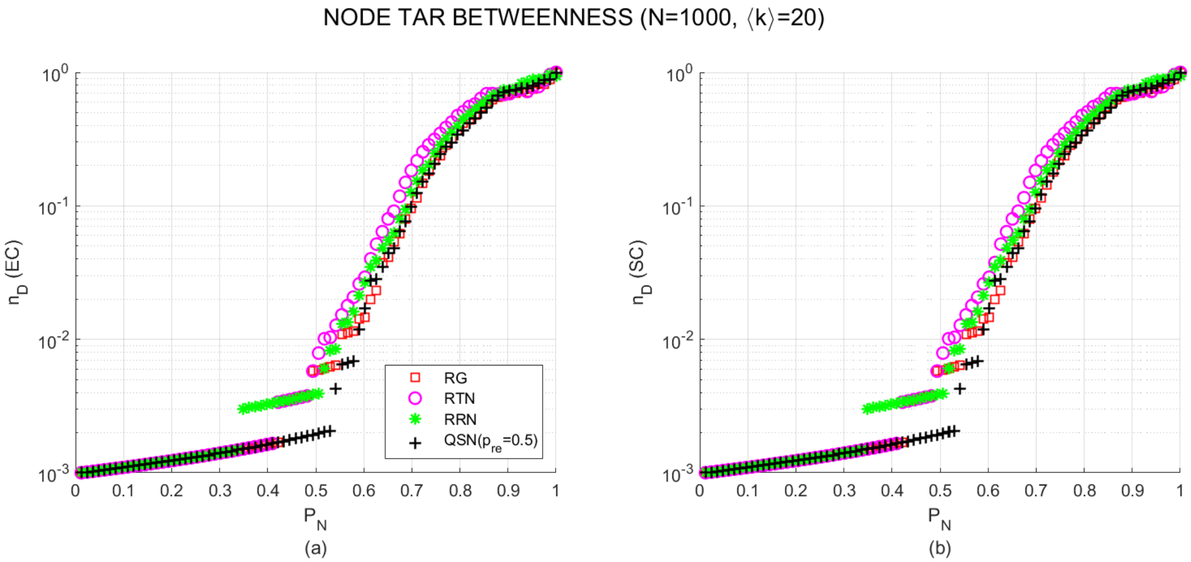

5.2.6 Node Intentional (Degree-based) Attack

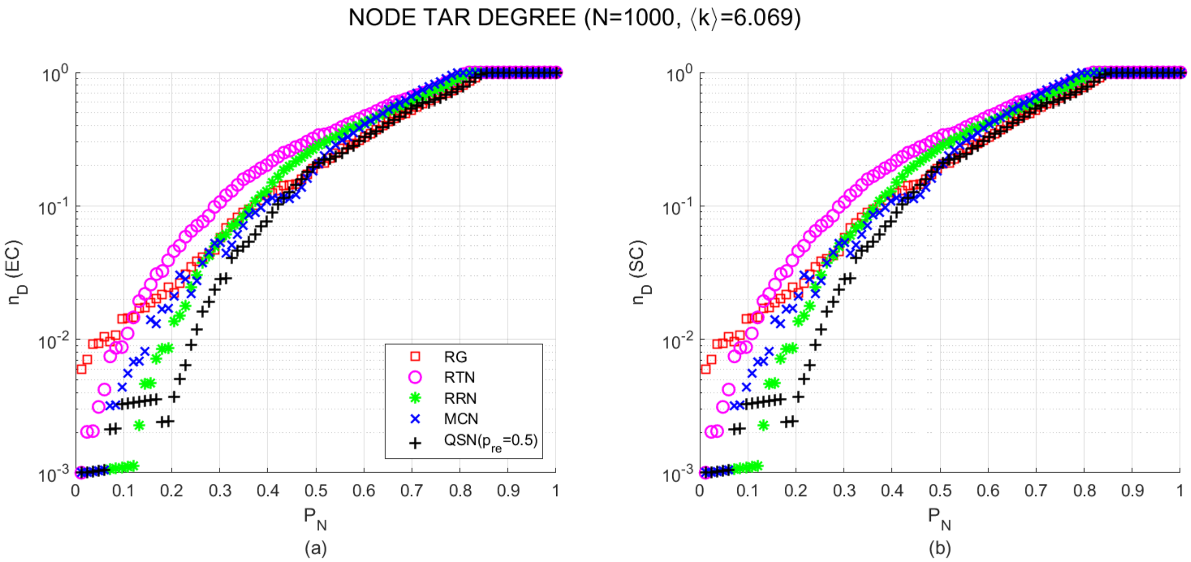

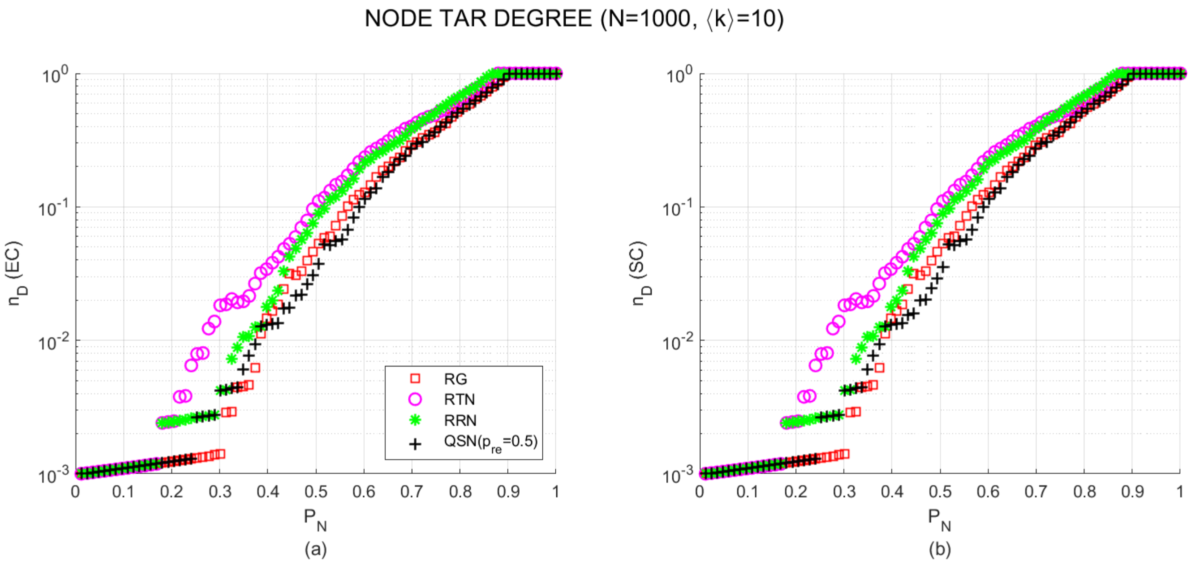

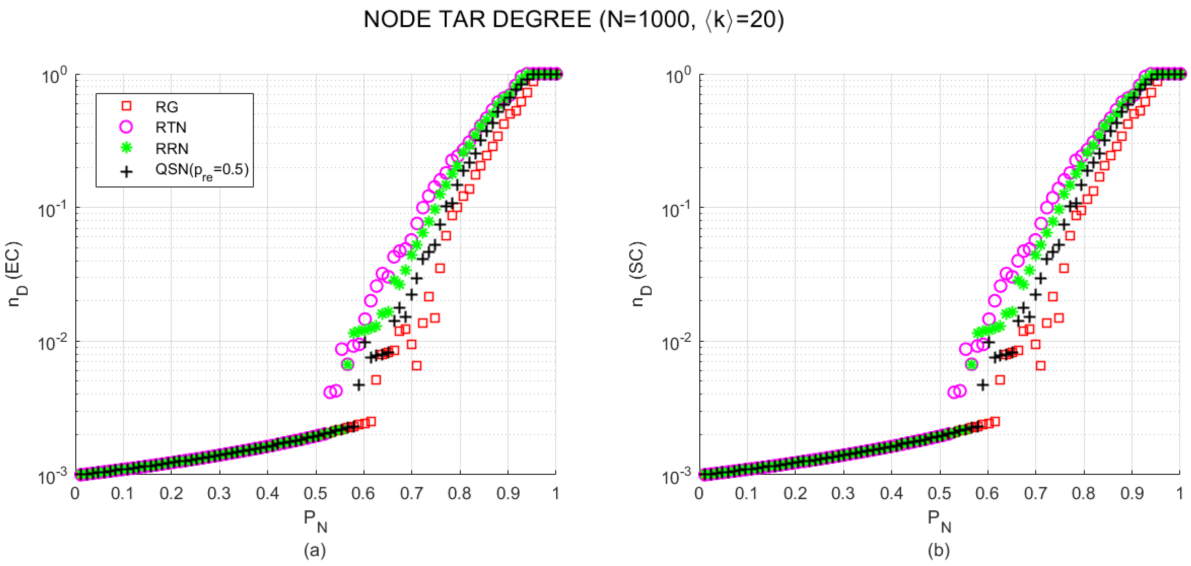

5.3 Network Size N=2000

5.3.1 Edge Random Attack

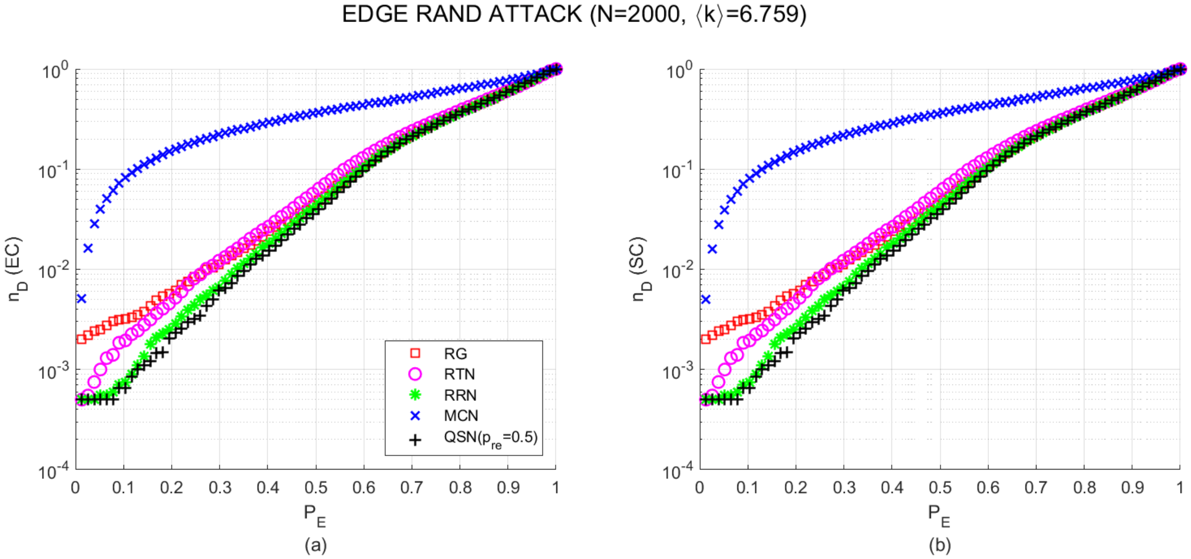

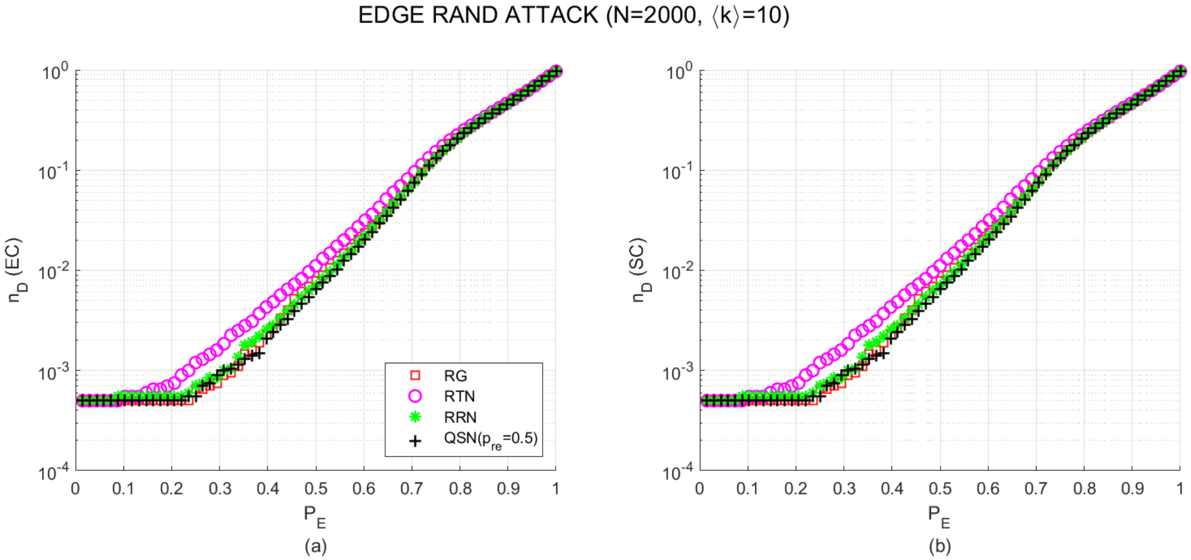

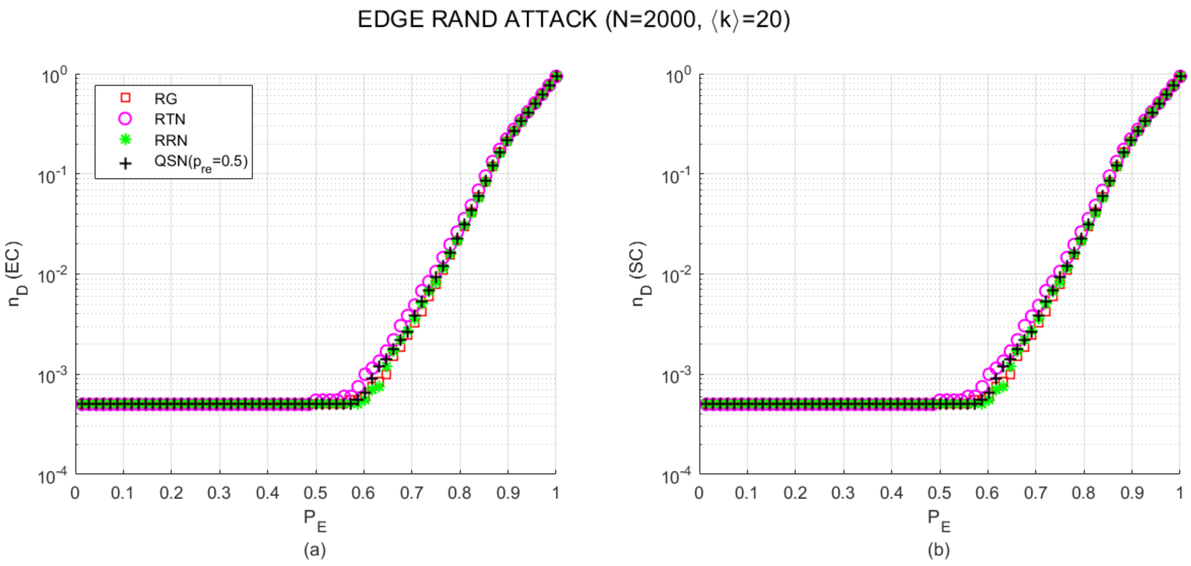

EDGE RAND ATTACK (N=2000, ⟨k⟩=20)
RG
RTN
RRN
QSN(p_re=0.5)
n_D (EC)
n_D (SC)
P_E

5.3.2 *Edge Intentional (Betweenness-based) Attack*

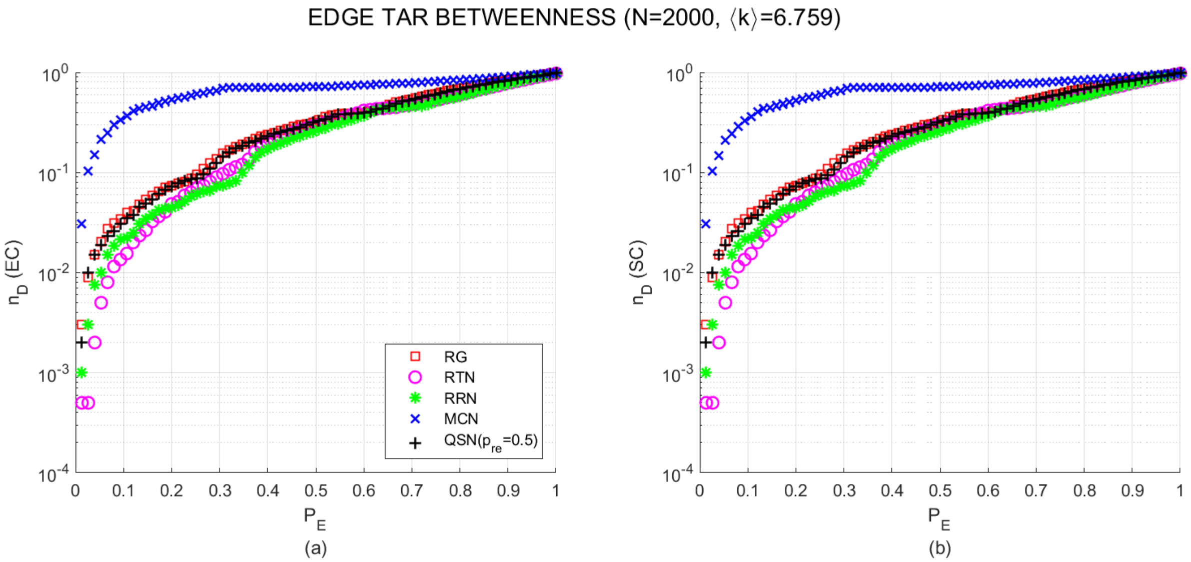

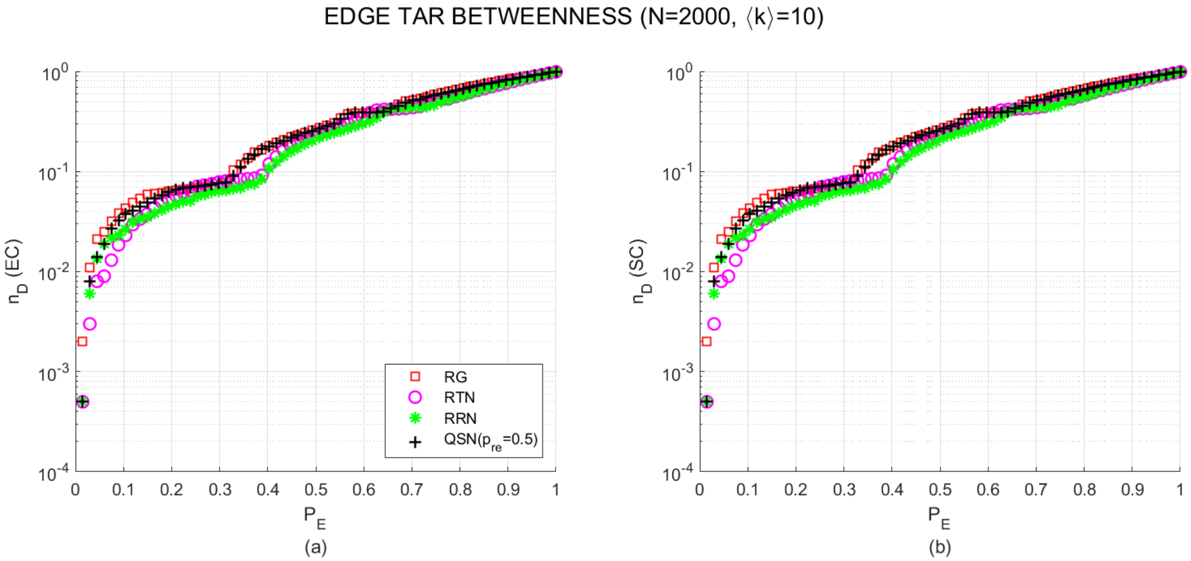

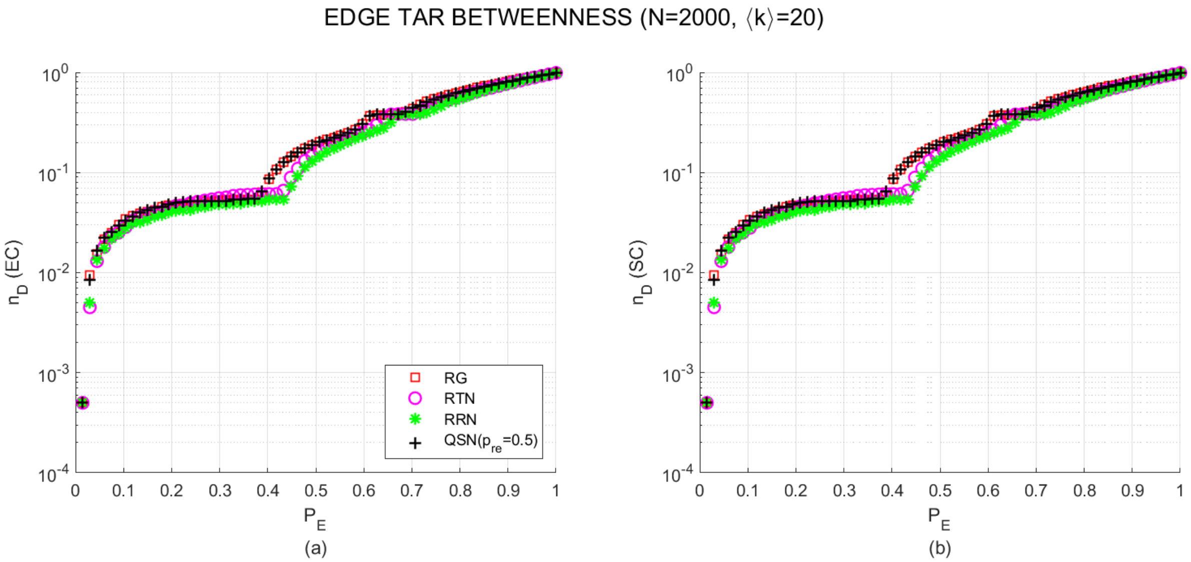

5.3.3 Edge Intentional (Degree-based) Attack

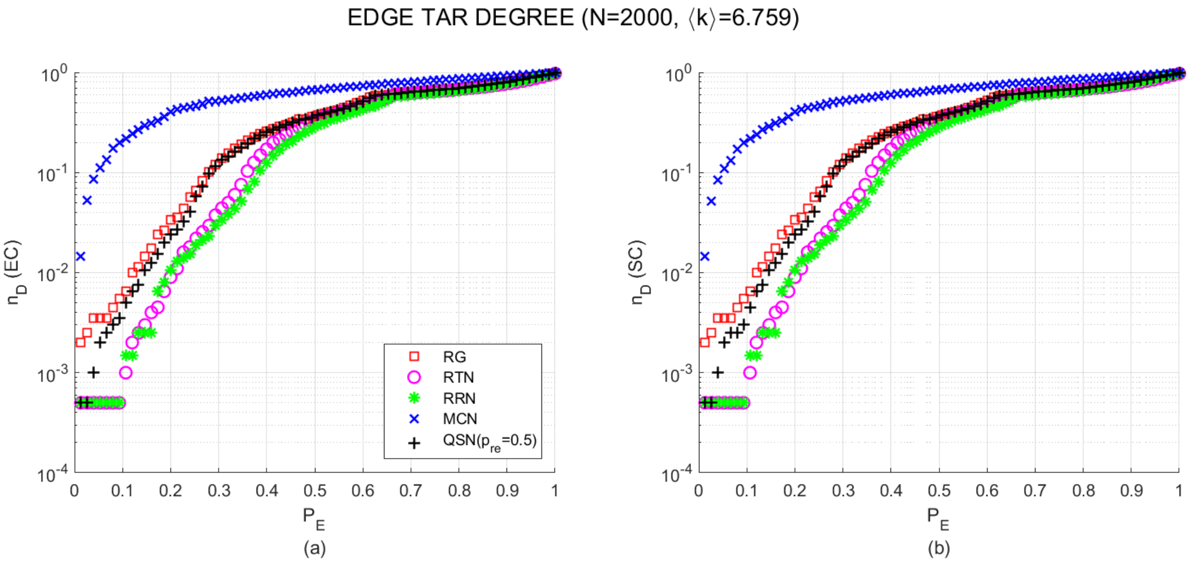

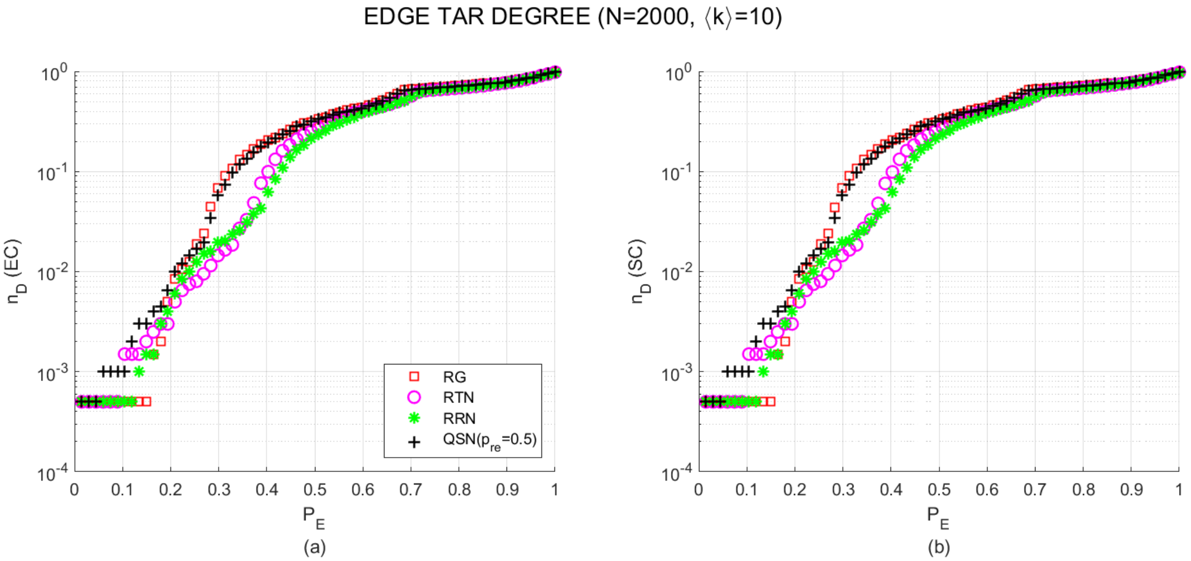

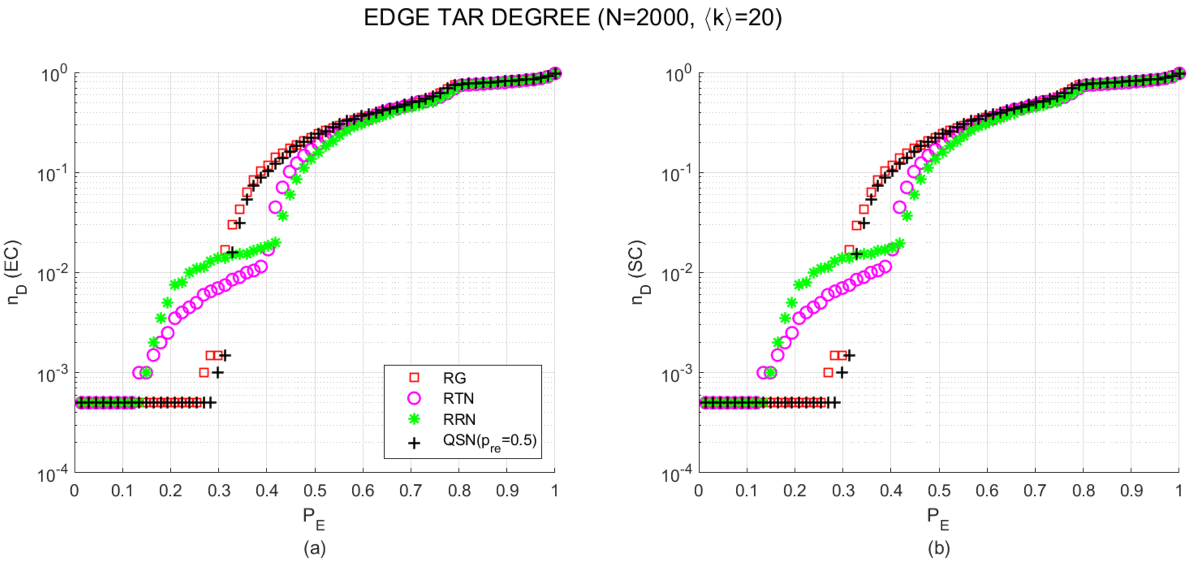

5.3.4 *Node Random Attack*

NODE RAND ATTACK (N=2000, ⟨k⟩=6.759)

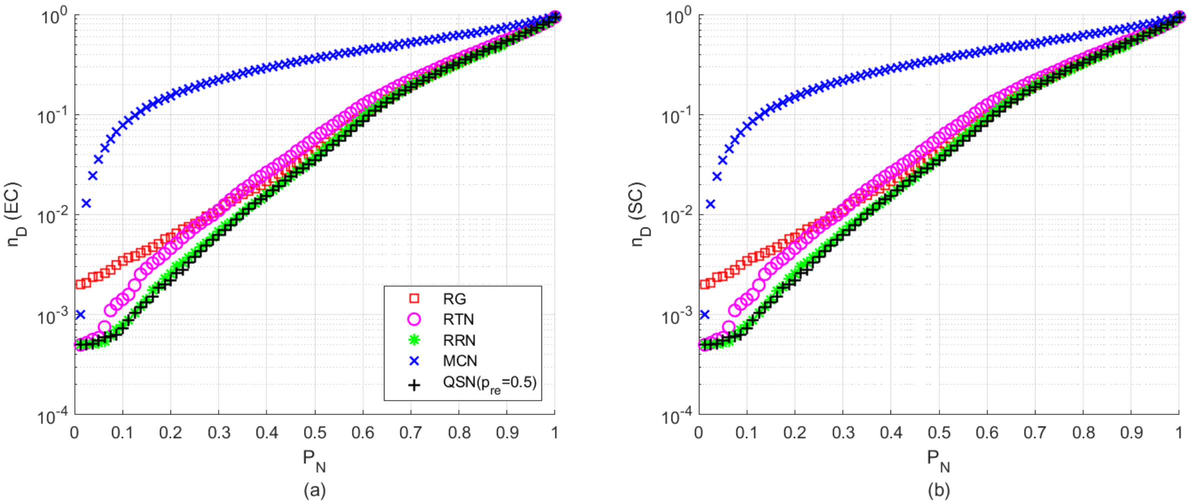

NODE RAND ATTACK (N=2000, ⟨k⟩=10)

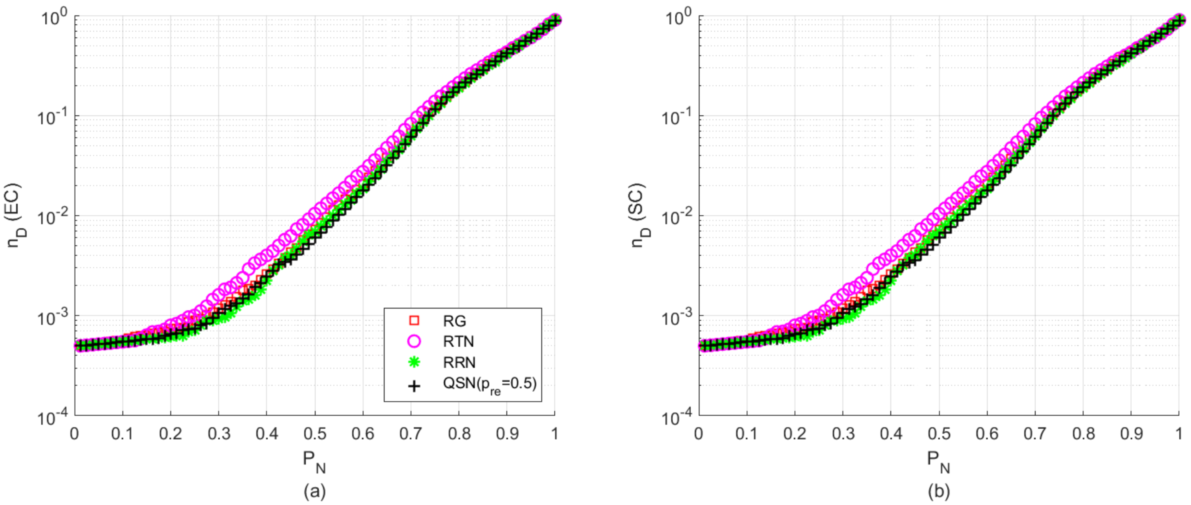

NODE RAND ATTACK (N=2000, ⟨k⟩=20)

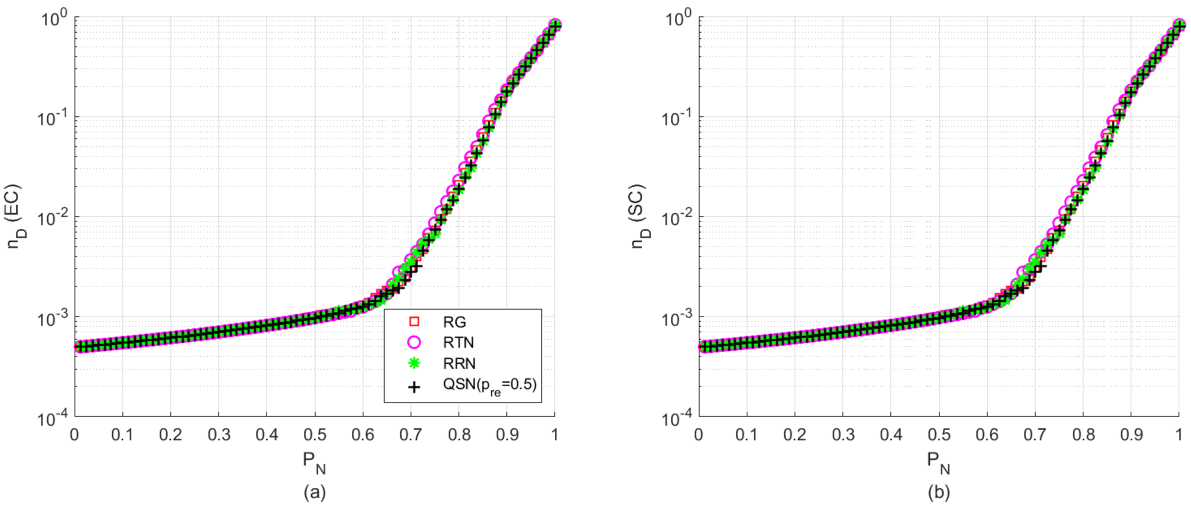

5.3.5 Node Intentional (Betweenness-based) Attack

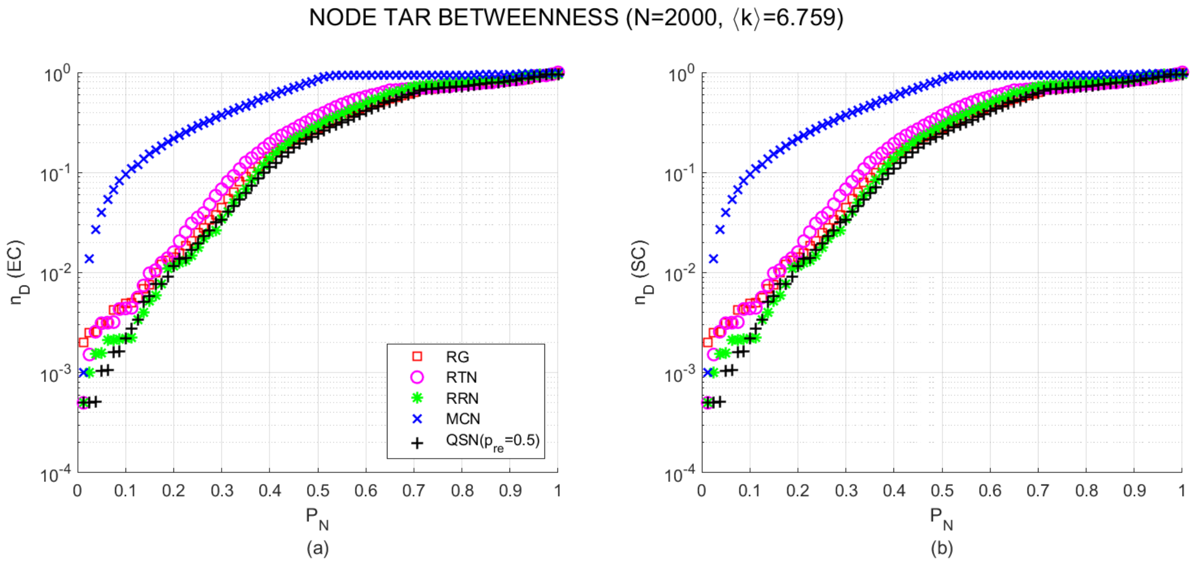

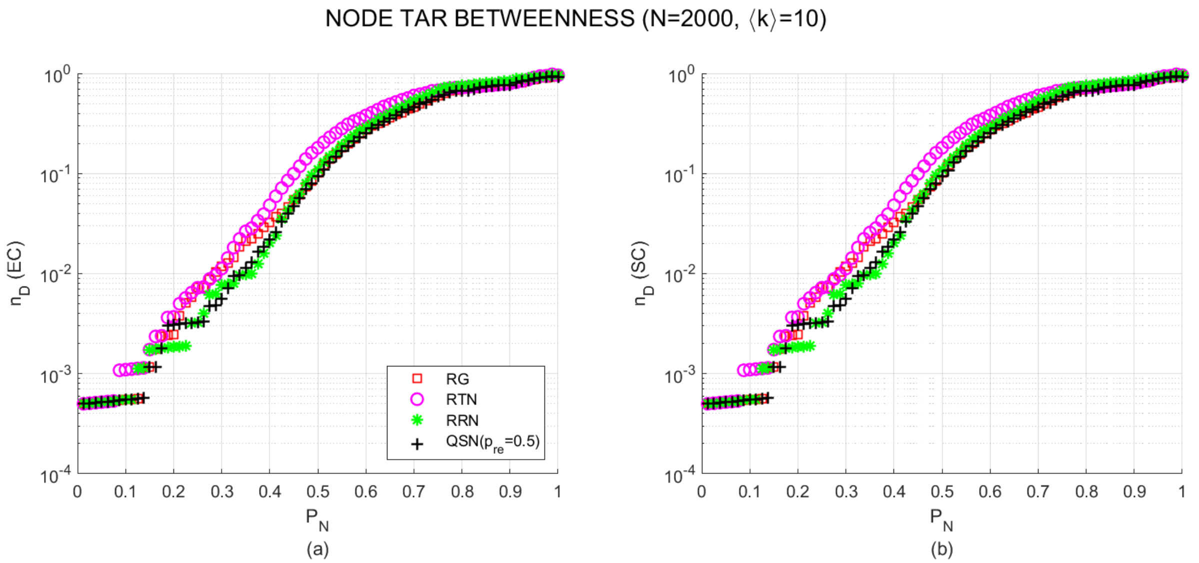

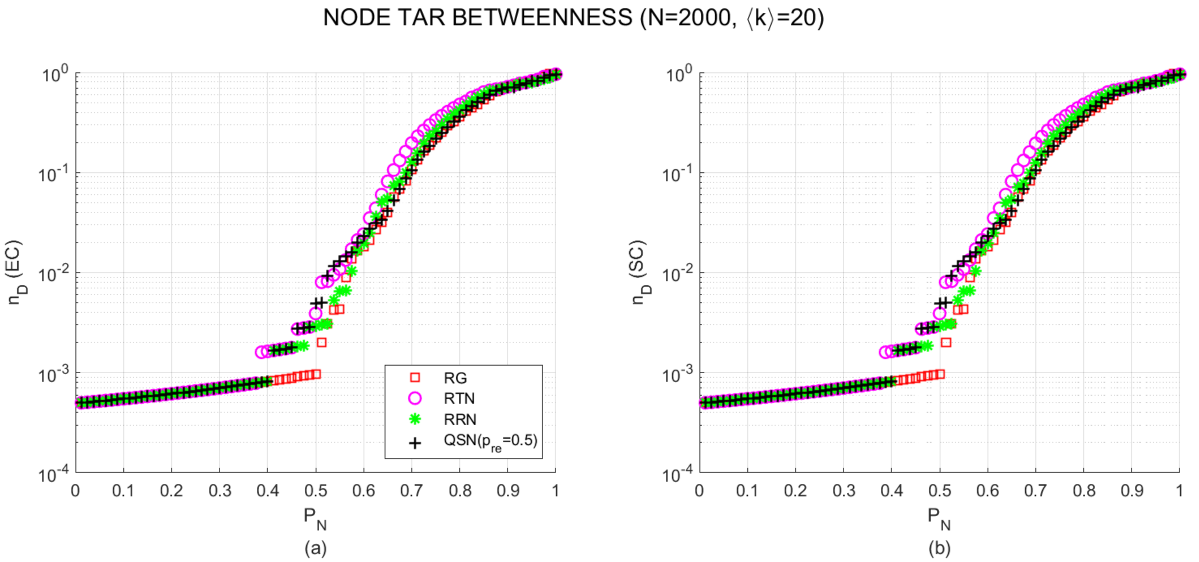

5.3.6 Node Intentional (Degree-based) Attack

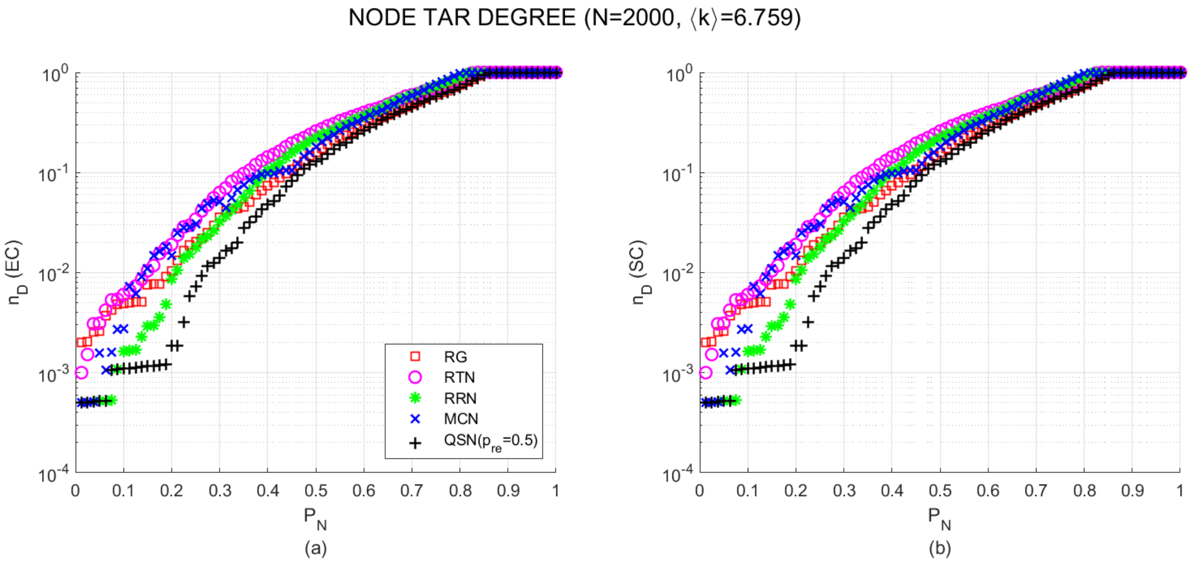

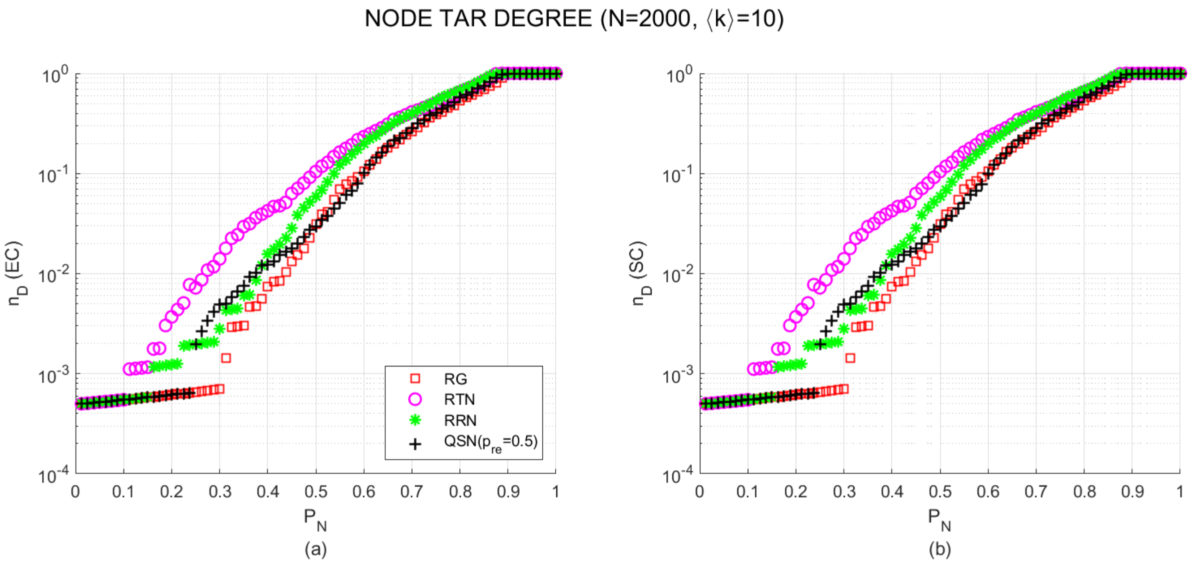

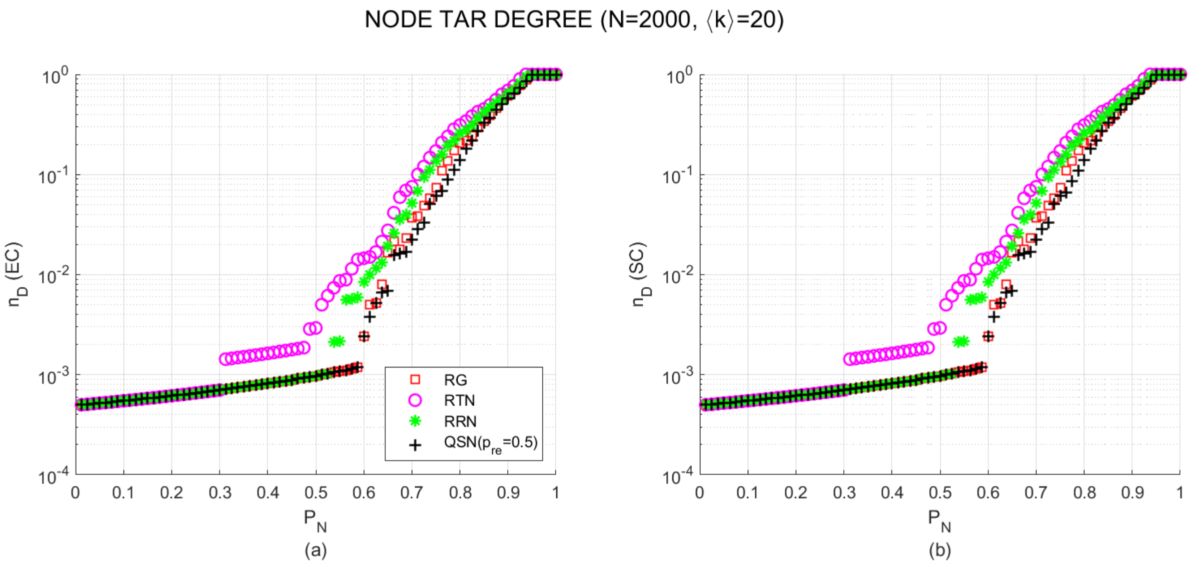

Supplement: Supplementary Materials — Supplemental information includes 32 tables and 97 figures can be found with this article online. [file 7857534.f1.pdf]
